# Genotype–covariate correlation and interaction disentangled by a whole-genome multivariate reaction norm model

**DOI:** 10.1038/s41467-019-10128-w

**Published:** 2019-05-20

**Authors:** Guiyan Ni, Julius van der Werf, Xuan Zhou, Elina Hyppönen, Naomi R. Wray, S. Hong Lee

**Affiliations:** 1 Australian Centre for Precision Health, University of South Australia Cancer Research Institute, University of South Australia, Adelaide, South Australia 5000 Australia; 2 School of Environmental and Rural Science, University of New England, Armidale, NSW 2351 Australia; 3 Population, Policy and Practice, UCL Great Ormond Street Institute of Child Health, London, WC1N 1EH UK; 4 South Australian Health and Medical Research Institute, Adelaide, South Australia 5000 Australia; 5 Institute for Molecular Bioscience, University of Queensland, Brisbane, Queensland 4072 Australia; 6 Queensland Brain Institute, University of Queensland, Brisbane, Queensland 4072 Australia

**Keywords:** Computational biology and bioinformatics, Genetics

## Abstract

The genomics era has brought useful tools to dissect the genetic architecture of complex traits. Here we propose a multivariate reaction norm model (MRNM) to tackle genotype–covariate (G–C) correlation and interaction problems. We apply MRNM to the UK Biobank data in analysis of body mass index using smoking quantity as a covariate, finding a highly significant G–C correlation, but only weak evidence for G–C interaction. In contrast, G–C interaction estimates are inflated in existing methods. It is also notable that there is significant heterogeneity in the estimated residual variances (i.e., variances not attributable to factors in the model) across different covariate levels, i.e., residual–covariate (R–C) interaction. We also show that the residual variances estimated by standard additive models can be inflated in the presence of G–C and/or R–C interactions. We conclude that it is essential to correctly account for both interaction and correlation in complex trait analyses.

## Abstract (web summary)

Complex traits are often influenced by genetic and non-genetic factors (such as environmental exposures), which are themselves interconnected. Here, the authors develop a method for disentangling genotype–covariate correlation and interaction, and investigate their effects on estimating statistical genetic parameters.

## Introduction

**Fig. 1:**
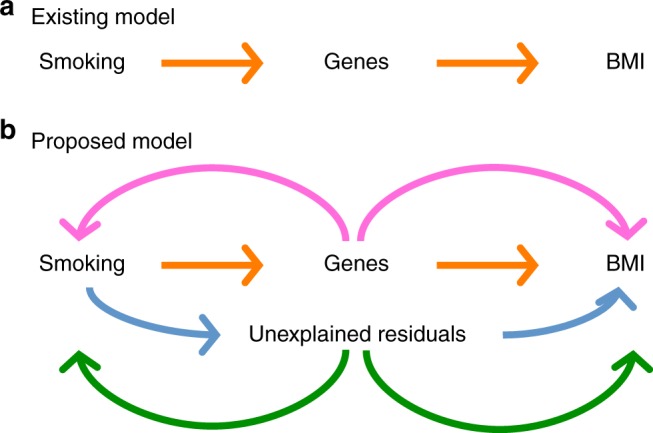
A simplified path diagram of the existing and proposed interaction model. **a** Existing interaction model where smoking modulates genes such that their expression, regulation and effects underlying BMI are changed (genotype–covariate interaction). **b** Proposed MRNM where genotype–covariate interaction is only a part of the latent mechanism that also includes pleiotropic effects on smoking and BMI (magenta arrow), residual–covariate interaction (blue arrow) and residual correlation (green arrow). Unexplained residuals may be partly due to epigenetic or unknown factors that are not captured by the genetic component, resulting in heterogeneous error (co)variance across different covariate values

Variation in complex traits between people is determined both by genetic and non-genetic factors. The non-genetic component will include environmental risk factors, but also unknown factors that are characterised by stochastic variation. The interplay between genetic and identifiable environmental factors has long been a topic of research interest^1–3^, since the identification of genotype–environment interactions has the potential to inform on health interventions to overcome genetic predisposition to disease. However, many so-called environmental risk factors (e.g., smoking, alcohol consumption, stressful life events, educational attainment) are themselves complex traits whose variation also reflects both genetic and non-genetic factors. For example, the relationship between smoking and body mass index (BMI) is complex, i.e., common causal genetic variants have biological effects on both traits (pleiotropy or genetic correlation)^4^ while BMI is also modulated by smoking status (interaction)^5–7^ (Fig. 1). The relationship between smoking and BMI is a good example for a complex association which can be best modelled using a framework that can account both for genotype–covariate correlation and interaction (GCCI).

Both correlation (‘association’) and interaction (‘effect modification’) are fundamental in biology^8–10^, but it is critical to distinguish between them because their biological mechanisms differ, as do their implications. This association/interaction problem has been well posed in the classical twin study approach^11^, showing that association and interaction can be disentangled and correctly estimated with an appropriate model and sufficient data. Unfortunately, large well-powered data sets with measures on multiple family members are few. However, genome-wide association studies (GWAS) now provide different types of genetically informative data to investigate GCCI. The genomic era has brought useful tools to dissect the genetic architecture of complex traits, where genetic variance and covariance can be estimated based on genome-wide single nucleotide polymorphisms (SNPs) genotyped in large-scale population samples. The increased availability of sufficiently powered data sets, with information on measured genetic and non-genetic risk factors, motivates the need to develop appropriate statistical tools for GCCI analysis. Locus specific interaction at single locus level has been widely studied^12^. However, it is desirable to estimate whole-genome level interaction, which is accumulated from every locus across the genome and has a direct implication on actual phenotypic modulations.

The reaction norm model (RNM) has been developed and applied to genotype–environment interaction analyses in ecology^13^ and agriculture^14,15^. The RNM allows environmental exposures to be modelled such that the genetic effects of a trait can be fitted as a nonlinear function of a continuous environmental gradient. The possible modulators of the phenotypes of the trait are not limited to environmental exposures, but can include any covariates, regulated by environmental and genetic factors, which are shared with the phenotypes. In other words, the genetic effect, and therefore the phenotype, of one trait often depends on the phenotype of another trait. This can be modelled by introducing dependence between the phenotype and the covariate, where the covariate represents the phenotype of the modulating trait, with both phenotypes having shared genetic and environmental components.

In the context of whole genome analyses of human complex traits, there is currently no approach that can fit GCCI effects to disentangle interaction from correlation at the genome-wide level. Yet, ignoring either the genotype–covariate (G–C) correlation or the G–C interaction may cause biased estimates of variance components which form the basis of SNP-heritability or interaction estimation^11^. Random regression-genomic restricted maximum likelihood (RR-GREML)^16,17^ and G–C interaction (GCI)-GREML^18^ have been used to detect and estimate G–C interaction at the whole genome level for BMI modulated by smoking quantity^16^. However, the analytical approach used in this study was based on univariate models which did not account for G–C correlation, thereby assuming that there is no correlation between the covariates and the outcomes. This can inflate signals indicating the presence of G–C interaction and lead to biased estimates by the failure to account for the G–C correlation. A further limitation with the existing methods is that these cannot be applied to continuous covariates without an arbitrary stratification into discrete exposure groups^16^. Importantly, additive models used for the estimation of SNP heritability (such as GREML^17,18^ which is based on individual level data, or LDSC^19–21^ based on summary statistics) may give biased estimates for genetic and residual (error) variance if the trait of interest is moderated by (unknown) covariates due to failure in adequate capture of the interaction effects. It is currently not possible to use RR-GREML or GCI-GREML to assess such bias especially when using continuous covariates.

In this study, we develop a whole-genome reaction norm model (RNM) that is computationally flexible and powerful when estimating genome-wide G–C interactions for complex traits. We also extend this approach to a whole-genome multivariate RNM (MRNM) framework to capture fully the GCCI effects, jointly modelling pleiotropy and interactions at the genome-wide level. As the proposed methods will be able to more realistically account for the complexity of GCCI effects, we hypothesise that they will lead to a significant reduction in bias and notably improve the estimation of the genetic architecture of complex traits.

## Results

### Overview of methods

We propose an extension of the whole-genome RNM that can estimate G–C interactions, where covariates can be continuous phenotypes of traits correlated with the response. In a simplified form of this model, the response variable (*y*) representing the main trait is modulated by a continuous covariate variable (*c*) as

$$y_i = g_i + e_i = \alpha _{i0} + \alpha _{i1} \cdot c_i + e_i$$

where *g*_*i*_ and *e*_*i*_ are the genetic and residual effects for the *i*^th^ individual record, and *g*_*i*_ can be further decomposed to the zero and first order random regression coefficients, denoted as *α*_*i*0_ and *α*_*i*1_ (i.e., the regression coefficients may vary between individuals) and *c*_*i*_ is the unique covariate value for the *i*^th^ individual (see Methods for the formal model specification and covariance structure). This model is the same as RR-GREML^16,17^ proposed by Maier et al.^22^ except the fact that *c*_*i*_ in our model is a continuous variable, as opposed to a discrete variable. The RNM can be generalised to account for residual heterogeneity or residual–covariate (R–C) interaction, by decomposing *e*_*i*_ as follows:

$$y_i = g_i + e_i = \alpha _{i0} + \alpha _{i1} \cdot c_i + \tau _{i0} + \tau _{i1} \cdot c_i$$

where *α*_*i*0_, *α*_*i*1,_ and *c*_*i*_ are defined as above, and *τ*_*i*0_ and *τ*_*i*1_ are the zero and first order of random regression coefficients for the residual variance (see Methods).

The RNM described above is used to model G–C and R–C interaction effects without accounting for G–C and R–C correlation. As briefly explained in Introduction section, the same genetic factors can affect both the covariate trait and the main trait (response variable), and at the same time, the covariate trait phenotypes can directly modify the main trait. For example, both BMI and smoking have non-zero SNP-based heritability^23^, there is a direct genetic association between BMI and smoking quantity, and BMI is known to be modulated by smoking. Typically, the covariate itself (here, smoking) is affected by genetic effects and residual error (i.e., *c*_*i*_ = *β*_*i*_ + *ε*_*i*_), and there can be non-negligible correlations between *α*_0_ and *β*, *α*_1_ and *β, τ*_0_ and *ε*, and *τ*_1_ and *ε* (for the full covariance structure, see Methods). We used MRNM to take into account the G–C and R–C correlation.

We compared the performance of previously published methods (RR-GREML^16,17^ and GCI-GREML^18^), RNM, and MRNM using simulated and real data from the UK Biobank^24^ (see Methods and Supplementary Note 1). The models used in these comparisons are summarised in Supplementary Table 1. In the simulation, we used likelihood ratio tests to obtain the *p*-values, and assessed bias, type I error rate and power of detecting G–C and R–C interactions. In the analyses using the UK Biobank, we modelled BMI as the main trait and fitted separate models using information on pack years of smoking (SMK), neuroticism score (NEU) and the first principal component of genotypes (PC1) as the covariates. We used SMK and NEU because of their well-known association with BMI^16,25,26^ although the variance and covariance components of the interaction effects were not clearly known. We would expect to see little or no evidence for interaction due to PC1 because BMI was reported to have relatively small interaction across different populations^27^ and the data used in this study were stringently restricted according to their ancestry (see Methods). In addition, we applied the standard GREML^17,18^ and LDSC^19–21^ methods to estimate SNP-based heritability for the main response variable (*y*), where *y* is modulated by one or more unknown covariates. With this analysis, we are able to explore the potential bias in results obtained by these methods in the presence of non-negligible interactions.

### Type I error rate, power and estimates for G–C interaction

**Fig. 2:**
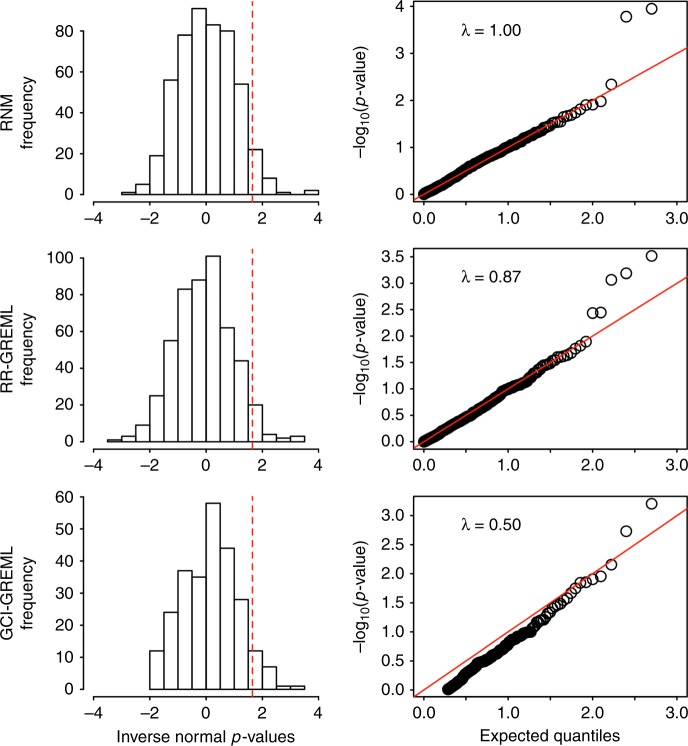
Type I error rate for detecting G–C interaction is under control. Five hundred replicates of data were simulated under a null model that assumed no genotype–covariate interaction. Simulation was based QCed ARIC data consisting 7,263 individuals and 583,058 SNPs. The model is specified as **y** = **α**_**0**_  **+ α**_**1**_ × **c** + **e** with **c** = **β** + **ε**, all effects drawn from a multivariate normal distribution, where the variance–covariance structure between **α**_**0**_, **β**, and **α**_**1**_ (in this order) is $$\left[ {\begin{array}{*{20}{c}} 1 & 0 & 0 \\ 0 & 1 & 0 \\ 0 & 0 & 0 \end{array}} \right]$$ and that between **e** and **ε** is $$\left[ {\begin{array}{*{20}{c}} 1 & 0 \\ 0 & 1 \end{array}} \right]$$. For every replicate, each of the three models was fitted to obtain a *p*-value for the G–C interaction via a comparison between the null (H_0_) and alternative hypothesis (H_1_) models using a likelihood ratio test. For RNM, the H_0_ and H_1_ models were **y** **=** **α**_**0**_ + **e** and **y** **=** **α**_**0**_ + **α**_**1**_ × **c** **+** **e**. For RR-GREML and GCI-GREML, the H_0_ and H_1_ models were **y** **=** **α**_**0**_ + **e** and **y** **=** **α**_**0**_ + **α**_**1**_ × **c** **+** **e**. In RR-GREML and GCI-GREML, samples were arbitrarily stratified into four different groups according to the covariate levels. RR-GREML explicitly estimate residual variance for each of the four groups whereas GCI-GREML assumes homogeneous residual variance across the four groups and estimates a single residual variance. The left panels show the proportions of significant *p*-values, i.e., type I error rate, for RNM, RR-GREML and GCI-GREML, which are 0.048, 0.048 and 0.034, respectively. Note that *p*-values are inverse normal transformed, such that the statistical significance level, i.e., 1.65, shown as dashed lines, is equivalent to the 0.05 level before the transformation. The right panels are quantile–quantile (Q–Q) plots of −log_10_(*p*-values) from RNM, RR-GREML and GCI-GREML to detect G–C interaction using simulated data (λ is the ratio of observed to expected median test statistic). The low lambda values for RR-GREML and GCI-GREML was probably due to the fact that the methods stratify individuals into multiple groups. In addition, GCI-GREML constrains the negative estimated variance to zero

**Fig. 3:**
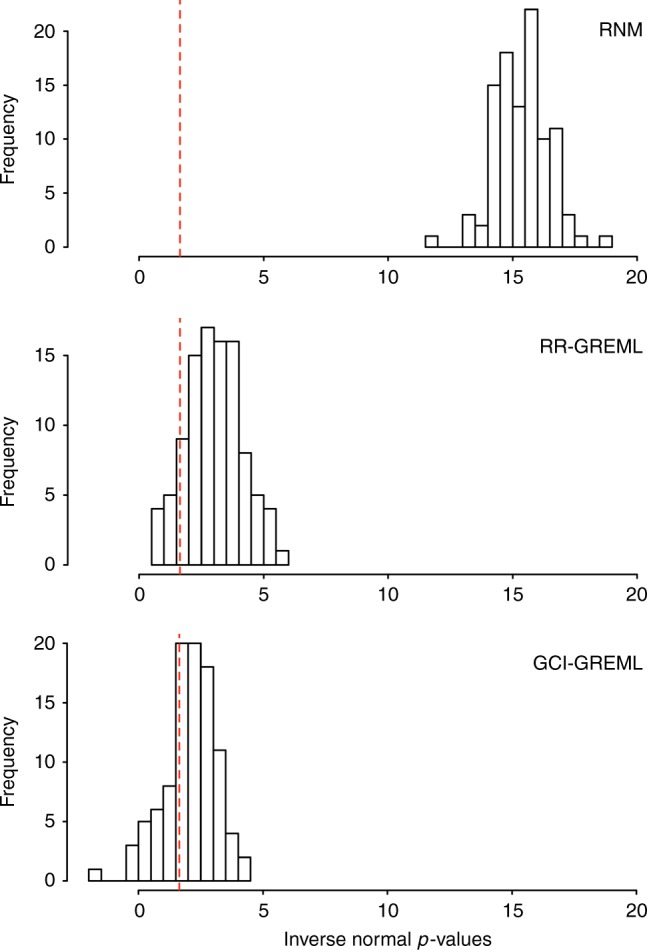
RNM has more statistical power than RR-GREML and GCI-GREML. One hundred replicates of data were simulated under a model that assumed the presence of a genotype–covariate interaction. Simulation was based QCed ARIC data consisting 7,263 individuals and 583,058 SNPs. The model is specified as **y** **=** **α**_**0**_ + **α**_**1**_ × **c** **+** **e** with **c** **=** **β** **+** **ε**, all effects drawn from a multivariate normal distribution, where the variance–covariance structure between **α**_**0**_, **β**, and **α**_**1**_ (in this order) is $$\left[ {\begin{array}{*{20}{c}} 1 & 0 & {0.05} \\ 0 & 1 & 0 \\ {0.05} & 0 & {0.25} \end{array}} \right]$$ and that between **e** and **ε** is $$\left[ {\begin{array}{*{20}{c}} 1 & 0 \\ 0 & 1 \end{array}} \right]$$. For every replicate, each of the three models was fitted to obtain a *p*-value for the G–C interaction via a comparison between the null (H_0_) and alternative hypothesis (H_1_) models using a likelihood ratio test. For RNM, the H_0_ and H_1_ models were **y** **=** **α**_**0**_ + **e** and **y** **=** **α**_**0**_  **+ α**_**1**_ × **c** **+** **e**. For RR-GREML and GCI-GREML, the H_0_ and H_1_ models were **y** **=** **α**_**0**_ + **e** and **y** **=** **α**_**0**_  **+ α**_**1**_ × **c** **+** **e**. In RR-GREML and GCI-GREML, samples were arbitrarily stratified into four different groups according to the covariate levels. RR-GREML explicitly estimate residual variance for each of the four groups whereas GCI-GREML assumes homogeneous residual variance across the four groups and estimates a single residual variance. This figure shows the proportions of significant *p*-values, i.e., statistical power, for RNM, RR-GREML and GCI-GREML, which are 1, 0.9 and 0.69, respectively. Note that *p*-values are inverse normal transformed, such that the statistical significance level, i.e., 1.65, shown as dashed lines, is equivalent to the 0.05 level before the transformation

We used simulation (see Methods) to quantify type I error rate and power of detecting G–C interaction for the proposed RNM, RR-GREML and GCI-GREML, without considering G–C correlation. As shown in Fig. 2, there was no inflation of type I error rate for all methods under the null model, when there was no G–C correlation and interaction. In contrast, when there were non-negligible G–C interactions, RNM outperformed RR-GREML and GCI-GREML in detecting G–C interactions (Fig. 3). The power to detect G–C interaction was slightly higher for RR-GREML compared to GCI-GREML. The type I error rate and power of MRNM were very similar to those of RNM (Supplementary Fig. 1).

We also tested if the methods can give unbiased estimates for variance components of random regression coefficients underlying the mechanism of G–C interaction. When G–C interactions were present, RNM gave unbiased estimates, whereas estimates from RR-GREML and GCI-GREML differed from true values (Supplementary Table 2). Note that RR-GREML and GCI-GREML required the stratification of the sample into discrete groups, resulting in an artificial heterogeneity of phenotypic variances across the discrete groups (Supplementary Fig. 2).

### Type I error rate, power and estimates for GCCI

**Fig. 4:**
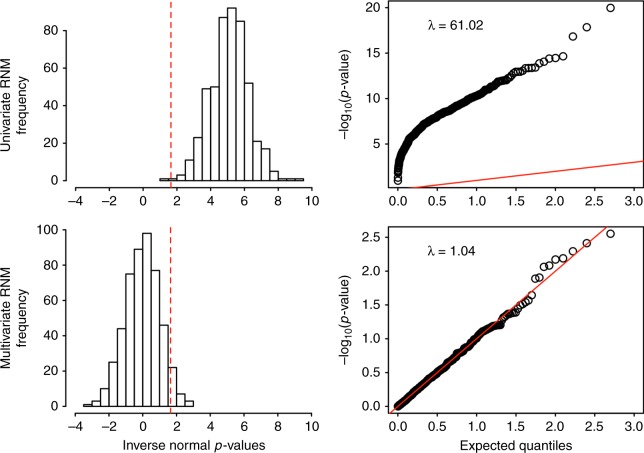
Spurious signals generated by incorrect (univariate) model can be controlled by MRNM. Five hundred replicates of data were simulated under a null model that assumed genotype–covariate correlation but no genotype–covariate interaction. Simulation was based QCed ARIC data consisting 7,263 individuals and 583,058 SNPs. The model is specified as **y** **=** **α**_**0**_ + **α**_**1**_ × **c** **+** **e** with **c** = **β** **+** **ε**, all effects drawn from a multivariate normal distribution, where the variance–covariance structure between **α**_**0**_, **β**, and **α**_**1**_ (in this order) is $$\left[ {\begin{array}{*{20}{c}} 1 & {0.5} & 0 \\ {0.5} & 1 & 0 \\ 0 & 0 & 0 \end{array}} \right]$$ and that between **e** and **ε** is $$\left[ {\begin{array}{*{20}{c}} 1 & {0.3} \\ {0.3} & 1 \end{array}} \right]$$. For every replicate, a univariate RNM and a multivariate RNM were fitted separately to obtain a *p*-value for the G–C interaction by comparing the null (H_0_) and alternative hypothesis (H_1_) model using a likelihood ratio test. For the univariate RNM, the H_0_ and H_1_ models were **y** **=** **α**_**0**_ + **e** and **y** **=** **α**_**0**_ + **α**_**1**_ × **c** **+** **e**. For the multivariate RNM, the H_0_ and H_1_ models were **y** **=** **α**_**0**_ + **e** with **c** **=** **β** **+** **ε** and **y** **=** **α**_**0**_ + **α**_**1**_ × **c** **+** **e** with **c** **=** **β** **+** **ε**. The left panel shows the proportions of significant *p*-values, i.e., type I error rate, for both models, which are 0.998 (univariate RNM) and 0.046 (multivariate RNM). Note that *p*-values are inverse normal transformed, such that the statistical significance level, i.e., 1.65, shown as dashed lines, is equivalent to the 0.05 level before the transformation. The right panel is quantile-quantile (Q-Q) plots of −log10(*p*-values) from univariate RNM and multivariate RNM. λ is the ratio of observed to expected median test statistic, which is inflated when using an inappropriate model

We also considered the GCCI model in simulations (Methods). Under the null (no G–C interaction), in the presence of non-negligible genetic correlations between the main response and covariate variables, we observed spurious signals for G–C interaction in the univariate analysis using the RNM (Fig. 4). This was probably due to the fact that the unmodelled association of the main genetic effects between the phenotypes and covariate, cov(**α**_0_, **β**), was partly captured and estimated as interaction variance, var(**α**_1_ · **c**), from the model, **y** = **α**_0_ + **α**_1_ · **c** + **e**. However, MRNM performed notably better in these analyses, being able to control for type I error rate (0.046) in detecting G–C interaction (Fig. 4). With a more modest genetic correlation (e.g., 0.1), type I error rate was still inflated (0.25 in Supplementary Fig. 3) and the estimates were biased (Supplementary Table 3) when applying the univariate model.

In the presence of G–C correlations and G–C interactions, both RNM and MRNM performed similarly in detecting G–C interactions (Supplementary Fig. 4) although the significance of the G–C interaction for RNM was slightly inflated due to over-estimated parameters (see var(**α**_1_) for RNM in Supplementary Table 4). Importantly, MRNM gave unbiased estimates for both G–C correlation and G–C interaction (Supplementary Table 4).

When using RNM, the spurious signals for detecting G–C interaction could be controlled by adjusting the main response for the covariate, i.e., using residuals (as the response) from the regression of the main response on the covariate (Supplementary Figs. 5 and 6). However, such adjustment was crude, and the genuine effects were sometimes over-corrected, again leading to biased estimates especially in the estimated variance of the main effects (Supplementary Table 5).

### Residual–covariate (R–C) correlation and interaction

In addition to GCCI, it is possible that the residual effects (*e*_*i*_) are correlated with the covariate (*c*_*i*_) and that there is interaction (RCCI) (see Eq. (3) or Methods). We tested various scenarios for detecting G–C interactions in the presence of R–C correlation and/or interaction (Supplementary Figs. 7–11). In the absence of G–C interactions but with R–C interactions, type I error rate was well controlled in all methods (Supplementary Fig. 7 and Supplementary Data 1). In the presence of G–C interactions and R–C interactions, RNM had greater power to detect G–C interaction compared to RR-GREML or GCI-GREML (Supplementary Fig. 8 and Supplementary Data 2). In the absence of G–C interaction but with G–C correlations and RCCI, all three methods were able to control type I errors in detecting G–C interaction (Supplementary Fig. 9 and Supplementary Data 3). With the full GCCI model in the presence of G–C correlation and interaction, and R–C correlation and interaction, MRNM had greater power than RR-GREML or GCI-GREML (Supplementary Fig. 10 and Supplementary Data 4). When increasing the variance explained by the G–C interaction, the statistical power reached 100% with all three methods (Supplementary Fig. 11 and Supplementary Data 5). It is notable that MRNM gave unbiased estimates of the components whereas the other methods generated some degree of bias in the estimation (Supplementary Data 3–5, and Supplementary Table 6).

### Inflated residual variance using LDSC or GREML

**Fig. 5:**
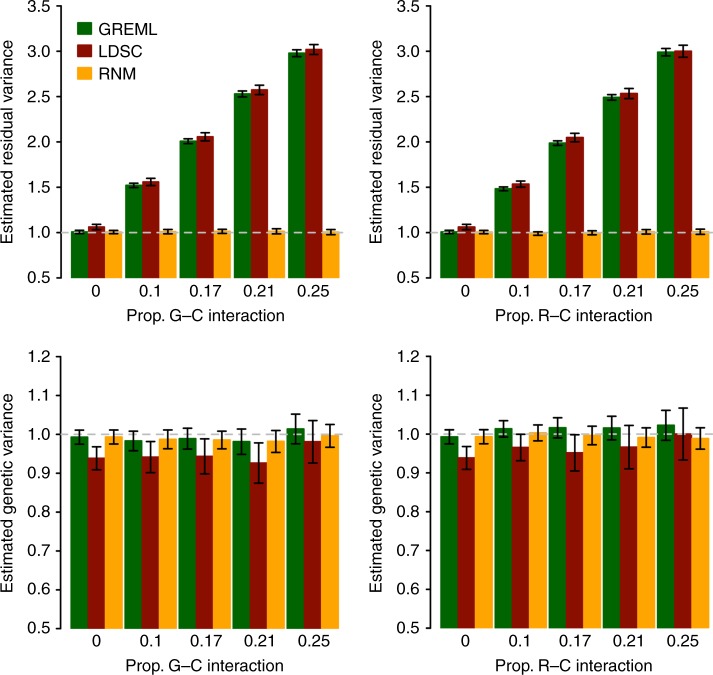
Estimated residual and genetic variances from data simulated under interaction models. Prop. G–C or R–C interaction is the proportion of variance due to **α**_**1**_ or **τ**_**1**_ (see below) in the total phenotypic variance (i.e., var(**α**_**1**_)/var(**y**) in G–C interaction model or var(**τ**_**1**_)/var(**y**) in R–C interaction model). Simulation for G–C interaction (**α**_**1**_) (left panels): The phenotype data were generated using **y** **=** **α**_**0**_ + **α**_**1**_ × **c** **+** **e** with **c** **=** **β** **+** **ε**, all effects drawn from a multivariate normal distribution. The variance–covariance structure of **α**_**0**_, **β**, and **α**_**1**_ (in this order) is $$\left[ {\begin{array}{*{20}{c}} 1 & 0 & {0.05} \\ 0 & 1 & 0 \\ {0.05} & 0 & {{\mathrm{var}}({\mathbf{\alpha }}_1)} \end{array}} \right]$$ with var(**α**_**1**_) = 0, 0.25, 0.5, 0.75 and 1 and that for **e** and **ε** is $$\left[ {\begin{array}{*{20}{c}} 1 & 0 \\ 0 & 1 \end{array}} \right].$$ Simulation for R–C interaction (**τ**_**1**_) (right panels): The phenotype data were generated using **y** **=** **α**_**0**_  + **τ**_**0**_  + **τ**_**1**_ × **c** with **c** **=** **β** **+** **ε**, all effects drawn from a multivariate normal distribution. The variance–covariance structure of **α**_**0**_ and **β** is $$\left[ {\begin{array}{*{20}{c}} 1 & 0 \\ 0 & 1 \end{array}} \right]$$ and that of **τ**_**0**,_ **ε**, and **τ**_**1**_ (in this order) is $$\left[ {\begin{array}{*{20}{c}} 1 & 0 & {0.05} \\ 0 & 1 & 0 \\ {0.05} & 0 & {{\mathrm{var}}({\mathbf{\tau }}_1)} \end{array}} \right]$$ with var(**τ**_**1**_) = 0, 0.25, 0.5, 0.75 and 1. The error bar is a 95% confidence interval, which was estimated over 100 replicates. The model for GREML is **y** = **α**_**0**_  **+ e** and the model for RNM in the left panel is **y** **=** **α**_**0**_  **+ α**_**1**_ × **c** **+** **e**. The model for RNM in the right panel is **y** **=** **α**_**0**_  **+ τ**_**0**_ **+** **τ**_**1**_ × **c**. Simulation was based QCed ARIC data consisting 7,263 individuals and 583,058 SNPs

LDSC SNP-based heritability estimates have been widely used^19,20,28^. However, it is not clear if G–C or R–C interactions have an effect on LDSC SNP-based heritability estimations. When using LDSC, GWAS summary stats are typically used without knowing (accessing) the information of specific covariates. So, it is important to assess the biasness of estimates if interaction effects are not properly modelled either because of the model limitation (i.e., LDSC is an additive model) or lack of covariate information. With simulated data based on the G–C or R–C interaction model, we showed that both the GREML and LDSC overestimated the residual variance for the main response variable hence underestimating the SNP-based heritability (Fig. 5 and Supplementary Fig. 12). When the interaction component explained 10% of the total variance, the estimated residual variance based on GREML or LDSC was 1.5 times higher than the true simulated value (Fig. 5). When the variance of the interaction was increased to 25% of the total variance, GREML or LDSC overestimated the residual variance up to 3-fold. However, RNM generated unbiased estimates for the residual variance in most cases. It was noted that the estimated genetic variance and covariance were mostly unbiased whether using GREML, LDSC or RNM (Fig. 5 and Supplementary Table 7). This implies that a consistent estimate of SNP-based heritability can be obtained across the methods given that the phenotypic variance is correctly estimated on the original scale, which is invariant to whether there are interactions or not.

### GCCI and RCCI analysis for real data (UK Biobank)

We used the first release of UK Biobank (see Methods) to compare various models that test interaction using RR-GREML (M1) and GCI-GREML (M2), and the proposed approaches RNM (M3-M7) and MRNM (M8-M12) (Table 1). We applied the models with BMI as the outcome trait using either SMK, NEU or PC1 as the covariate of interest, in order to detect G–C and/or R–C interactions.

**Table 1:** *P*-values of likelihood ratio tests for model comparisons in UK Biobank analyses of BMI

Index	Model comparison	Model equation	SMK^a^	NEU^b^	PC1^c^
Univariate models					
M1	H_0_: RR-GREML k = 0^d^	**T**_1_ = **α**_0_ +**e**	1.00E-03	6.28E-04	7.00E-01
	H_1_: RR-GREML k = 1^d^	**T**_1_ = **α**_0_ + **α**_1_ · **c** +**e**			
M2	H_0_: Uni-GREML	**T**_1_ = **α**_0_ +**e**	1.99E-07	6.18E-01	1.00E-00
	H_1_: GCI-GRMEL	**T**_1_ = **α**_0_ + **α**_1_ · **c** + **e**			
M3	H_0_: Uni-GREML	**T**_1_ = **α**_0_ + **e**	1.89E-49	1.05E-49	8.39E-01
	H_1_: RNM Full	**T**_1_ = **α**_0_ + **α**_1_ · **c** + **τ**_0_ + **τ**_1_ · **c**			
M4	H_0_: Uni-GREML	**T**_1_ = **α**_0_ + **e**	8.76E-48	2.36E-48	5.63E-01
	H_1_: RNM R–C	**T**_1_ = **α**_0_ + **τ**_0_ + **τ**_1_ · **c**			
M5	H_0_: Uni-GREML	**T**_1_ = **α**_0_ + **e**	1.19E-44	1.15E-46	5.02E-01
	H_1_: RNM G–C	**T**_1_ = **α**_0_ + **α**_1_ · **c** + **e**			
M6	H_0_: RNM G–C	**T**_1_ = **α**_0_ + **α**_1_ · **c** + **e**	1.35E-07	7.73E-06	9.74E-01
	H_1_: RNM Full	**T**_1_ = **α**_0_ + **α**_1_ · **c** + **τ**_0_ + **τ**_1_ · **c**			
M7	H_0_: RNM R–C	**T**_1_ = **α**_0_ + **τ**_0_ + **τ**_1_ · **c**	1.83E-04	3.77E-04	8.69E-01
	H_1_: RNM Full	**T**_1_ = **α**_0_ + **α**_1_ · **c** + **τ**_0_ + **τ**_1_ · **c**			
Multivariate models					
M8	H_0_: MVGREML	**T**_1_ = **α**_0_ + **e** **T**_2_ = **β** + **ε**	1.97E-135	4.12E-48	8.98E-01
	H_1_: MRNM Full	**T**_1_ = **α**_0_ + **α**_1_ · **c** + **τ**_0_ + **τ**_1_ · **c** **T**_2_ = **β** + **ε**			
M9	H_0_: MVGREML	**T**_1_ = **α**_0_ + **e** **T**_2_ = **β** + **ε**	6.10E-137	2.18E-47	7.09E-01
	H_1_: MRNM R–C	**T**_1_ = **α**_0_ + **τ**_0_ + **τ**_1_ · **c** **T**_2_ = **β** + **ε**			
M10	H_0_: MVGREML	**T**_1_ = **α**_0_ + **e** **T**_2_ = **β** + **ε**	2.93E-101	1.17E-45	7.09E-01
	H_1_: MRNM G–C	**T**_1_ = **α**_0_ + **α**_1_ · **c** + **e** **T**_2_ = **β** + **ε**			
M11	H_0_: MRNM G–C	**T**_1_ = **α**_0_ + **α**_1_ · **c** + **e** **T**_2_ = **β** + **ε**	2.37E-37	2.36E-05	8.39E-01
	H_1_: MRNM Full	**T**_1_ = **α**_0_ + **α**_1_ · **c** + **τ**_0_ + **τ**_1_ · **c** **T**_2_ = **β** + **ε**			
M12	H_0_: MRNM R–C	**T**_1_ = **α**_0_ + **τ**_0_ + **τ**_1_ · **c** **T**_2_ = **β** + **ε**	3.26E-02	1.08E-03	8.40E-01
	H_1_: MRNM Full	**T**_1_ = **α**_0_ + **α**_1_ · +**τ**_0_+**τ**_1_ · c **T**_2_ = **β** + **ε**			

Note: T_1_ is the residual of main trait adjusted for confounders. T_2_ is the residual of c adjusted for confounders

^a^SMK: Pack years of smoking used in BMI-SMK interaction analysis

^b^NEU: Neuroticism score treated as continuous variable used in BMI–NEU interaction analysis

^c^The first principal component provided by UK Biobank used in BMI–PC interaction analysis

^d^Samples used in the respective model were arbitrarily stratified into four different levels according to covariates, SMK, NEU and PC1. Residual variance was estimated in each level for RR-GREML whereas GCI-GREML assumes homogeneous residual variance across the four groups and estimates a single residual variance

**Fig. 6:**
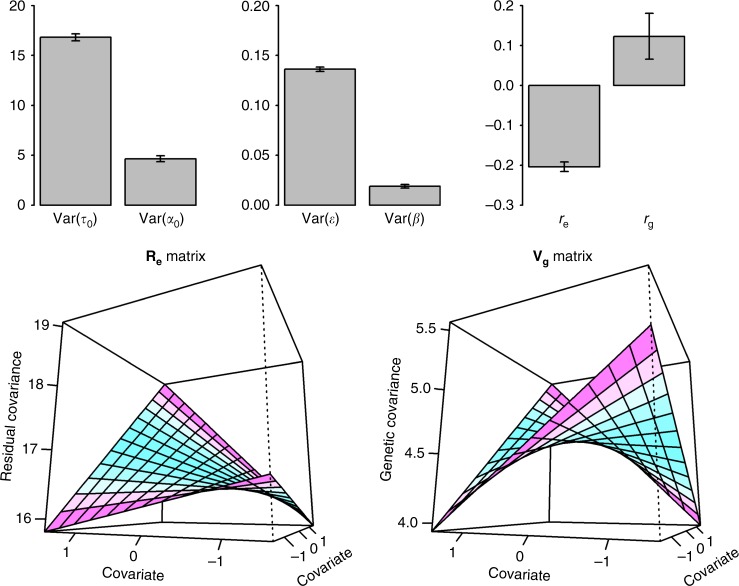
Estimated variance components and correlations from MRNM for BMI-SMK analysis. Var(**τ**_0_): Estimated residual variance for BMI as the main outcome. Var(**α**_0_): Estimated genetic variance for BMI as the main outcome. Var(**ε**): Estimated residual variance for SMK as the covariate. Var(**β**): Estimated genetic variance for SMK as the covariate. r_e_: Estimated residual correlation between BMI and SMK. r_g_: Estimated genetic correlation between BMI and SMK. Error bars are 95% confidence interval. **R**_**e**_ matrix is the residual (co)variance structure between different covariate levels (see Eq. 4), which is derived using estimated random regression coefficients and polynomial matrix as **R**_**e**_ = **ΦM**_**y**_**Φ**′. **Φ** is the matrix of polynomials evaluated at given covariate values, where entries of the first column are all **1**s and the second column is the standardised covariates of respective individuals. **M**_**y**_ is the variance–covariance matrix of estimated random regression coefficients from MRNM as $${\mathbf{M}}_{\mathbf{y}} = \left[ {\begin{array}{*{20}{c}} {{\mathrm{var}}({\mathbf{\tau }}_0)} & {{\mathrm{cov}}({\mathbf{\tau }}_0,{\mathbf{\tau }}_1)} \\ {{\mathrm{cov}}({\mathbf{\tau }}_0,{\mathbf{\tau }}_1)} & {{\mathrm{var}}({\mathbf{\tau }}_1)} \end{array}} \right] = \left[ {\begin{array}{*{20}{c}} {16.81({\mathrm{SE}}0.18)} & {0.41({\mathrm{SE}}0.12)} \\ {0.41({\mathrm{SE}}0.12)} & {0.42({\mathrm{SE}}0.16)} \end{array}} \right]$$. **V**_**g**_ matrix in is the genetic (co)variance structure between different covariate levels (see Eq. 2), which is derived based on the estimated random regression coefficients and polynomial matrix as **V**_**g**_ = **ΦK**_**y**_**Φ**′. **Φ** is the matrix of polynomials evaluated at given covariate values, where entries of the first column are all **1**s and the second column is the standardised covariates of respective individuals. **K**_**y**_ is the variance–covariance matrix of random regression coefficients estimated from MRNM as $${\mathbf{K}}_{\mathbf{y}} = \left[ {\begin{array}{*{20}{c}} {{\mathrm{var}}({\mathbf{\alpha }}_0)} & {{\mathrm{cov}}({\mathbf{\alpha }}_0,{\mathbf{\alpha }}_1)} \\ {{\mathrm{cov}}({\mathbf{\alpha }}_0,{\mathbf{\alpha }}_1)} & {{\mathrm{var}}({\mathbf{\alpha }}_1)} \end{array}} \right] = \left[ {\begin{array}{*{20}{c}} {4.66({\mathrm{SE}}0.15)} & {0.07({\mathrm{SE}}0.10)} \\ {0.07({\mathrm{SE}}0.10)} & {0.30({\mathrm{SE}}0.12)} \end{array}} \right]$$

Table 1 shows the *p*-values for interaction effects from the likelihood ratio tests and the corresponding estimates for variance and covariance components are presented in Supplementary Data 6. We found that BMI was significantly modulated by SMK using RR-GREML (M1, *p*-value = 1.00E-03) or GCI-GREML (M2, *p*-value = 1.99E-07), confirming published results^16^. However, published methods did not account for G–C correlation or RCCI (Supplementary Data 4 and 5). Using RNM (M3-M7), we found that the combined G–C and R–C interaction effects were highly significant (M3-M5). We then used RNM to test for the G–C interaction corrected for R–C interaction (M7) and found similar results (*p*-value = 1.83E-04 and var(**α**_1_) = 0.47 with SE = 0.12) compared to those obtained using RR-GREML (M1) and GCI-GREML (M2). It is noted that residual heterogeneity (reflected by R–C interaction) was partly controlled in M1 and M2 as these models adjusted for group differences with the covariate stratified into four discrete groups, which however generated biased estimates as shown in Supplementary Data 4 and 5 from simulation. We next applied MRNM to test for interactions, accounting for both G–C interaction and G–C correlation effects (M8-M12). We found that the signal for the combined G–C and R–C interaction increased (M8-M10) compared to that seen using RNM, which turned out to be mostly due to the increased R–C interaction (M11). It is likely that this was due to the large negative residual correlation between BMI and SMK (Fig. 6 and Supplementary Data 6) which could be more properly modelled in MRNM than in RNM. We finally tested G–C interaction controlled for G–C correlation, and R–C correlation and R–C interaction (M12), and showed that the signal for G–C interaction was marginally significant (*p*-value = 3.26E-02). This was probably due to the fact that the non-negligible G–C correlation (Fig. 6 and Supplementary Data 6) would inflate the signal of G–C interaction in M1, 2 and 7 (all based on univariate framework). As shown in the simulations, the MRNM was the most reliable model (Supplementary Data 4 and 5). Hence, this demonstrates that conclusions from models using the MRNM applied to real data can differ from those obtained using methods based on more simplified models (Fig. 6 and Supplementary Data 6).

**Fig. 7:**
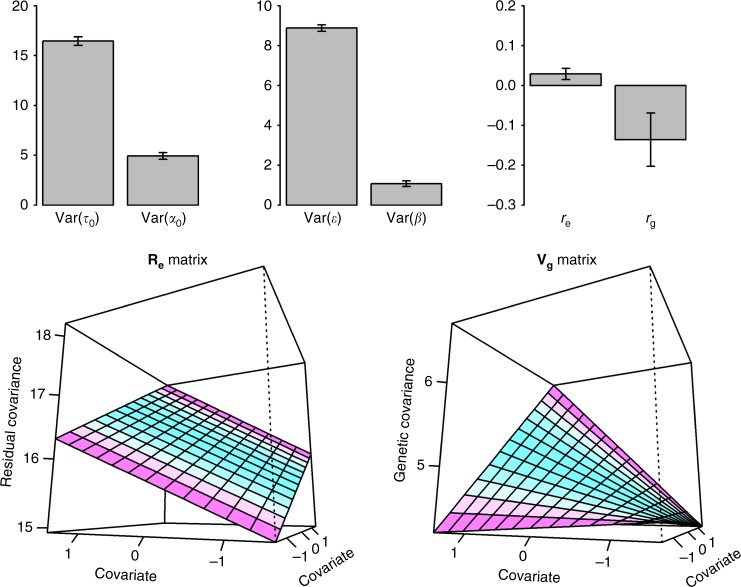
Estimated variance components and correlations from MRNM for BMI-NEU analysis. Var(**τ**_0_): Estimated residual variance for BMI as the main outcome. Var(**α**_0_): Estimated genetic variance for BMI as the main outcome. Var(**ε**): Estimated residual variance for NEU as the covariate. Var(**β**): Estimated genetic variance for NEU as the covariate. r_e_: Estimated residual correlation between BMI and NEU. r_g_: Estimated genetic correlation between BMI and NEU. Error bars are 95% confidence interval. **R**_**e**_ matrix is the residual (co)variance structure between different covariate levels (see Eq. 4), which is derived based on the estimated random regression coefficients and polynomial matrix as **R**_**e**_ = **ΦM**_**y**_**Φ**′. **Φ** is the matrix of polynomials evaluated at given covariate values, where entries of the first column are all **1**s and the second column is the standardised covariates of respective individuals. **M**_**y**_ is the variance–covariance matrix of random regression coefficients estimated from MRNM as $${\mathbf{M}}_{\mathbf{y}} = \left[ {\begin{array}{*{20}{c}} {{\mathrm{var}}({\mathbf{\tau }}_0)} & {{\mathrm{cov}}({\mathbf{\tau }}_0,{\mathbf{\tau }}_1)} \\ {{\mathrm{cov}}({\mathbf{\tau }}_0,{\mathbf{\tau }}_1)} & {{\mathrm{var}}({\mathbf{\tau }}_1)} \end{array}} \right] = \left[ {\begin{array}{*{20}{c}} {16.45({\mathrm{SE}}0.21)} & {0.54({\mathrm{SE}}0.11)} \\ {0.54({\mathrm{SE}}0.11)} & {0.06({\mathrm{SE}}0.18)} \end{array}} \right]$$. **V**_**g**_ matrix in is the genetic (co)variance structure between different covariate levels (see Eq. 2), which is derived based on the estimated random regression coefficients and polynomial matrix as **V**_**g**_ = **ΦK**_**y**_**Φ**′. **Φ** is the matrix of polynomials evaluated at given covariate values, where entries of the first column are all **1**s and the second column is the standardised covariates of respective individuals. **K**_**y**_ is the variance–covariance matrix of random regression coefficients estimated from MRNM as $${\mathbf{K}}_{\mathbf{y}}=\left[ {\begin{array}{*{20}{c}} {{\mathrm{var}}({\mathbf{\alpha }}_0)} & {{\mathrm{cov}}({\mathbf{\alpha }}_0,{\mathbf{\alpha }}_1)} \\ {{\mathrm{cov}}({\mathbf{\alpha }}_0,{\mathbf{\alpha }}_1)} & {{\mathrm{var}}({\mathbf{\alpha }}_1)} \end{array}} \right] = \left[ {\begin{array}{*{20}{c}} {4.94({\mathrm{SE}}0.17)} & {0.38({\mathrm{SE}}0.11)} \\ {0.38({\mathrm{SE}}0.11)} & {0.28({\mathrm{SE}}0.13)} \end{array}} \right]$$

We also analysed BMI using NEU^25,26^ as the covariate in the various models, observing evidence for interaction with RR-GREML (*p*-value = 6.82E-04) but not GCI-GREML(M1 and M2 in Table 1). We found strong G–C and R–C interactions when using either RNM (M3-M5) or MRNM (M8-M10). Evidence for interaction remained when the G–C interaction effects were adjusted for R–C interaction effects (*p*-value = 3.77E-04 for M7 and 1.08E-03 for M12) or vice versa (7.73E-06 for M6 and 2.36E-05 for M11). This shows that G–C and R–C interactions are both important and contribute to the shared aetiology between BMI and NEU. As shown in Fig. 7, both genetic and residual effects on BMI are significantly modulated by individual NEU while there also is a strong genetic association between them. It was noted that in contrast to BMI-SMK analysis, the results between RNM and MRNM were similar, possibly reflecting different shared genetic and environmental architecture between BMI and NEU, compared to BMI and SMK. The estimated genetic architecture from BMI-NEU analyses is depicted in Fig. 7, Supplementary Data 6 and Supplementary Fig. 13.

Lastly, we used PC1 as the covariate in the same analyses (Table 1) and as expected, found no significant interaction effects. Compared to SMK or NEU, the R–C interaction was dramatically less (Table 1 and Supplementary Data 6), probably because PC1 was calculated from genotype data for which the residual component was relatively small. We also found no evidence of G–C interaction, which was probably due to the fact that the sample was so homogeneous such that there was little power to detect interaction effects modulated by population difference.

The phenotypes of BMI showed some deviation from a normal distribution (kurtosis = 5.65 and skewness = 1.08). We explicitly tested the normality assumption using simulations, and found that rank-based inverse normal transformed (RINT) phenotypes could remedy type I error inflation due to non-normality (Supplementary Note 2). However, the conclusions drawn from our analyses were the same when either raw or RINT phenotypes are used (Table 1 vs. and Supplementary Table 8). Therefore, we report significances and estimations based on raw phenotypes here.

### Inflated residual variance using GREML with real data

**Fig. 8:**
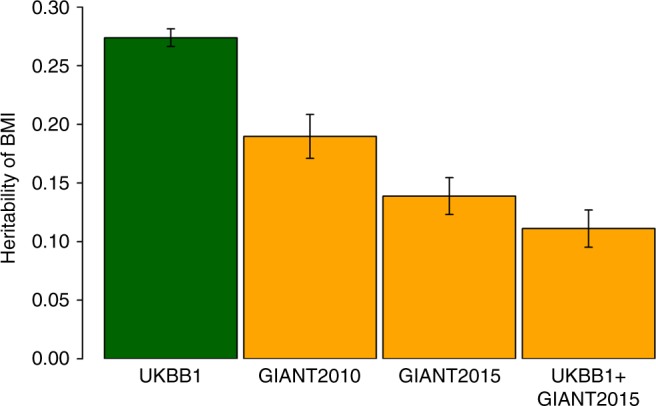
Estimated SNP-based heritability of BMI decreases with increasing numbers of cohorts. The UKBB1 estimate was reported by Ge et al.^23^, which used GWAS summary statistics based on the samples from the first release of UK Biobank. The GIANT2010 and GIANT 2015 estimates were reported by Duncan et al.^29^, which used GWAS summary statistics based on the GIANT consortium samples from ~80 to 125 cohorts, respectively. The UKBB1 + GIANT2015 estimate was reported by Ni et al.^30^. Bars are 95% confidence interval

We observed in simulation data that residual variances for a trait estimated from LDSC or GREML were inflated when there were G–C or R–C interactions (Fig. 5 and Supplementary Table 7), and this led to underestimates of SNP heritability. Hence, with real data, we tested the differences in the estimates of residual variances for BMI estimated from GREML and RNM (Table 2). For this real data analysis, we could not assess LDSC performance because it did not provide the standard error of estimated residual variance that was required for testing a statistical difference (Supplementary Note 3). For SMK and NEU that had significant interaction effects, the estimated residual variances from GREML were significantly higher than those from RNM (1.89% difference with *p*-value = 5.99E-04 and 2.04% difference with *p*-value = 7.12E-03, using a Wald test) (Table 2). As expected, there was no significant difference between the models when PC1 was considered as the covariate, because it had no interaction effects. We also fitted both SMK and NEU simultaneously and found that the difference between estimated residual variances from GREML and RNM was increased (3.28% with *p*-value 1.57E-04 using a Wald test) (Table 2). The estimated variance components for the interaction effects from the joint model (Supplementary Table 9) and the separate models (M4 in Supplementary Data 6) did not differ. We also observed that the estimated genetic variance varied little between using GREML or RNM (Fig. 5 and M3 in Supplementary Data 1 and 2), hence biased residual variance directly caused biased SNP-based heritability estimates. Inflated residual variance therefore underestimated SNP-based heritability, as also observed from an extensive meta-analysis across diverse study-cohorts^23,29,30^ that possibly increased the heterogeneity of covariates shared by the study-samples, hence increased the variance due to G–C and/or R–C interactions (Fig. 8). When comparing MRNM and MVGREML, the results did not differ much (Supplementary Table 10) although there were additional parameters such as cov(**α**_1_, **β**) and cov(**τ**_1_, **ε**) that were not explicitly parameterised in GREML. We did not fit multiple covariates jointly in MRNM because of our focus on SNP-based heritability comparisons based on univariate models (i.e., GREML vs. RNM) and due to the need to control computational demands.

**Table 2:** The difference between residual variances of BMI estimated from RNM^a^ and GREML^b^

	Difference^c^	SE^d^	Difference in %	SE of Difference in %	h^2^ (GREML)	h^2^ (RNM)	P^e^
SMK	−0.316	0.092	1.887	0.549	0.221	0.224	5.99E-04
NEU	−0.336	0.125	2.044	0.760	0.227	0.231	7.12E-03
PC1	0.016	0.088	−0.095	0.523	0.227	0.227	8.56E-01
SMK-NEU^f^	−0.588	0.156	3.279	0.870	0.227	0.233	1.57E-04

^a^Alternative model (H1) of M3 in Table 1

^b^Null model (H0) of M3 in Table 1

^c^Difference = the residual variance estimated from RNM − the residual variance estimated from univariate GREML

^d^Standard error of the difference was calculated based on the theory in Supplementary Note 3

^e^*P-*value was obtained based a two-tailed Wald test using the difference of residual variances and its SE

^f^The model jointly fitted both SMK and NEU as multiple covariates (see Methods)

### Meta-analysis approach and validation using UK Biobank data

For very large data sets, our proposed approach may become computationally infeasible (see Supplementary Table 11 for computational requirements). A solution could be to divide the data in various subsets and undertake a meta-analysis. We show that a meta-analysis^31^ of GCCI and RCCI results across different data subsets is useful and reliable (Supplementary Note 4).

## Discussion

Complex traits are determined by both genetic and environmental effects. Some environmental covariates of complex traits may themselves be determined by genetic and non-genetic factors. Genotype–covariate correlation and interactions (GCCI) and residual–covariate correlation and interaction effects (RCCI) may be important underlying factors shaping complex trait phenotypes^32^, yet not many studies have conducted analyses to detect these effects jointly in one model because of a lack of proper analysis models. In this study, we propose a flexible (multivariate) RNM to estimate genotype–covariate correlation and interactions and residual–covariate correlation and interaction effects for complex traits, which is powerful and reliable. The key findings are summarised as follows:

1. For continuous covariates, the proposed MRNM is a more appropriate model, compared to RR-GREML and GCI-GREML.
2. Covariates can be regulated by genetic and environmental factors that are possibly shared with the main response (GCCI and RCCI effects), which is the most plausible mechanism for many complex traits. It is desirable to model GCCI and RCCI effects appropriately (using multivariate RNM).
3. SNP-based heritability estimates from standard additive models should be carefully interpreted or revisited if covariate information is available.
4. The proposed models can be applied to large-scale biobank data by meta-analysis of results from sub-samples, for which the analyses are computationally feasible.

The existing methods for estimation of G–C interactions, i.e., RR-GREML and GCI-GREML, require that the outcome of interest (e.g., BMI) should be stratified into multiple discrete groups. The discrete grouping ignores the difference of covariate values for the individuals within each group, and results in some loss of information. In contrast, RNM and MRNM use a flexible model to fit a continuous covariate. Based on the analysis method that we believe to be the most appropriate for the data (MRNM) the G–C interaction estimate was much reduced and only borderline significant while R–C interaction was much more significant.

In the presence of G–C or R–C interactions, estimated SNP-based heritability of the main response variable by GREML or LDSC could be biased. The biased estimates reflect that the interaction effects are absorbed by residual variance and the overall estimated residual variance was inflated. The residual variance estimated from GREML was significantly higher than that from RNM for the BMI-SMK, BMI-NEU or BMI-SMK/NEU analysis using the real data. Currently reported SNP-based heritabilites estimated based on meta analysis of GWAS summary statistics from diverse study-cohorts tend to be lower when the number of study-cohorts is larger (as a proxy of heterogeneity) (Fig. 8), which can be partly explained by not properly modelling G–C and R–C interactions. This observation has an important implication because estimates from such meta-analyses (using LDSC) should be carefully interpreted when known key covariates were not included in the GWAS analysis model that generated the input for the LDSC analysis.

In this study, we found a strong negative R–C correlation and weak positive G–C correlation between BMI and smoking, which may support the phenomenon observed in several studies that heavier smokers tend to have lower BMI. The R–C interaction was shown to be highly significant. This suggests that the information about R–C interaction component is crucial such that that the main phenotypes (BMI) can be possibly controlled by changing the covariate (SMK), provided that the covariate is modifiable. In this example, the implication is that the intervention of increasing smoking could be used to control BMI^33^. While in this example the advice may not be practical for other health reasons, the principle can be used to other traits and diseases with modifiable covariates. The information from the G–C correlation and interaction can be useful for an early intervention (e.g., genomic medicine) although the magnitude of the effects is relatively small, compared to R–C components. We also investigated NEU and found strong G–C and R–C interactions, indicating the personality trait NEU is a major covariate influencing the environmental factor for BMI as well as revealing a novel genetic architecture of BMI to interact with different levels of NEU (Fig. 7). Both genetic and residual variances of BMI are significantly modulated by NEU, as well as there is significant genetic correlation between BMI and NEU. We included analyses using PC1 as the covariate in the model. Because of little variance among the homogenous sample, there were no significant interactions (Table 1 and Supplementary Table 8). In other circumstances, for example when using diverse samples from the population or even across different ethnic populations, then analyses that fit PC1 as a covariate might generate significant interaction estimates.

Our analytical framework could be extended to consider genotype-by-genotype interactions, i.e., epistasis, such that an interaction between the genetic component of a trait and a covariate might be partitioned as interaction variances in the proposed model. However, for the sorts of phenotypes considered here there is no power to disentangle epistasis from other interactions with the data available to us. However, the proposed approach may have important utility in the context of gene-expression, transcriptome and methylation data as novel covariates, for which the specific genetic architecture may be powered to detect epistatic interactions and correlations, and may merit future study. It is possible to use models that fit multiple covariates simultaneously as we did for fitting both SMK and NEU jointly (Table 2), which increase the proportion of the total phenotypic variance explained by the interaction components. Genomic partitioning analyses to describe GCCI and RCCI effects across the genome will be also useful to shed light on the latent genetic architecture of complex traits and diseases, which is possible by using the proposed approaches in this study.

An alternative approach to disentangle interaction from association is through the classical structural equation models^11^ applied to twin- or pedigree-based data. However, availability of such data is limited, restricting our ability to study GCCI effects for a wide range of complex traits and covariates. For example, phenotypes moderated by ancestry components (e.g., ethnic composition in humans or breed composition in animals) cannot be studied by an approach that is based on twins or relatives. It is also difficult to disentangle the genetic and shared environmental effects when using a pedigree-based approach. Standard REML packages (e.g., ASReml^34^) can be used to test the GCCI effects although it is questionable that the classical REML algorithm, which has been optimised for pedigree-based studies, can be computationally tractable when fitting genetic covariance structures based on genomic information^17^. Therefore, it may be infeasible investigate the GCCI effects using the classical REML packages although they have been applied widely in livestock^35,36^ and ecological genetics^13,37,38^ to explore the phenotype-genotype relationship across environmental gradients. When extending analyses to cover large-scale data such as the UK Biobank^24^, it is essential to develop computationally efficient methods that also correctly capture the GCCI effects, based on genomic information.

There are a number of limitations and caveats in this study. First, we only considered interaction of order *k* *=* 1 for both G–C and R–C interactions. Further study is required to validate performance with higher order interactions to generalise the proposed approach. Second, our approaches are flexible, but computationally demanding. For a large data set or when conducting randomisation tests, e.g., permutation or bootstrapping, it may not be computationally feasible to conduct an analysis within a reasonable time. Subsampling and meta-analysis approaches can reduce analysis time, however, the power is notably reduced, compared to when using the whole data (Supplementary Figs. 14 and 15). Third, the proposed methods do not estimate the direction of causality that can be determined by existing methods, e.g., Mendelian randomisation. Fourth, in application to real data we do not take account of ascertainment biases that may generate interactions and correlations in the sample of data which means that our results may not be representative of the populations from which the samples are drawn^39,40^. Lastly, when there are assumption violations, such as non-normality, correlation between random effects or wrong estimation models due to missing or misspecified information, one needs to carefully check model performance (as in the Supplementary Notes 2 and 5). For non-normally distributed traits, we recommend using RINT phenotypes to check the robustness of estimated interaction effects. Interaction effects will require careful interpretation, particularly for R–C effects which can be caused by residual heteroscedasticity by covariates^41^.

In conclusion, we showed that the multivariate RNM is able to effectively disentangle interaction from correlation and to generate unbiased estimates for G–C and R–C components. The concept of GCCI and RCCI is more plausible in explaining the genetic architecture of complex traits associated/interacted with covariates, which will shift the paradigm from a univariate to multivariate framework and from linear to non-linear models in complex trait analyses.

## Methods

UK Biobank’s scientific protocol and operational procedures were reviewed and approved by the North West Multi-centre Research Ethics Committee (MREC), National Information Governance Board for Health & Social Care (NIGB), and Community Health Index Advisory Group (CHIAG). The protocol of Atherosclerosis Risk in Communities Study (ARIC) study has been reviewed and approved by the Institutional Review Boards (IRB) of each participating institution, including the IRBs of the University of Minnesota, Johns Hopkins University, University of North Carolina, University of Mississippi Medical Center, and Wake Forest University. Research Ethics approval was obtained from University of South Australia Human Research Ethics Committee (HREC).

### Reaction norm model (RNM)

We only consider interaction of order *k* = 1 in the following equations. More general equations with a higher order can be found in Supplementary Note 1.

To account for phenotypic plasticity and norms of reaction in response to different covariate or environmental conditions among samples^35,36^, the dependent variable for individual *i* can be modelled as

1 $$y_i = b_i + g_i + e_i = b_i + \alpha _{i0} + \alpha _{i1} \cdot c_i + e_i$$

where *y*_*i*_ is the phenotypic observation, *b*_*i*_ represents fixed effects, *g*_*i*_ is the random genetic effect, *α*_*i0*_ and *α*_*i1*_ are the zero and first order of random regression coefficients, *c*_i_ is the covariate value, and *e*_*i*_ is the residual effect for the *i*th individual. Assuming that each individual has unique covariate value, the variance–covariance matrix of observed phenotypes (*y*_i_) is

$${\mathrm{var}}({\mathbf{y}}) = \left[ {\begin{array}{*{20}{c}} {{\mathbf{Z}}_1{\mathbf{A}}{\mathrm{\sigma }}_{g_1}^2{\mathbf{Z}}_1^\prime + {\mathbf{Z}}_1{\mathbf{I}}{\mathrm{\sigma }}_{e_1}^2{\mathbf{Z}}_1^\prime } & \cdots & {{\mathbf{Z}}_1{\mathbf{A}}\sigma _{g_{1,N}}{\mathbf{Z}}_N^\prime + {\mathbf{Z}}_1{\mathbf{I}}\sigma _{e_{1,N}}{\mathbf{Z}}_N^\prime } \\ \vdots & \ddots & \vdots \\ {{\mathbf{Z}}_{\mathrm{N}}{\mathbf{A}}\sigma _{g_{1,N}}{\mathbf{Z}}_1^\prime + {\mathbf{Z}}_{\mathrm{N}}{\mathbf{I}}\sigma _{e_{1,N}}{\mathbf{Z}}_1^\prime } & \cdots & {{\mathbf{Z}}_N{\mathbf{A}}{\mathrm{\sigma }}_{g_N}^2{\mathbf{Z}}_{\mathrm{N}}^\prime + {\mathbf{Z}}_N{\mathbf{I}}{\mathrm{\sigma }}_{e_N}^2{\mathbf{Z}}_N^\prime } \end{array}} \right],$$

where **A** is the *N* × *N* genomic relationship matrix based on genome-wide SNP information, **Z**_i_ is an incidence matrix for *g*_*i*_, and **I** is an *N* × *N* identity matrix. The terms ${\mathrm{\sigma }}_{g_i}^2$ and ${\mathrm{\sigma }}_{e_i}^2$ denote the genetic and residual variances at the covariate level for individual *i*. The terms $\sigma _{g_{i,j}}$and $\sigma _{e_{i,j}}$ indicate the genetic and residual covariance between the covariate levels for indiv*i*dual *i* and *j* (*i* = 1, …, *N*, and *j* = 1, …, *N*), respectively^17^. The random genetic and residual effect are assumed following a normal distribution with mean as zero and variance as ${\mathbf{A}}\sigma _g^2$ and ${\mathbf{I}}\sigma _e^2$. The random genetic effect, *g*_*i*_, can be regressed on the covariate gradient (reaction norm), which can be efficiently modelled with random regression coefficients. The variance–covariance matrix of random regression coefficients (**K**) is

$${\mathbf{K}} = {\mathrm{cov}}\left( {{\boldsymbol{\alpha }}_0,{\boldsymbol{\alpha }}_1} \right) = \left[ {\begin{array}{*{20}{c}} {{\mathrm{var}}({\boldsymbol{\alpha }}_0)} & {{\mathrm{cov}}({\boldsymbol{\alpha }}_0,{\boldsymbol{\alpha }}_1)} \\ {{\mathrm{cov}}({\boldsymbol{\alpha }}_0,{\boldsymbol{\alpha }}_1)} & {{\mathrm{var}}({\boldsymbol{\alpha }}_1)} \end{array}} \right]$$

where **α**_0_ and **α**_1_ are the zero and first order random regression coefficients. The genetic (co)variance matrix of genetic effects between *N* individuals or *N* covariate values (because each individual has unique covariate value) is a function of random regression coefficients and polynomials, which can be expressed as

$${\mathbf{V}}_{\mathbf{g}} = {\mathbf{\Phi K}} {\mathbf{\Phi }}\prime = \left[ {\begin{array}{*{20}{c}} {{\mathrm{\sigma }}_{g_1}^2} & \cdots & {\sigma _{g_{1,N}}} \\ \vdots & \ddots & \vdots \\ {\sigma _{g_{N,1}}} & \cdots & {{\mathrm{\sigma }}_{g_N}^2} \end{array}} \right]$$

where **Φ** is the *N* × 2 matrix of the zero and first order polynomials of *N* covariate values, that is ${{\Phi }}_{{i}} = [{{c}}_i^0,{{c}}_i^1]$.

Given that this model does not explicitly parameterise the correlation between *y*_*i*_ and *c*_*i*_, it naively assumes that *y*_*i*_ and *c*_*i*_ are uncorrelated. For this reason, this model is also referred to as a genotype–covariate interaction (G–C interaction) model.

### Multivariate reaction norm model (MRNM)

The naïve assumption of the univariate RNM (or G–C interaction model) that *y*_*i*_ and *c*_i_ are uncorrelated is often violated. In a more proper model, the covariate value for individual *i* is decomposed as *c*_*i*_ = *μ*_*i*_ + *β*_*i*_ + *ε*_*i*_, where *μ*_*i*_ is fixed effects, *β*_*i*_ is the random genetic effect, and *ε*_i_ is the residual effect. When considering the main response (**y**) and covariate (**c**) jointly in a multivariate model, the variance–covariance matrix of observed phenotypes *y*_*i*_ and *c*_*i*_ is

$${\mathrm{cov}}\left( {{\mathbf{y}},{\mathbf{c}}} \right) = \left[ {\begin{array}{*{20}{c}} {{\mathbf{Z}}_1{\mathbf{A}}{\mathrm{\sigma }}_{g_1}^2{\mathbf{Z}}_1^\prime + {\mathbf{Z}}_1{\mathbf{I}}{\mathrm{\sigma }}_{e_1}^2{\mathbf{Z}}_1^\prime } & \cdots & {{\mathbf{Z}}_1{\mathbf{A}}\sigma _{g_{1,N}}{\mathbf{Z}}_N^\prime + {\mathbf{Z}}_1{\mathbf{I}}\sigma _{e_{1,N}}{\mathbf{Z}}_N^\prime } & {{\mathbf{Z}}_1{\mathbf{A}}\sigma _{g_1,\beta }{\mathbf{Z}}_c^\prime + {\mathbf{Z}}_1{\mathbf{I}}\sigma _{e_1,\varepsilon}{\mathbf{Z}}_c^\prime } \\ \vdots & \ddots & \vdots & \vdots \\ {{\mathbf{Z}}_{\mathrm{N}}{\mathbf{A}}\sigma _{g_{1,N}}{\mathbf{Z}}_1^\prime + {\mathbf{Z}}_{\mathrm{N}}{\mathbf{I}}\sigma _{e_{1,N}}{\mathbf{Z}}_1^\prime } & \cdots & {{\mathbf{Z}}_N{\mathbf{A}}{\mathrm{\sigma }}_{g_N}^2{\mathbf{Z}}_{\mathrm{N}}^\prime + {\mathbf{Z}}_N{\mathbf{I}}{\mathrm{\sigma }}_{e_N}^2{\mathbf{Z}}_N^\prime } & {{\mathbf{Z}}_N{\mathbf{A}}\sigma _{g_N,\beta }{\mathbf{Z}}_c^\prime + {\mathbf{Z}}_N{\mathbf{I}}\sigma _{e_N,\varepsilon }{\mathbf{Z}}_c^\prime } \\ {{\mathbf{Z}}_c{\mathbf{A}}\sigma _{g_1,\beta }{\mathbf{Z}}_1^\prime + {\mathbf{Z}}_{\mathrm{c}}{\mathbf{I}}\sigma _{e_1,\varepsilon }{\mathbf{Z}}_1^\prime } & \cdots & {{\mathbf{Z}}_c{\mathbf{A}}\sigma _{g_N,\beta }{\mathbf{Z}}_N^\prime + {\mathbf{Z}}_{\mathrm{c}}{\mathbf{I}}\sigma _{e_N,\varepsilon }{\mathbf{Z}}_N^\prime } & {{\mathbf{Z}}_c{\mathbf{A}}{\mathrm{\sigma }}_\beta ^2{\mathbf{Z}}_c^\prime + {\mathbf{Z}}_c{\mathbf{I}}{\mathrm{\sigma }}_\varepsilon ^2{\mathbf{Z}}_c^\prime } \end{array}} \right]$$

where **Z**_*c*_ is an incidence matrix for the vector of the random genetic and residual effects, **β** and **ε**, underlying **c**. The genetic and residual variances of covariate **c** are denoted as ${\mathrm{\sigma }}_\beta ^2$ and ${\mathrm{\sigma }}_\varepsilon ^2$, respectively. The terms $\sigma _{g_i,\beta }$ and $\sigma _{e_i,\varepsilon }$ indicate the genetic and residual covariance between main trait and covariate at the *i*th covariate levels (*i* *=* 1, …, *N*), respectively. The random genetic and residual effects of y are the same as defined above. The random genetic and residual effect of **c** are assumed following a normal distribution with mean as zero and variance as ${\mathbf{A}}\sigma _\beta ^2$ and ${\mathbf{I}}\sigma _\varepsilon ^2$. The genetic (co)variance matrix of individual genetic effects in the multivariate model can be written as

2 $${\mathbf{V}}_{{\mathrm{g}},{\mathrm{\beta }}} = \left[ {\begin{array}{*{20}{c}} {{\mathbf{\Phi K}}_{\mathbf{y}}{\mathbf{\Phi }}\prime } & {{\mathbf{\Phi K}}_{{\mathbf{y}},{\mathbf{c}}}} \\ {{\mathbf{K}}_{{\mathbf{y}},{\mathbf{c}}}^\prime {\mathbf{\Phi }}\prime } & {{\mathrm{var}}({\mathbf{\beta }})} \end{array}} \right] = \left[ {\begin{array}{*{20}{c}} {{\mathrm{\sigma }}_{g_1}^2} & \cdots & {\sigma _{g_{1,N}}} & {\sigma _{g_1,\beta }} \\ \vdots & \ddots & \vdots & \vdots \\ {\sigma _{g_{N,1}}} & \cdots & {{\mathrm{\sigma }}_{g_N}^2} & {\sigma _{g_N,\beta }} \\ {\sigma _{g_1,\beta }} & \cdots & {\sigma _{g_N,\beta }} & {{\mathrm{\sigma }}_\beta ^2} \end{array}} \right]$$

where **K**_**y**_ is the same as **K** defined above, and **K**_**y,c**_ consists of the covariance between **β** and the random regression coefficients, that is

$${\mathbf{K}}_{{\mathbf{y}},{\mathbf{c}}} = \left[ {\begin{array}{*{20}{c}} {\begin{array}{*{20}{c}} {{\mathrm{cov}}({\boldsymbol{\alpha }}_0,{\boldsymbol{\beta }})} \\ {{\mathrm{cov}}({\boldsymbol{\alpha }}_1,{\boldsymbol{\beta }})} \end{array}} \end{array}} \right].$$

The multivariate residual covariance structure is

$${\mathbf{R}}_{{\mathbf{e}},{\boldsymbol{\varepsilon }}} = \left[ {\begin{array}{*{20}{c}} {{\mathrm{var}}({\mathbf{e}})} & {{\mathrm{cov}}({\mathbf{e}},{\boldsymbol{\varepsilon }})} \\ {{\mathrm{cov}}({\mathbf{e}},{\boldsymbol{\varepsilon }})} & {{\mathrm{var}}({\boldsymbol{\varepsilon }})} \end{array}} \right],$$

where **e** is the vector of residual effects for the main phenotypes, assuming that var(**e**) is homogenous across different levels of covariate values, i.e., ${\mathrm{\sigma }}_{e_1}^2 = {\mathrm{\sigma }}_{e_2}^2 = , \ldots , = {\mathrm{\sigma }}_{e_N}^2$, which can be relaxed for the case of heterogeneous residual variances (see the next section), and **ε** is the vector of residual effects for the covariate, defined as above, and var(**ε**) is the residual variance of the covariate.

This model explicitly parameterises covariance between the random regression coefficients for the main phenotypes and the genetic effects underlying the covariate (i.e., **K**_**y,c**_), therefore, is referred to as a genotype–covariate correlation and interaction (GCCI) model. Importantly, values for cov(**α**_0_, **β**) or cov(**e**, **ε**) are often non-negligible. Neglecting these terms can cause confounding between G–C correlation and interaction, thereby generating spurious signals and biased estimates for the interaction. Yet many studies do not account for G–C correlations when estimating and testing G–C interaction^16^.

### MRNM accounting for heterogeneous residual variance (RCCI)

The models we described so far assume that the residual variance for the main phenotypes, var(**e**), is homogeneous across different values of the covariate. However, it is often possible that residual–covariate (R–C) correlation and interaction exist, resulting in heterogeneous residual variances across different covariate values. To account for this possibility, MRNM can be further generalised as

3 $$y_i = b_i + g_i + e_i = b_i + \alpha _{i0} + \alpha _{i1} \cdot c_i + \tau _{i0} + \tau _{i1} \cdot c_i$$

where the residual term, *e*_*i*_, can be also regressed on the covariate gradient, modelled with the zero and first order of random regression coefficients (*τ*_*i*0_ and *τ*_*i*1_) and polynomial of the covariate.

The variance–covariance structure of the genetic effect for this model is the same as for the multivariate reaction norm model described in Eq. (2) in the previous section. The multivariate residual covariance structure in this generalised MRNM becomes

4 $${\mathbf{R}}_{{\mathbf{e}},{\boldsymbol{\varepsilon }}} = \left[ {\begin{array}{*{20}{c}} {{\mathbf{\Phi M}}_{\mathbf{y}}{\mathbf{\Phi }}\prime } & {{\mathbf{\Phi M}}_{{\mathbf{y}},{\mathbf{c}}}} \\ {{\mathbf{M}}_{{\mathbf{y}},{\mathbf{c}}}^\prime {\mathbf{\Phi }}\prime } & {{\mathrm{var}}({\boldsymbol{\varepsilon }})} \end{array}} \right] = \left[ {\begin{array}{*{20}{c}} {{\mathrm{\sigma }}_{e_1}^2} & \cdots & {\sigma _{e_{1,N}}} & {\sigma _{e_1,\varepsilon }} \\ \vdots & \ddots & \vdots & \vdots \\ {\sigma _{e_{N,1}}} & \cdots & {{\mathrm{\sigma }}_{e_N}^2} & {\sigma _{e_N,\varepsilon }} \\ {\sigma _{e_1,\varepsilon }} & \cdots & {\sigma _{e_N,\varepsilon }} & {{\mathrm{\sigma }}_\varepsilon ^2} \end{array}} \right]$$

where **M**_**y**_ is the variance and covariance matrix of random regression coefficients for the residual components and can be written as

$${\mathbf{M}}_{\mathbf{y}} = {\mathrm{cov}}\left( {{\boldsymbol{\tau }}_0,{\boldsymbol{\tau }}_1} \right) = \left[ {\begin{array}{*{20}{c}} {{\mathrm{var}}\left( {{\boldsymbol{\tau }}_0} \right)} & {{\mathrm{cov}}\left( {{\boldsymbol{\tau }}_0,{\boldsymbol{\tau }}_1} \right)} \\ {{\mathrm{cov}}\left( {{\boldsymbol{\tau }}_0,{\boldsymbol{\tau }}_1} \right)} & {{\mathrm{var}}\left( {{\boldsymbol{\tau }}_1} \right)} \end{array}} \right],$$

where **τ**_0_ and **τ**_1_ are the zero and first order random regression coefficients for the residual effects. **M**_**y**,**c**_ is a vector with the covariance between **ε** and the random regression coefficients for the residual effects, and can be expressed as

$${\mathbf{M}}_{{\mathbf{y}},{\mathbf{c}}} = \left[ {\begin{array}{*{20}{c}} {{\mathrm{cov}}({\boldsymbol{\tau }}_0,{\boldsymbol{\varepsilon }})} \\ {{\mathrm{cov}}\left( {{\boldsymbol{\tau }}_1,{\mathrm{\varepsilon }}} \right)} \end{array}} \right].$$

### RNM with multiple covariates

RNM can be further extended to include multiple covariates. A model fitting with multiple covariates can be expressed as

$$y_i = b_i + \mathop {\sum}\limits_{j = 1}^x {g_{ij}} + e_i = b_i + \mathop {\sum}\limits_{j = 1}^x {\left( {\alpha _{ij0} + \alpha _{ij1} \cdot c_{ij}} \right) + e_i} ,$$

where* x* is the number of random effects, each of which is associated with a unique combination of a relationship matrix and covariate (see below), *α*_*ij*0_ and *α*_*ij*1_ are the zero and first order random regression coefficient for the *j*th random effect and *c*_*ij*_ is the covariate value for the *j*th random effect. Therefore, this model is a multiple random effects model fitting multiple components^22^, but it allows the inclusion of interaction effects for each random effect. As in the original multiple random effects model, it is assumed that there is no correlation between the random effects^42^.

The variance–covariance matrix of observed phenotypes (*y*_*i*_) for this multiple random effects model is

$${\mathrm{var}}\left( {\mathbf{y}} \right) = \left[ {\begin{array}{*{20}{c}} {\mathop {\sum}\limits_{j = 1}^x {{\mathbf{Z}}_1} {\mathbf{A}}_j{\mathrm{\sigma }}_{(g_1)_j}^2{\mathbf{Z}}_1^\prime + {\mathbf{Z}}_1{\mathbf{I}}{\mathrm{\sigma }}_{e_1}^2{\mathbf{Z}}_1^\prime } & \cdots & {\mathop {\sum}\limits_{j = 1}^x {{\mathbf{Z}}_1} {\mathbf{A}}_j\sigma _{(g_{1,N})_j}{\mathbf{Z}}_N^\prime + {\mathbf{Z}}_1{\mathbf{I}}\sigma _{e_{1,N}}{\mathbf{Z}}_N^\prime } \\ \vdots & \ddots & \vdots \\ {\mathop {\sum}\limits_{j = 1}^x {{\mathbf{Z}}_{\mathrm{N}}} {\mathbf{A}}_j\sigma _{(g_{1,N})_j}{\mathbf{Z}}_1^\prime + {\mathbf{Z}}_{\mathrm{N}}{\mathbf{I}}\sigma _{e_{1,N}}{\mathbf{Z}}_1^\prime } & \cdots & {\mathop {\sum}\limits_{j = 1}^x {{\mathbf{Z}}_N} {\mathbf{A}}_j{\mathrm{\sigma }}_{(g_N)_j}^2{\mathbf{Z}}_{\mathrm{N}}^\prime + {\mathbf{Z}}_N{\mathbf{I}}{\mathrm{\sigma }}_{e_N}^2{\mathbf{Z}}_N^\prime } \end{array}} \right],$$

where **A**_j_ is the genomic relationship matrix for the *j*th random effect, ${\mathrm{\sigma }}_{(g_i)_j}^2$ is the genetic variance at the *i*th covariate level for the *j*th random effect, $\sigma _{(g_{1,N})_j}$ is, for example, the genetic covariance between the first and the last covariate levels, and other terms are defined as above. As in the RNM fitting with a single covariate, *g*_*ij*_ in each random effect (*j* = 1 ~ *x*) can be regressed on the covariate gradient in the same manner. The variance–covariance matrix of random regression coefficients for each random effect (**K**_*j*_) can be written as

$${\mathbf{K}}_j = {\mathrm{cov}}\left( {{\boldsymbol{\alpha }}_{j0},{\boldsymbol{\alpha }}_{j1}} \right) = \left[ {\begin{array}{*{20}{c}} {{\mathrm{var}}({\boldsymbol{\alpha }}_{j0})} & {{\mathrm{cov}}({\boldsymbol{\alpha }}_{j0},{\boldsymbol{\alpha }}_{j1})} \\ {{\mathrm{cov}}({\boldsymbol{\alpha }}_{j0},{\boldsymbol{\alpha }}_{j1})} & {{\mathrm{var}}({\boldsymbol{\alpha }}_{j1})} \end{array}} \right].$$

Similarly, the genetic (co)variance matrix of individual genetic effects between *N* individuals can be obtained as

$${\mathbf{V}}_{{\mathrm{g}}_j} = {\mathbf{\Phi }}_j{\mathbf{K}}_j{\mathbf{\Phi }}_j^\prime = \left[ {\begin{array}{*{20}{c}} {{\mathrm{\sigma }}_{(g_1)_j}^2} & \cdots & {\sigma _{(g_{1,N})_j}} \\ \vdots & \ddots & \vdots \\ {\sigma _{(g_{1,N})_j}} & \cdots & {{\mathrm{\sigma }}_{(g_N)_j}^2} \end{array}} \right],$$

where **Φ**_*j*_ is the *N* × 2 matrix of covariate polynomials for the *j*th random effect, and the variance–covariance components were defined as above. This multiple random effects model fitting with multiple covariates can be feasibly extended to MRNM with GCCI and RCCI although the number of parameters increases exponentially.

All models described above can be fitted using MTG2^17^.

### Simulated data

Phenotypic simulation was based on individual genotypes from the GWAS data of the Atherosclerosis Risk in Communities Study (ARIC) cohort. We used autosomes only and applied the standard quality control (QC) to genotypes, which included MAF > 0.01, SNP call rate > 0.95, sample call rate > 0.95 and Hardy–Weinberg Equilibrium *p*-value > 0.001, keeping qualified genotyped SNPs. After the standard QC, 583,058 SNPs and 8,291 individuals remained. In addition, we estimated pair-wise relatedness from the remaining SNPs and randomly excluded one individual from each pair with an estimated relatedness greater than 0.05. This relatedness cut-off QC reduced the sample to 7263 individuals.

*Simulation under GCCI model (MRNM G–C model)*: We simulated phenotypes for the main response (**y**) and covariate (**c**) under the GCCI model with the first order interaction effect, i.e., *k* = 1. In the simulation, we used the following covariance structure for the **K**_**y**_ matrix in Eq. (2) as

$${\mathbf{K}}_{\mathbf{y}} = \left[ {\begin{array}{*{20}{c}} {{\mathrm{var}}({\boldsymbol{\alpha }}_0)} & {{\mathrm{cov}}({\boldsymbol{\alpha }}_0,{\boldsymbol{\alpha }}_1)} \\ {{\mathrm{cov}}({\boldsymbol{\alpha }}_0,{\boldsymbol{\alpha }}_1)} & {{\mathrm{var}}({\boldsymbol{\alpha }}_1)} \end{array}} \right] = \left[ {\begin{array}{*{20}{c}} 1 & {0.05} \\ {0.05} & {{\mathrm{var}}({\boldsymbol{\alpha }}_1)} \end{array}} \right].$$

We used a wide range of the G–C interaction with var(**α**_1_) set at 0, 0.25, 0.5, 0.75 or 1. For the covariate, *c*_*i*_ = *μ*_*i*_ + *β*_*i*_ + *ε*_*i*_, var(**β**) and var(**ε**) were set at 1.

The values for the **K**_**y,c**_ matrix (Eq. 2) were used in the simulation as

$${\mathbf{K}}_{{\mathbf{y}},{\mathbf{c}}} = \left[ {\begin{array}{*{20}{c}} {\begin{array}{*{20}{c}} {{\mathrm{cov}}({\boldsymbol{\alpha }}_0,{\mathbf{\beta }})} \\ {{\mathrm{cov}}({\boldsymbol{\alpha }}_1,{\mathbf{\beta }})} \end{array}} \end{array}} \right] = \left[ {\begin{array}{*{20}{c}} {0.5} \\ 0 \end{array}} \right],$$

and

$${\mathbf{R}}_{{\mathbf{e}},{\boldsymbol{\varepsilon }}} = \left[ {\begin{array}{*{20}{c}} {\begin{array}{*{20}{c}} {{\mathrm{var}}({\mathbf{e}})} & {{\mathrm{cov}}({\mathbf{e}},{\boldsymbol{\varepsilon }})} \\ {{\mathrm{cov}}({\mathbf{e}},{\boldsymbol{\varepsilon }})} & {{\mathrm{var}}({\boldsymbol{\varepsilon }})} \end{array}} \end{array}} \right] = \left[ {\begin{array}{*{20}{c}} 1 & {0.3} \\ {03} & 1 \end{array}} \right].$$

*Simulation under RCCI model (MRNM R–C model)*: In this simulation, we used the following covariance structure for the **K**_y_ matrix in Eq. (2) as

$${\mathbf{K}}_{\mathbf{y}} = \left[ {\begin{array}{*{20}{c}} {{\mathrm{var}}({\boldsymbol{\alpha }}_0)} & {{\mathrm{cov}}({\boldsymbol{\alpha }}_0,{\boldsymbol{\alpha }}_1)} \\ {{\mathrm{cov}}({\boldsymbol{\alpha }}_0,{\boldsymbol{\alpha }}_1)} & {{\mathrm{var}}({\boldsymbol{\alpha }}_1)} \end{array}} \right] = \left[ {\begin{array}{*{20}{c}} 1 & 0 \\ 0 & 0 \end{array}} \right].$$

For the covariate, *c*_*i*_ = *μ*_*i*_ + *β*_*i*_ + *ε*_*i*_, var(**β**) and var(**ε**) were set at 1.

The values for the **K**_**y,c**_ matrix (Eq. 2) were used in the simulation as

$${\mathbf{K}}_{{\mathrm{y}},{\mathrm{c}}} = \left[ {\begin{array}{*{20}{c}} {\begin{array}{*{20}{c}} {{\mathrm{cov}}({\boldsymbol{\alpha }}_0,{\mathbf{\beta }})} \\ {{\mathrm{cov}}({\boldsymbol{\alpha }}_1,{\mathbf{\beta }})} \end{array}} \end{array}} \right] = \left[ {\begin{array}{*{20}{c}} {0.5} \\ 0 \end{array}} \right].$$

The **M**_**y**_ and **M**_**y**,**c**_ matrices (Eqs 3 and 4) were specified in the simulation as follows:

$${\mathbf{M}}_{\mathbf{y}} = \left[ {\begin{array}{*{20}{c}} {{\mathrm{var}}\left( {{\boldsymbol{\tau }}_0} \right)} & {{\mathrm{cov}}\left( {{\boldsymbol{\tau }}_0,{\boldsymbol{\tau }}_1} \right)} \\ {{\mathrm{cov}}\left( {{\boldsymbol{\tau }}_0,{\boldsymbol{\tau }}_1} \right)} & {{\mathrm{var}}\left( {{\boldsymbol{\tau }}_1} \right)} \end{array}} \right] = \left[ {\begin{array}{*{20}{c}} 1 & {0.05} \\ {0.05} & {{\mathrm{var}}\left( {{\boldsymbol{\tau }}_1} \right)} \end{array}} \right]\,{\mathrm{with}}\,{\mathrm{var}}({\boldsymbol{\tau}}_1) = 0,0.25\,{\mathrm{or}}\,1$$

and

$${\mathbf{M}}_{{\mathbf{y}},{\mathbf{c}}} = \left[ {\begin{array}{*{20}{c}} {{\mathrm{cov}}({\boldsymbol{\tau }}_0,{\boldsymbol{\varepsilon }})} \\ {{\mathrm{cov}}\left( {{\boldsymbol{\tau }}_1,{\boldsymbol{\varepsilon }}} \right)} \end{array}} \right] = \left[ {\begin{array}{*{20}{c}} {{\mathrm{cov}}({\boldsymbol{\tau }}_0,{\boldsymbol{\varepsilon }})} \\ 0 \end{array}} \right]\,{\mathrm{with}}\,{\mathrm{cov}}({\boldsymbol{\tau}}_0,{\mathbf{\varepsilon}}) = 0\,{\mathrm{or}}\,0.3.$$

*Simulation under GCCI and RCCI model (MRNM Full model)*: Similar to the GCCI simulations above, we used values for the **K**_**y**_ matrix in Eq. (2) as

$${\mathbf{K}}_{\mathbf{y}} = \left[ {\begin{array}{*{20}{c}} {{\mathrm{var}}({\boldsymbol{\alpha }}_0)} & {{\mathrm{cov}}({\boldsymbol{\alpha }}_0,{\boldsymbol{\alpha }}_1)} \\ {{\mathrm{cov}}({\boldsymbol{\alpha }}_0,{\boldsymbol{\alpha }}_1)} & {{\mathrm{var}}({\boldsymbol{\alpha }}_1)} \end{array}} \right] = \left[ {\begin{array}{*{20}{c}} 1 & {0.05} \\ {0.05} & {{\mathrm{var}}({\boldsymbol{\alpha }}_1)} \end{array}} \right].$$

We performed simulations with var(**α**_1_) set at 0, 0.25 or 1, and for the covariate, both var(**β**) and var(**ε**) were set at 1.

The **K**_**y,c**_, **M**_**y**_ and **M**_**y**,**c**_ matrices (Eqs. 3 and 4) were specified in the simulation as follows:

$${\mathbf{K}}_{{\mathbf{y}},{\mathbf{c}}} = \left[ {\begin{array}{*{20}{c}} {\begin{array}{*{20}{c}} {{\mathrm{cov}}({\boldsymbol{\alpha }}_0,{\mathbf{\beta }})} \\ {{\mathrm{cov}}({\boldsymbol{\alpha }}_1,{\mathbf{\beta }})} \end{array}} \end{array}} \right] = \left[ {\begin{array}{*{20}{c}} {{\mathrm{cov}}\left( {{\boldsymbol{\alpha }}_0,{\mathbf{\beta }}} \right)} \\ 0 \end{array}} \right]\,{\mathrm{with}}\,{\mathrm{cov}}({\boldsymbol{\alpha}}_0,{\mathbf{\beta}}) = 0\,{\mathrm{or}}\,0.5,$$

$${\mathbf{M}}_{\mathbf{y}} = \left[ {\begin{array}{*{20}{c}} {{\mathrm{var}}\left( {{\boldsymbol{\tau }}_0} \right)} & {{\mathrm{cov}}\left( {{\boldsymbol{\tau }}_0,{\boldsymbol{\tau }}_1} \right)} \\ {{\mathrm{cov}}\left( {{\boldsymbol{\tau }}_0,{\boldsymbol{\tau }}_1} \right)} & {{\mathrm{var}}\left( {{\boldsymbol{\tau }}_1} \right)} \end{array}} \right] = \left[ {\begin{array}{*{20}{c}} 1 & {0.05} \\ {0.05} & {{\mathrm{var}}\left( {{\boldsymbol{\tau }}_1} \right)} \end{array}} \right]\,{\mathrm{with}}\,{\mathrm{var}}({\boldsymbol{\tau}}_1) = 0.25\,{\mathrm{or}}\,1$$

and

$${\mathbf{M}}_{{\mathbf{y}},{\mathbf{c}}} = \left[ {\begin{array}{*{20}{c}} {{\mathrm{cov}}({\boldsymbol{\tau }}_0,{\boldsymbol{\varepsilon }})} \\ {{\mathrm{cov}}\left( {{\boldsymbol{\tau }}_1,{\boldsymbol{\varepsilon }}} \right)} \end{array}} \right] = \left[ {\begin{array}{*{20}{c}} {{\mathrm{cov}}({\boldsymbol{\tau }}_0,{\boldsymbol{\varepsilon }})} \\ 0 \end{array}} \right]\,{\mathrm{with}}\,{\mathrm{cov}}({\boldsymbol{\tau}}_0,{\mathbf{\varepsilon}}) = 0\,{\mathrm{or}}\,0.3.$$

According to the normal distribution assumption in the models, all genetic and residual values were drawn from normal distributions with mean zero and variance–covariance structures specified as above. In the presence or absence of interactions with simulated data under these various models, we assessed bias, type I error rate and power for LDSC^19,20^, GREML^18^, MVGREML^17^, RR-GREML^16^, GCI-GREML^18^, RNM, and MRNM. We used likelihood ratio tests to get *p*-values to detect interaction effects and also estimated variance components in the models. We used inverse normal *p*-values transformed from the raw *p*-values using qnorm function in R^43^ for a clearer comparison across the methods.

### Real data

*Data and Quality control*: We used the UK Biobank data^24^, which initially contained 488,377 individuals and 92,693,895 imputed SNPs across autosomes. Stringent quality control was applied to the genotype data at both individual and SNP levels. Specifically, we excluded individuals who met one of the following criteria: (1) does not have white British ancestry, (2) has a genotype missing rate > 0.05, (3) whose reported gender does not match with the gender inferred using genotype data, and (4) has a putative sex chromosome aneuploidy. At the SNP level, we excluded SNPs with an INFO score < 0.6, with a MAF < 0.01, with a Hardy–Weinberg equilibrium *p*-value < 1E-4, or with a call rate < 0.95. We excluded ambiguous or duplicated SNPs. We only used the HapMap 3 SNPs in the main analyses because they are reliable and robust to bias in estimating SNP-based heritability and genetic correlation^44–46^. In addition, we excluded individual population outliers with the first or second PC outside six standard deviations of the population mean. For individuals who were in the first and second releases of UK biobank genotype data, we calculated the discordance rate between imputed genotype of the two versions for each individual and for each SNP, and excluded individuals and SNPs with a discordance rate lager than 0.05. We also excluded one individual randomly from any pair with a genomic relationship larger than 0.05. After the QC above, 288,866 individuals and 1,130,918 SNPs remained. Of these remaining individuals 91,472 were from the first release of UK Biobank (denoted as UKBB1) and 66,281 individuals with complete records of covariates were used in the main analyses. The rest of 197,394 individuals were from the second release of UK Biobank (denoted as UKBB2) and 115,053 with complete records of covariates were used in the validation and meta-analyses.

*Main response variable and covariates*: We applied the novel (M)RNM model using BMI as the main response variable to estimate the GCCI/RCCI components with each of several covariates, including pack years of smoking (SMK), neuroticism score (NEU) or the first principal component (PC1) provided by the UK Biobank. We also fitted the model that includes multiple covariates (e.g., SMK and NEU) jointly, i.e., RNM with multiple covariates. For all analyses, covariates were standardised as mean zero and variance 1. Prior to model fitting, we adjusted the main response variable (BMI) for confounders including genotype batch, assessment centre at which participant consented, year of birth, sex, age, diet variation, diet change, the first 15 PCs, SMK, weekly alcohol consumption (ALC) and Townsend deprivation index at recruitment (TDI). In the analyses using NEU and PC1 as the covariates, we further adjusted BMI for NEU to correct the mean difference. The distribution of each covariate is in Supplementary Fig. 16.

When including the covariate (i.e., SMK, NEU, or PC1) as the second trait in a MRNM, it was also pre-adjusted for the confounders in a similar way as for the main trait (i.e., BMI). For instance, as the second trait in a MRNM, SMK was pre-adjusted for BMI, genotype batch, assessment centre at which participant consented, year of birth, sex, age, diet variation, diet change, the first 15 PCs, ALC, and TDI. NEU was pre-adjusted for BMI, genotype batch, assessment centre at which participant consented, year of birth, sex, age, diet variation, diet change, the first 15 PCs, ALC, TDI and SMK. PC1 was pre-adjusted for BMI, genotype batch, assessment centre at which participant consented, year of birth, sex, age, diet variation, diet change, the first 15 PCs except the first one (PC1), ALC, TDI, SMK and NEU.

Detailed information regarding covariates used in the interaction models is described below and that for other confounders used to adjust the main phenotypes is in Supplementary Note 6.

*Pack years of smoking (SMK)*: We combined pack years adult smoking as proportion of life span exposed to smoking (UK Biobank data field: 20162) and ever smoked (UK Biobank data field 20160) as SMK. The distribution of SMK is in Supplementary Fig. 16. For RR-GREML and GCI-GREML, following Robinson et al.^16^, we stratified SMK into four levels: 8,773 individuals with SMK > 0.8, 9,192 individuals with 0.5 ≤ SMK ≤ 0.8, 11,741 individuals with 0 < SMK < 0.5, and 36,575 individuals with SMK = 0 (i.e., never smoked).

*Neuroticism score (NEU)*: The neuroticism score (data field 20127) of a given individual was indexed by the number of ‘yes’s to 12 touchscreen questions that evaluate neurotic behaviours. The distribution of NEU is in Supplementary Fig. 16. For RR-GREML and GCI-GREML, we stratified the data into four groups according to NEU level: 20,901 individuals with NEU ≤ 2, 16,161 individuals with 2 < NEU ≤ 5, 10,895 individuals with 5 < NEU ≤ 8, and 6,417 individuals with 8 < NEU ≤ 12.

*The fist principal components of genotype (PC1)*: PCs were pre-calculated by the UK Biobank. Detailed information regarding the calculation is described elsewhere^47^. Briefly, PCs-loadings were estimated using fastPCA^48^ based on 407,219 unrelated individuals and 147,604 markers that were pruned to minimise linkage disequilibrium, onto where all samples were projected, to generate a set of PC scores. For RR-GREML and GCI-GREML, we stratified the sample into four groups based on quartiles of PC1.

*Meta analyses of real data*: The proposed MRNM requires individual-level genotype data, which makes it computationally demanding. As sample size increases (e.g., the second release of UK Biobank), the computing time increases substantially. To complete the analyses within a reasonable timeframe, we used a meta-analysis approach. We performed two sets of meta-analyses, one across two groups within UKBB1 to assess the performance of the meta-analysis, compared to that of the whole UKBB1 data analysis, and the other across UKBB1 and UKBB2.

Meta-analyses within UKBB1. We randomly divided the UKBB1 into two groups of equal size (denoted as g1 and g2), and fitted all models mentioned above for each group. *P*-values from each group were meta-analysed using the Fisher’s method^31^. We then compared these *p*-values with those based on the whole UKBB1 data set.

Meta-analyses across UKBB1 and UKBB2. In UKBB2, 197,394 individuals with genotype data passed the QC, of which 94 K have no missing covariates and main response. Similar to meta-analyses within UKBB1, we randomly divided the UKBB2 into two groups of equal size (denoted as G1 and G2), and fitted all models mentioned above for each group. We then meta-analysed the results from G1, G2, and UKBB1 (denoted as G0) using the Fisher’s method^31^. For UKBB2, the same pre-adjustment as for UKBB1 was applied to the main response and covariates as the second trait in MRNM.

### URLs

UK Biobank, http://www.ukbiobank.ac.uk/

LDSC, https://github.com/bulik/ldsc

GCTA, http://cnsgenomics.com/software/gcta/

Plink1.9, https://www.cog-genomics.org/plink2

MTG2, https://sites.google.com/site/honglee0707/mtg2

### Reporting summary

Further information on research design is available in the Nature Research Reporting Summary linked to this article.

## Supplementary information

Supplementary Information

Reporting Summary

Description of Additional Supplementary Files

Supplementary Data 1

Supplementary Data 2

Supplementary Data 3

Supplementary Data 4

Supplementary Data 5

Supplementary Data 6

Supplementary Data 7

Supplementary Data 8

Supplementary Data 9

Source Data

## Data Availability

The simulated data can be obtained from the authors on request. We also used the genotype data of ARIC study under accession code phs000090 in the database of Genotypes and Phenotypes. All other relevant data is available upon request. The source data underlying Figs. 1–8 and Supplementary Figs. 1–23 are provided as a Source Data file.

## Code availability

The RNM and MRNM for GCCI and RCCI analyses are fully implemented in MTG2 version 2.14.
