# Supplementary material for: Genotype–covariate correlation and interaction disentangled by a whole-genome multivariate reaction norm model
Source: Nat Commun. 2019 May 20;10:2239. doi: 10.1038/s41467-019-10128-w (PMC6527612; doi:10.1038/s41467-019-10128-w)
Supplement: Supplementary file 1 — Supplementary Information [file 41467_2019_10128_MOESM1_ESM.pdf]

Genotype-covariate correlation and interaction disentangled by a whole-genome multivariate reaction norm model

Ni et al.

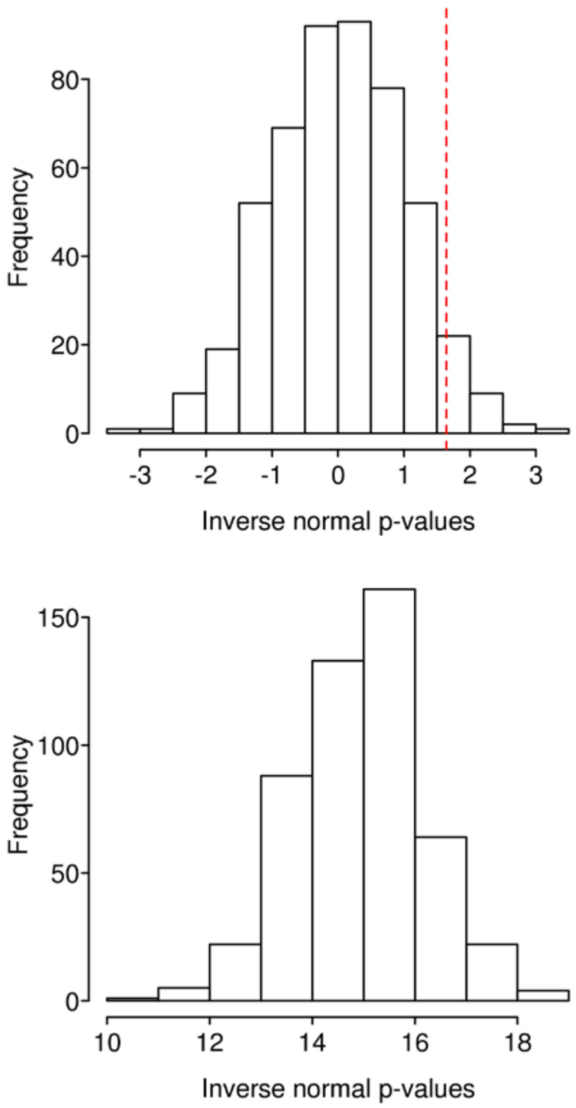

Supplementary Figure 1. Type I error (top) and power (bottom) for detecting G-C interaction using MRNM for the same data used in Figures 2 and 3.

The MRNM performed similarly with RNM (Figures 2 and 3), i.e. the type I error rate was 0.05 and the power was 1 in this figure.

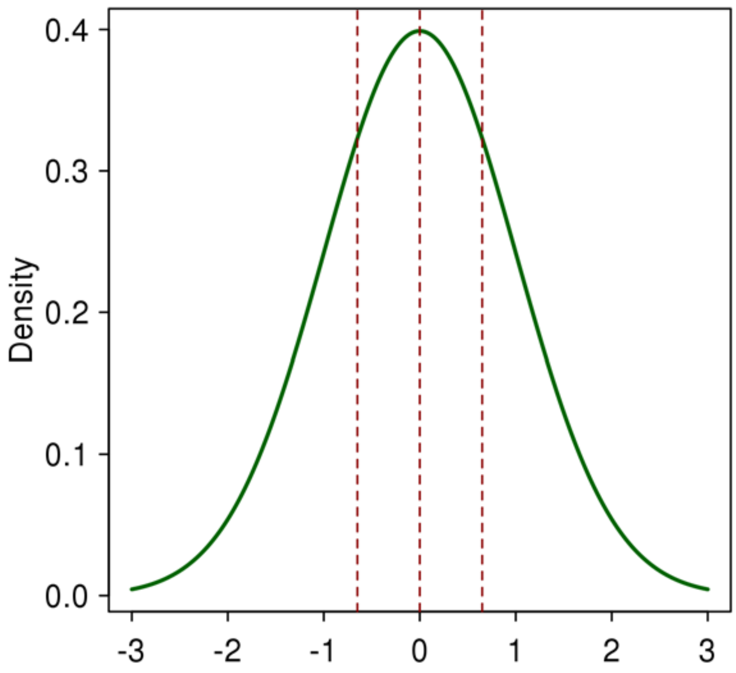

Supplementary Figure 2. Artificial heterogeneous density and variances across four discrete groups classified according to 25, 50 and 75% quantiles (red dashed lines) of a covariate that is continuous and normally distributed.

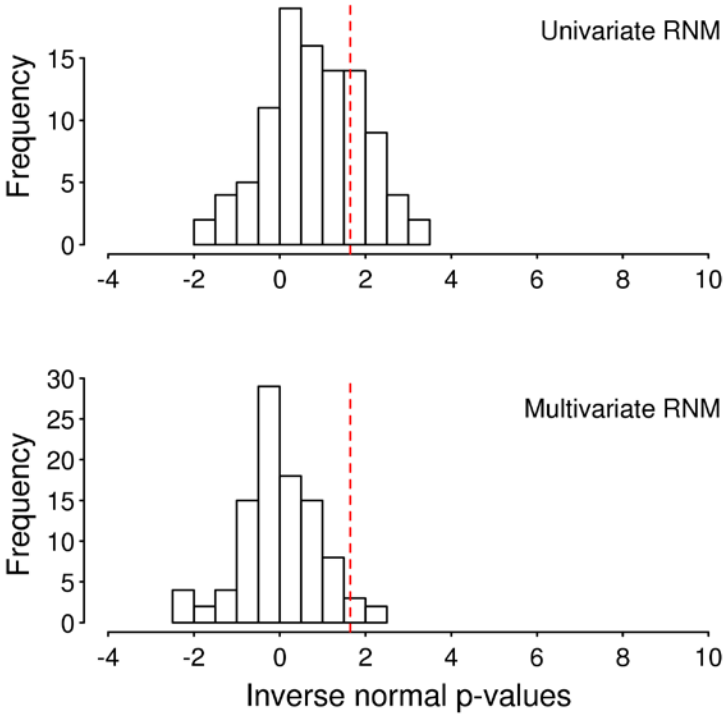

Supplementary Figure 3. Spurious signals generated by incorrect (univariate) model can be controlled by applying multivariate RNM for detecting G-C interaction in a simulation with relatively low genetic correlation.

One hundred replicates of data were simulated under a null model that assumed genotype-covariate correlation but no genotype-covariate interaction. Simulation was based QCed ARIC data consisting of 7,263 individuals and 583,058 SNPs. The model is specified as $\mathbf{y} = \boldsymbol{\alpha}_0 + \boldsymbol{\alpha}_1 \times \mathbf{c} + \mathbf{e}$ with $\mathbf{c} = \boldsymbol{\beta} + \boldsymbol{\varepsilon}$, all effects drawn from a multivariate normal distribution, where the variance-covariance structure between $\boldsymbol{\alpha}_0$, $\boldsymbol{\beta}$, and $\boldsymbol{\alpha}_1$ (in this order) is $\begin{bmatrix} 1 & 0.1 & 0 \\ 0.1 & 1 & 0 \\ 0 & 0 & 0 \end{bmatrix}$ and that between $\mathbf{e}$ and $\boldsymbol{\varepsilon}$ is $\begin{bmatrix} 1 & 0.3 \\ 0.3 & 1 \end{bmatrix}$. For every replicate, a univariate RNM and a multivariate RNM were fitted separately to obtain a p-value for the G-C interaction by comparing the null (H_0) and alternative hypothesis (H_1) model. For the univariate RNM, the H_0 and H_1 models were $\mathbf{y} = \boldsymbol{\alpha}_0 + \mathbf{e}$ and $\mathbf{y} =$

$\boldsymbol{\alpha_0} + \boldsymbol{\alpha_1}\times\mathbf{c} + \mathbf{e}$. For the multivariate RNM, the H_0 and H_1 models were $\mathbf{y} = \boldsymbol{\alpha_0} + \mathbf{e}$ with $\mathbf{c} = \boldsymbol{\beta} + \boldsymbol{\varepsilon}$ and $\mathbf{y} = \boldsymbol{\alpha_0} + \boldsymbol{\alpha_1}\times\mathbf{c} + \mathbf{e}$ with $\mathbf{c} = \boldsymbol{\beta} + \boldsymbol{\varepsilon}$. This figure shows the proportions of significant p-values, i.e., type I error rate, for both models, which are 0.25 (univariate RNM) and 0.04 (multivariate RNM). Note that p-values are inverse normal transformed, such that the statistical significance level, i.e., 1.65, shown as dashed lines, is equivalent to the 0.05 level before the transformation. Refer Supplementary Table 3 for the estimated variance components.

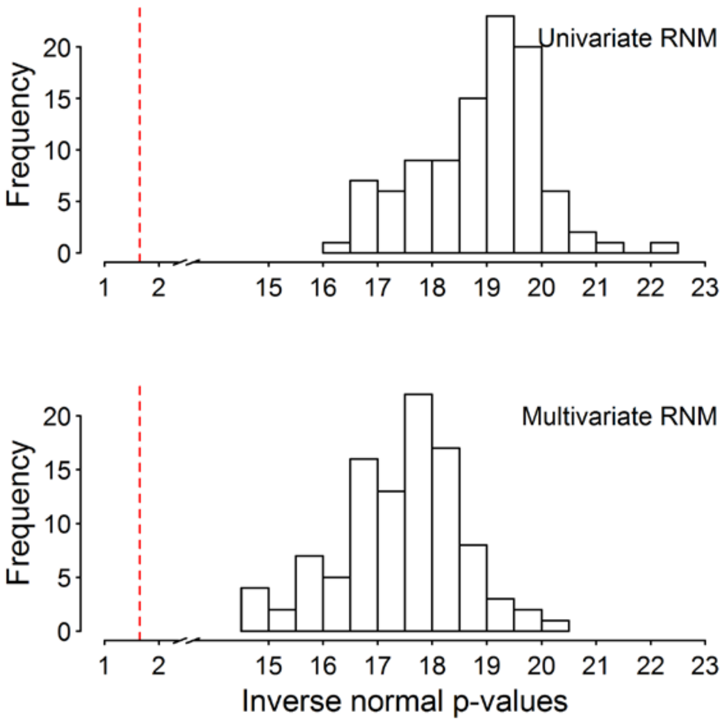

Supplementary Figure 4. Univariate RNM and multivariate RNM have a similar level of statistical power for detecting G-C interaction.

A hundred replicates of data were simulated under a model that assumed the presence of genotype–covariate correlation and interaction. Simulation was based QCed ARIC data consisting 7,263 individuals and 583,058 SNPs. The model is specified as $\mathbf{y} = \boldsymbol{\alpha_0} + \boldsymbol{\alpha_1} \times \mathbf{c} + \mathbf{e}$ with $\mathbf{c} = \boldsymbol{\beta} + \boldsymbol{\varepsilon}$, all effects drawn from a multivariate normal distribution, where the variance-covariance structure of $\boldsymbol{\alpha_0}$, $\boldsymbol{\beta}$, and $\boldsymbol{\alpha_1}$ (in this order) is $\begin{bmatrix} 1 & 0.5 & 0.05 \\ 0.5 & 1 & 0 \\ 0.05 & 0 & 0.25 \end{bmatrix}$ and that of $\mathbf{e}$ and $\boldsymbol{\varepsilon}$ is $\begin{bmatrix} 1 & 0.3 \\ 0.3 & 1 \end{bmatrix}$. For every replicate, a univariate RNM and a multivariate RNM were fitted separately to obtain a p-value for the G-C interaction by comparing the null (H_0) and alternative hypothesis (H_1) model. For the univariate RNM, the H_0 and H_1 models were $\mathbf{y} = \boldsymbol{\alpha_0} + \mathbf{e}$ and $\mathbf{y} = \boldsymbol{\alpha_0} + \boldsymbol{\alpha_1} \times \mathbf{c} +$

$\mathbf{e}$. For the multivariate RNM, the H_0 and H_1 models were $\mathbf{y} = \boldsymbol{\alpha_0} + \mathbf{e}$ with $\mathbf{c} = \boldsymbol{\beta} + \boldsymbol{\varepsilon}$ and $\mathbf{y} = \boldsymbol{\alpha_0} + \boldsymbol{\alpha_1}\times\mathbf{c} + \mathbf{e}$ with $\mathbf{c} = \boldsymbol{\beta} + \boldsymbol{\varepsilon}$. This figure shows the proportions of significant p-values, i.e., statistical power, for the two models, which are 1 for both. Note that p-values are inverse normal transformed, such that the statistical significance level, i.e., 1.65, shown as dashed lines, is equivalent to the 0.05 level before the transformation.

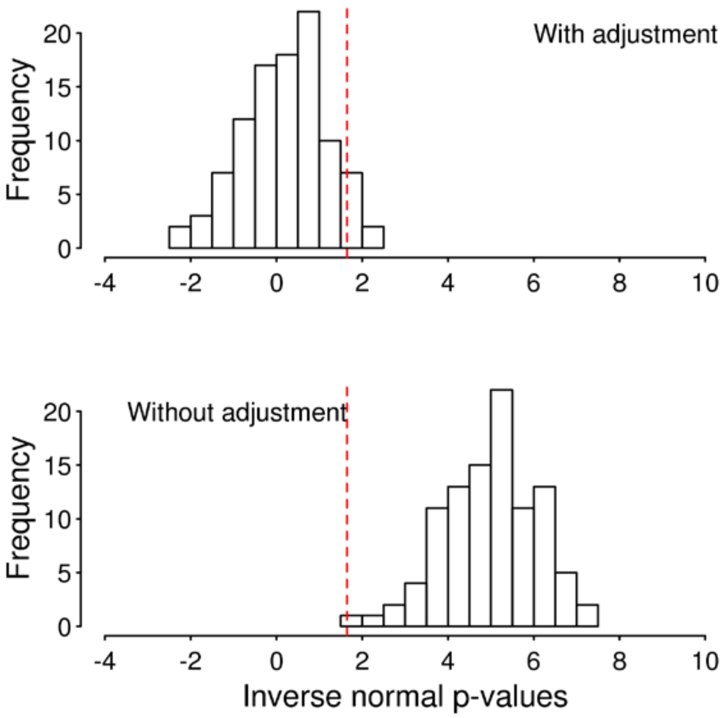

Supplementary Figure 5. Adjusting the main trait for the covariate can control spurious signals of G-C interaction when using RNM.

One hundred replicates of data were simulated under a null model that assumed genotype-covariate correlation but no genotype-covariate interaction. Simulation was based QCed ARIC data consisting of 7,263 individuals and 583,058 SNPs. The model is specified as $\mathbf{y} = \boldsymbol{\alpha}_0 + \boldsymbol{\alpha}_1 \times \mathbf{c} + \mathbf{e}$ with $\mathbf{c} = \boldsymbol{\beta} + \boldsymbol{\varepsilon}$, all effects drawn from a multivariate normal distribution, where the variance-covariance structure between $\boldsymbol{\alpha}_0$, $\boldsymbol{\beta}$, and $\boldsymbol{\alpha}_1$ (in this order) is $\begin{bmatrix} 1 & 0.5 & 0 \\ 0.5 & 1 & 0 \\ 0 & 0 & 0 \end{bmatrix}$ and that between $\mathbf{e}$ and $\boldsymbol{\varepsilon}$ is $\begin{bmatrix} 1 & 0.3 \\ 0.3 & 1 \end{bmatrix}$. For each replicate, a RNM was fitted under two scenarios, one where the main trait (i.e., y) was adjusted for the covariate (i.e., c) using a linear regression, and the other where the main trait was not adjusted. For each replicate, a p-value was obtained via a likelihood ratio test comparing the null (H_0) and alternative hypothesis (H_1) models. The H_0 and H_1 models

were $\mathbf{y} = \boldsymbol{\alpha}_0 + \mathbf{e}$ and $\mathbf{y} = \boldsymbol{\alpha}_0 + \boldsymbol{\alpha}_1 \times \mathbf{c} + \mathbf{e}$. In the top panel, the phenotypes (**y**) were adjusted for the covariate (**c**) as a fixed effect in a linear model and the residuals were used. In the bottom panel, the phenotypes were used without such adjustment. This figure shows the proportions of statistically significant p-values for detecting a genotype-covariate interaction, i.e., type I error rate, under the two scenarios, which are 0.04 (top) and 1 (bottom). Note that p-values are inverse normal transformed, such that the statistical significance level, i.e., 1.65, shown as dashed lines, is equivalent to the 0.05 level before the transformation.

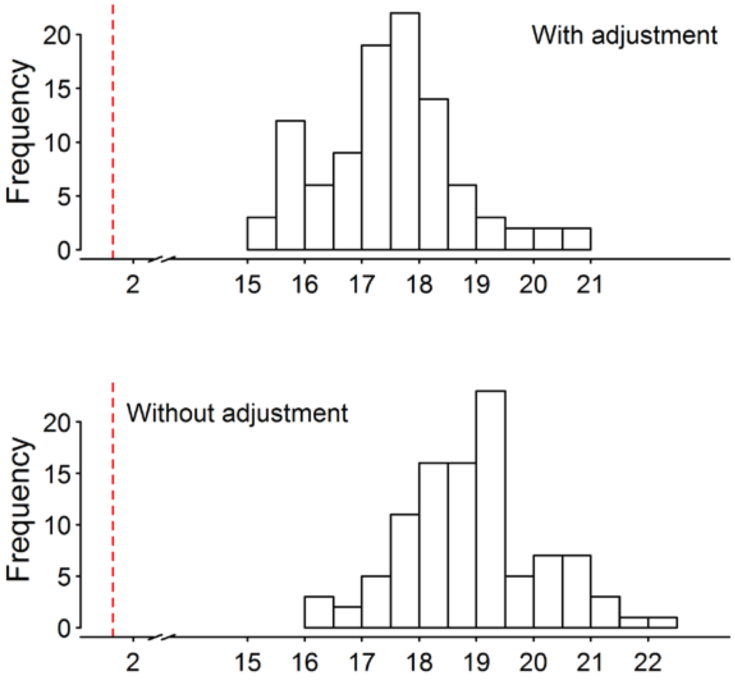

Supplementary Figure 6. The statistical power of detecting G-C interaction with or without adjusting the main trait for the covariate when using RNM.

One hundred replicates of data were simulated under a model that assumed the presence of genotype-covariate correlation and interaction. Simulation was based QCed ARIC data consisting of 7,263 individuals and 583,058 SNPs. The model is specified as $\mathbf{y} = \boldsymbol{\alpha_0} + \boldsymbol{\alpha_1} \times \mathbf{c} + \mathbf{e}$ with $\mathbf{c} = \boldsymbol{\beta} + \boldsymbol{\varepsilon}$, all effects drawn from a multivariate normal distribution, where the variance-covariance structure of $\boldsymbol{\alpha_0}$, $\boldsymbol{\beta}$, and $\boldsymbol{\alpha_1}$ (in this order) is $\begin{bmatrix} 1 & 0.5 & 0.05 \\ 0.5 & 1 & 0 \\ 0.05 & 0 & 0.25 \end{bmatrix}$ and that of $\mathbf{e}$ and ε is $\begin{bmatrix} 1 & 0.3 \\ 0.3 & 1 \end{bmatrix}$. For each replicate, a RNM was fitted under two scenarios, one where the main trait (i.e., y) was adjusted for the covariate (i.e., c) using a linear regression, and the other where the main trait was not adjusted. For each replicate, a p-value was obtained via a likelihood ratio test comparing the null (H_0) and alternative hypothesis (H_1) models. The H_0 and H_1 models were $\mathbf{y}$ =

$\boldsymbol{\alpha_0} + \mathbf{e}$ and $\mathbf{y} = \boldsymbol{\alpha_0} + \boldsymbol{\alpha_1}\times\mathbf{c} + \mathbf{e}$. In the top panel, the phenotypes (**y**) were adjusted for the covariate (**c**) as a fixed effect in a linear model and the residuals were used. In the bottom panel, the phenotypes were used without such adjustment. This figure shows the proportions of statistically significant p-values for detecting the genotype-covariate interaction, i.e., statistical power, under the two scenarios, which is 1 for both. Note that p-values are inverse normal transformed, such that the statistical significance level, i.e., 1.65, shown as dashed lines, is equivalent to the 0.05 level before the transformation.

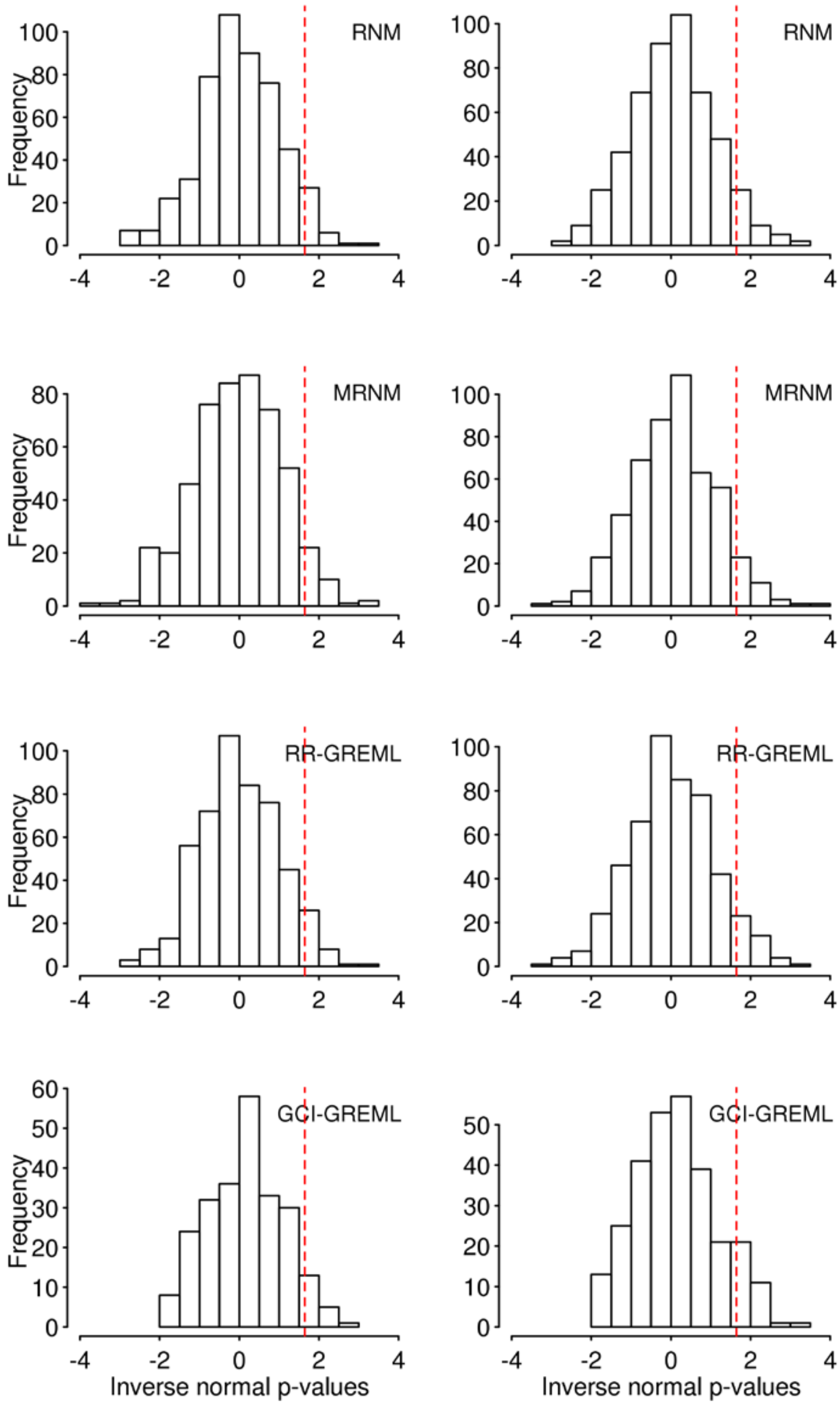

Supplementary Figure 7. Type I error rate of detecting genotype-covariate interaction is under control for RNM, MRNM, RR-GREML and GCI-GREML.

Five hundred replicates of data were simulated under each of two scenarios that assumed no genotype-covariate interaction but a residual-covariate interaction of different magnitudes. Simulation was based QCed ARIC data consisting of 7,263 individuals and 583,058 SNPs. The models of the two scenarios are specified as $\mathbf{y} = \boldsymbol{\alpha_0} + \boldsymbol{\tau_0} + \boldsymbol{\tau_1}\times\mathbf{c}$ with $\mathbf{c} = \boldsymbol{\beta} + \boldsymbol{\varepsilon}$, all effects drawn from a multivariate normal distribution, where the variance-covariance structure between $\boldsymbol{\alpha_0}$ and $\boldsymbol{\beta}$ is $\begin{bmatrix} 1 & 0 \\ 0 & 1 \end{bmatrix}$ and that between $\boldsymbol{\tau_0}$, $\boldsymbol{\varepsilon}$ and $\boldsymbol{\tau_1}$ is $\begin{bmatrix} 1 & 0 & 0.05 \\ 0 & 1 & 0 \\ 0.05 & 0 & \mathrm{var}(\boldsymbol{\tau_1}) \end{bmatrix}$ with var($\boldsymbol{\tau_1}$) = 0.25 (left panels) or 1 (right panels). For every replicate, each of four methods, i.e., RNM, MRNM, RR-GREML and GCI-GREML, was applied to obtain a p-value for detecting a G-C interaction via a comparison between the null (H_0) and alternative hypothesis (H_1) models. For RNM, the H_0 and H_1 models were $\mathbf{y} = \boldsymbol{\alpha_0} + \boldsymbol{\tau_0} + \boldsymbol{\tau_1}\times\mathbf{c}$ and $\mathbf{y} = \boldsymbol{\alpha_0} + \boldsymbol{\alpha_1}\times\mathbf{c} + \boldsymbol{\tau_0} + \boldsymbol{\tau_1}\times\mathbf{c}$. For MRNM, the H_0 and H_1 models were $\mathbf{y} = \boldsymbol{\alpha_0} + \boldsymbol{\tau_0} + \boldsymbol{\tau_1}\times\mathbf{c}$ with $\mathbf{c} = \boldsymbol{\beta} + \boldsymbol{\varepsilon}$ and $\mathbf{y} = \boldsymbol{\alpha_0} + \boldsymbol{\alpha_1}\times\mathbf{c} + \boldsymbol{\tau_0} + \boldsymbol{\tau_1}\times\mathbf{c}$ with $\mathbf{c} = \boldsymbol{\beta} + \boldsymbol{\varepsilon}$. For RR-GREML and GCI-GREML, the H_0 and H_1 models were $\mathbf{y} = \boldsymbol{\alpha_0} + \mathbf{e}$ and $\mathbf{y} = \boldsymbol{\alpha_0} + \boldsymbol{\alpha_1}\times\mathbf{c} + \mathbf{e}$. In RR-GREML and GCI-GREML, samples were arbitrarily stratified into four different groups according to the covariate levels. RR-GREML explicitly estimates residual variance for each of the four groups whereas GCI-GREML assumes homogeneous residual variance across the four groups and estimates a single residual variance. This figure shows the proportions of significant p-values, i.e., type I error rate, for each method (top to bottom) under the two simulation scenarios (left versus right). For var($\boldsymbol{\tau_1}$) = 0.25 (left panels), type I error rates from top to bottom are 0.052, 0.04, 0.058 and 0.024, respectively. For var($\boldsymbol{\tau_1}$) = 1 (right panels), type I error rates from top to bottom are 0.06, 0.06, 0.062 and 0.058, respectively. Note that p-values are inverse normal transformed, such that the statistical significance level, i.e., 1.65, shown as dashed lines,

is equivalent to the 0.05 level before the transformation. Refer to Supplementary Data 1 for estimated variance components of the four methods.

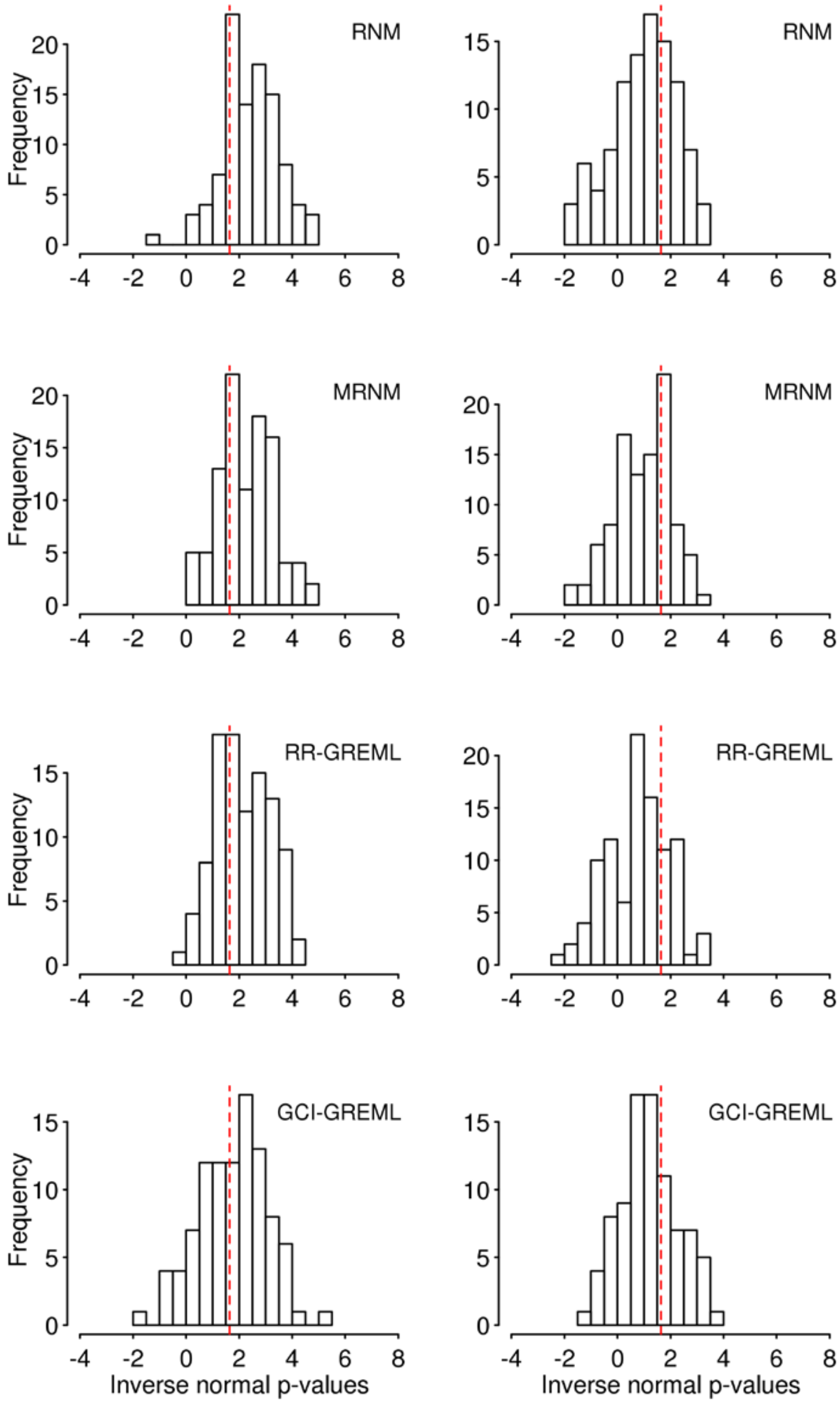

Supplementary Figure 8. Statistical power of detecting genotype-covariate interaction for RNM, MRNM, RR-GREML and GCI-GREML.

One hundred replicates of data were simulated under a model that assumed the presence of genotype-covariate interaction and residual-covariate interaction. Simulation was based QCed ARIC data consisting of 7,263 individuals and 583,058 SNPs. The model are specified as $\mathbf{y} = \boldsymbol{\alpha}_0 + \boldsymbol{\alpha}_1 \times \mathbf{c} + \boldsymbol{\tau}_0 + \boldsymbol{\tau}_1 \times \mathbf{c}$ with $\mathbf{c} = \boldsymbol{\beta} + \boldsymbol{\varepsilon}$, all effects drawn from a multivariate normal distribution, where the variance-covariance structure of $\boldsymbol{\alpha}_0$, $\boldsymbol{\beta}$, and $\boldsymbol{\alpha}_1$ (in this order) is $\begin{bmatrix} 1 & 0 & 0.05 \\ 0 & 1 & 0 \\ 0.05 & 0 & 0.25 \end{bmatrix}$ and that of $\boldsymbol{\tau}_0$, $\boldsymbol{\varepsilon}$, and $\boldsymbol{\tau}_1$ is $\begin{bmatrix} 1 & 0 & 0.05 \\ 0 & 1 & 0 \\ 0.05 & 0 & \mathrm{var}(\boldsymbol{\tau}_1) \end{bmatrix}$ with var($\boldsymbol{\tau}_1$) =0.25 for one scenario and 1 for the other. For every replicate, each of four methods, i.e., RNM, MRNM, RR-GREML and GCI-GREML, was applied separately to obtain a p-value for detecting a G-C interaction via a comparison between the null (H_0) and alternative hypothesis (H_1) models. For RNM, the H_0 and H_1 models were $\mathbf{y} = \boldsymbol{\alpha}_0 + \boldsymbol{\tau}_0 + \boldsymbol{\tau}_1 \times \mathbf{c}$ and $\mathbf{y} = \boldsymbol{\alpha}_0 + \boldsymbol{\alpha}_1 \times \mathbf{c} + \boldsymbol{\tau}_0 + \boldsymbol{\tau}_1 \times \mathbf{c}$. For MRNM, the H_0 and H_1 models were $\mathbf{y} = \boldsymbol{\alpha}_0 + \boldsymbol{\tau}_0 + \boldsymbol{\tau}_1 \times \mathbf{c}$ with $\mathbf{c} = \boldsymbol{\beta} + \boldsymbol{\varepsilon}$ and $\mathbf{y} = \boldsymbol{\alpha}_0 + \boldsymbol{\alpha}_1 \times \mathbf{c} + \boldsymbol{\tau}_0 + \boldsymbol{\tau}_1 \times \mathbf{c}$ with $\mathbf{c} = \boldsymbol{\beta} + \boldsymbol{\varepsilon}$. For RR-GREML and GCI-GREML, the H_0 and H_1 models were $\mathbf{y} = \boldsymbol{\alpha}_0 + \mathbf{e}$ and $\mathbf{y} = \boldsymbol{\alpha}_0 + \boldsymbol{\alpha}_1 \times \mathbf{c} + \mathbf{e}$. In RR-GREML and GCI-GREML, samples were arbitrarily stratified into four different groups according to the covariate levels. RR-GREML explicitly estimates residual variance for each of the four groups whereas GCI-GREML assumes homogeneous residual variance across the four groups and estimates a single residual variance. This figure shows the proportions of significant p-values, i.e., power, for each method (top to bottom) under the two simulation scenarios (left versus right). For var($\boldsymbol{\tau}_1$) = 0.25 (left panel), power from top to bottom are 0.77, 0.69, 0.62 and 0.54, respectively. For var($\boldsymbol{\tau}_1$) = 1 (right panel), power from top to bottom are 0.35, 0.31, 0.23 and 0.28, respectively. Note that p-values are inverse normal transformed, such that the statistical significance level, i.e., 1.65, shown as dashed lines, is equivalent to the 0.05 level before the

transformation. Refer to Supplementary Data 2 for estimated variance components of the four methods

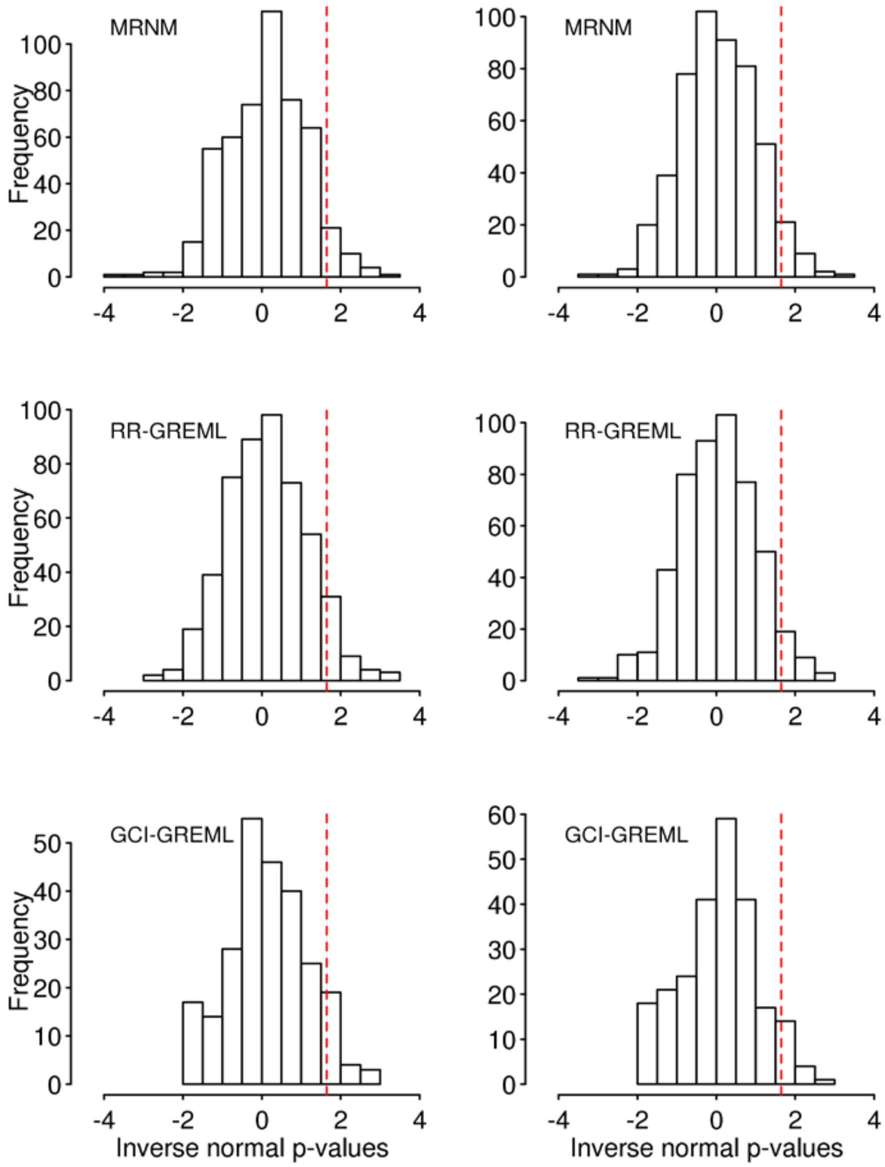

Supplementary Figure 9. Type I error rates of detecting genotype-covariate interaction for MRNM, RR-GREML and GCI-GREML.

Five hundred replicates of data were simulated under a model that assumed no genotype-covariate interaction but with the presence of genotype-covariate correlation and with the presence of residual-covariate correlation and interaction. Simulation was based QCed ARIC data consisting of 7,263 individuals and 583,058 SNPs. The models are specified as $\mathbf{y} = \boldsymbol{\alpha}_0 + \boldsymbol{\tau}_0 + \boldsymbol{\tau}_1 \times \mathbf{c}$ with $\mathbf{c} = \boldsymbol{\beta} + \boldsymbol{\varepsilon}$, all effects drawn from a multivariate normal distribution, where the

variance-covariance structure between $\boldsymbol{\alpha_0}$ and $\boldsymbol{\beta}$ is $\begin{bmatrix} 1 & 0.5 \\ 0.5 & 1 \end{bmatrix}$ and that between $\boldsymbol{\tau_0}$, $\boldsymbol{\varepsilon}$ and $\boldsymbol{\tau_1}$ is $\begin{bmatrix} 1 & 0.3 & 0.05 \\ 0.3 & 1 & 0 \\ 0.05 & 0 & \mathrm{var}(\boldsymbol{\tau_1}) \end{bmatrix}$ with var($\boldsymbol{\tau_1}$) = 0.25 (left panels) or 1 (right panels). For every replicate, each of three methods, i.e., MRNM, RR-GREML and GCI-GREML, was applied to obtain a p-value for detecting a G-C interaction via a comparison between the null (H_0) and alternative hypothesis (H_1) models. For MRNM, the H_0 and H_1 models were $\mathbf{y} = \boldsymbol{\alpha_0} + \boldsymbol{\tau_0} + \boldsymbol{\tau_1}\times\mathbf{c}$ with $\mathbf{c} = \boldsymbol{\beta} + \boldsymbol{\varepsilon}$ and $\mathbf{y} = \boldsymbol{\alpha_0} + \boldsymbol{\alpha_1}\times\mathbf{c} + \boldsymbol{\tau_0} + \boldsymbol{\tau_1}\times\mathbf{c}$ with $\mathbf{c} = \boldsymbol{\beta} + \boldsymbol{\varepsilon}$. For RR-GREML and GCI-GREML, the H_0 and H_1 models were $\mathbf{y} = \boldsymbol{\alpha_0} + \mathbf{e}$ and $\mathbf{y} = \boldsymbol{\alpha_0} + \boldsymbol{\alpha_1}\times\mathbf{c} + \mathbf{e}$. In RR-GREML and GCI-GREML, samples were arbitrarily stratified into four different groups according to the covariate levels. RR-GREML explicitly estimates residual variance for each of the four groups whereas GCI-GREML assumes homogeneous residual variance across the four groups and estimates a single residual variance. This figure shows the proportions of significant p-values, i.e., type I error rate, for each method (top to bottom) under the two simulation scenarios (left versus right). For var($\boldsymbol{\tau_1}$) = 0.25 (left panels), type I error rates from top to bottom are 0.060, 0.068, and 0.044, respectively. For var($\boldsymbol{\tau_1}$) = 1 (right panels), type I error rates from top to bottom are 0.048, 0.050, and 0.03, respectively. Note that p-values are inverse normal transformed, such that the statistical significance level, i.e., 1.65, shown as dashed lines, is equivalent to the 0.05 level before the transformation. Refer to Supplementary Data 3 for estimated variance components of the three methods.

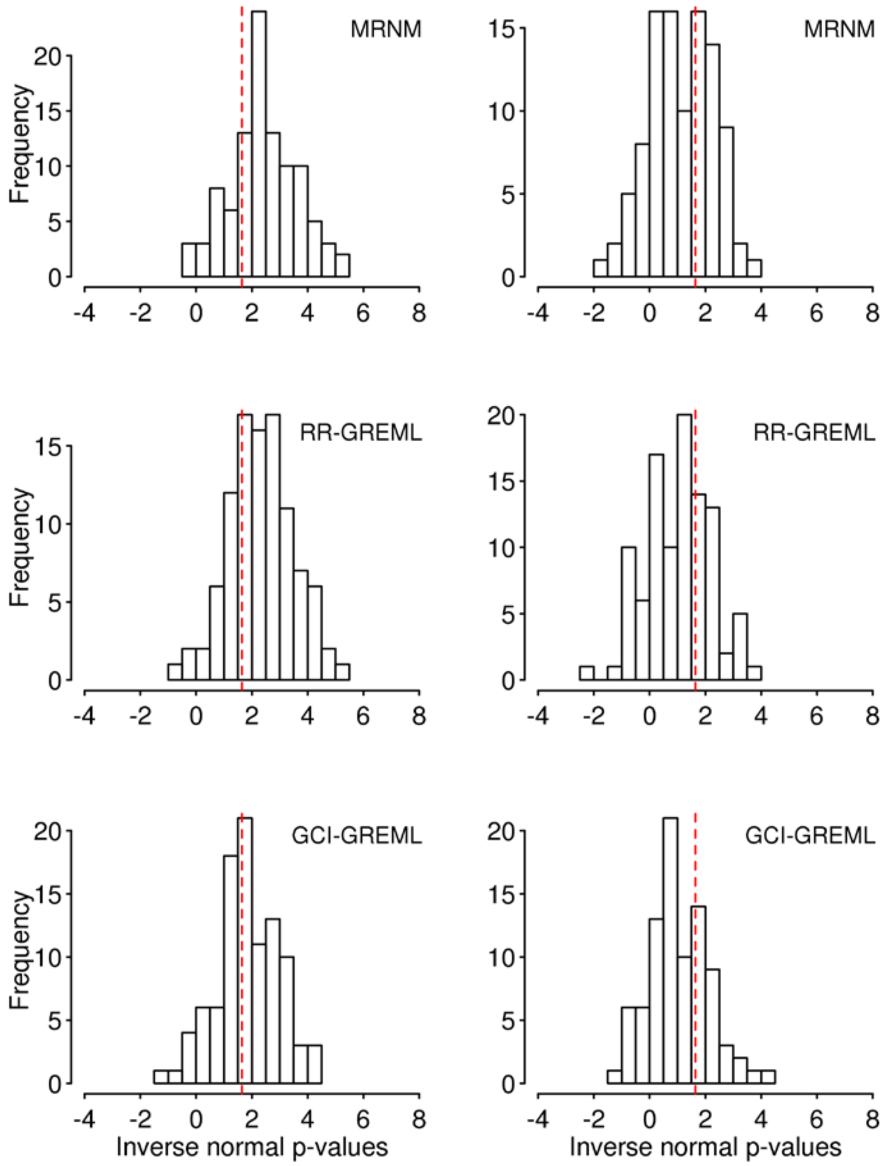

Supplementary Figure 10. Power of detecting genotype-covariate interaction for MRNM, RR-GREML and GCI-GREML.

One hundred replicates of data were simulated under a model that assumed the presence of genotype-covariate correlation and interaction and the presence of residual-covariate correlation and interaction. Simulation was based QCed ARIC data consisting of 7,263 individuals and 583,058 SNPs. The models are specified as $\mathbf{y} = \boldsymbol{\alpha}_0 + \boldsymbol{\alpha}_1 \times \mathbf{c} + \boldsymbol{\tau}_0 + \boldsymbol{\tau}_1 \times \mathbf{c}$ with $\mathbf{c} = \boldsymbol{\beta} + \boldsymbol{\varepsilon}$, all effects drawn from a multivariate normal distribution, where variance-covariance structure between $\boldsymbol{\alpha}_0$,

$\boldsymbol{\beta}$, and $\boldsymbol{\alpha_1}$ (in this order) is $\begin{bmatrix} 1 & 0.5 & 0.05 \\ 0.5 & 1 & 0 \\ 0.05 & 0 & 0.25 \end{bmatrix}$ and that between $\boldsymbol{\tau_0}$, $\boldsymbol{\varepsilon}$ and $\boldsymbol{\tau_1}$ is

$\begin{bmatrix} 1 & 0.3 & 0.05 \\ 0.3 & 1 & 0 \\ 0.05 & 0 & \text{var}(\boldsymbol{\tau_1}) \end{bmatrix}$ with var($\boldsymbol{\tau_1}$) = 0.25 (left panels) and 1 (right panels). For every replicate, each of three methods, i.e., MRNM, RR-GREML and GCI-GREML, was applied to obtain a p-value for detecting a G-C interaction via a comparison between the null (H_0) and alternative hypothesis (H_1) models. For MRNM, the H_0 and H_1 models were $\mathbf{y} = \boldsymbol{\alpha_0} + \boldsymbol{\tau_0} + \boldsymbol{\tau_1}\times\mathbf{c}$ with $\mathbf{c} = \boldsymbol{\beta} + \boldsymbol{\varepsilon}$ and $\mathbf{y} = \boldsymbol{\alpha_0} + \boldsymbol{\alpha_1}\times\mathbf{c} + \boldsymbol{\tau_0} + \boldsymbol{\tau_1}\times\mathbf{c}$ with $\mathbf{c} = \boldsymbol{\beta} + \boldsymbol{\varepsilon}$. For RR-GREML and GCI-GREML, the H_0 and H_1 models were $\mathbf{y} = \boldsymbol{\alpha_0} + \mathbf{e}$ and $\mathbf{y} = \boldsymbol{\alpha_0} + \boldsymbol{\alpha_1}\times\mathbf{c} + \mathbf{e}$. In RR-GREML and GCI-GREML, samples were arbitrarily stratified into four different groups according to the covariate levels. RR-GREML explicitly estimates residual variance for each of the four groups whereas GCI-GREML assumes homogeneous residual variance across the four groups and estimates a single residual variance. This figure shows the proportions of significant p-values, i.e., power, for each method (top to bottom) under the two simulation scenarios (left versus right). For var($\boldsymbol{\tau_1}$) = 0.25 (left panel), power from top to bottom are 0.77, 0.70, and 0.58, respectively. For var($\boldsymbol{\tau_1}$) = 1 (right panel), power from top to bottom are 0.35, 0.31 and 0.28, respectively. Note that p-values are inverse normal transformed, such that the statistical significance level, i.e., 1.65, shown as dashed lines, is equivalent to the 0.05 level before the transformation. Refer to Supplementary Data 4 for estimated variance components of the three methods.

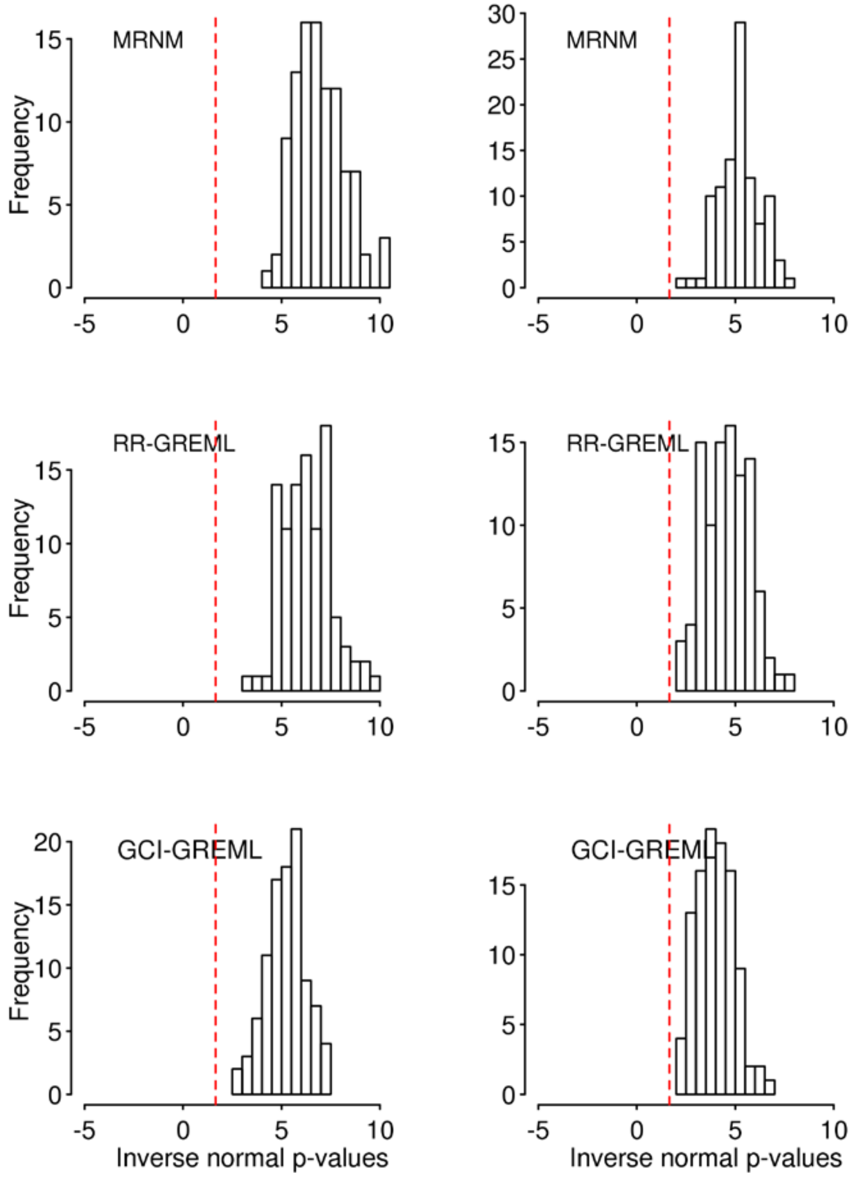

Supplementary Figure 11. Power of detecting genotype-covariate interaction for MRNM, RR-GREML and GCI-GREML.

One hundred replicates of data were simulated under a model that assumed the presence of genotype-covariate correlation and interaction and the presence of residual-covariate correlation and interaction. Simulation was based QCed ARIC data consisting of 7,263 individuals and 583,058 SNPs. The models are specified as $\mathbf{y} = \boldsymbol{\alpha}_0 + \boldsymbol{\alpha}_1 \times \mathbf{c} + \boldsymbol{\tau}_0 + \boldsymbol{\tau}_1 \times \mathbf{c}$ with $\mathbf{c} = \boldsymbol{\beta} + \boldsymbol{\varepsilon}$, all effects drawn from a multivariate normal distribution, where variance-covariance structure between $\boldsymbol{\alpha}_0$,

$\boldsymbol{\beta}$, and $\boldsymbol{\alpha_1}$ is $\begin{bmatrix} 1 & 0.5 & 0.05 \\ 0.5 & 1 & 0 \\ 0.05 & 0 & 1 \end{bmatrix}$ and that between $\boldsymbol{\tau_0}$, $\boldsymbol{\varepsilon}$ and $\boldsymbol{\tau_1}$ is $\begin{bmatrix} 1 & 0.3 & 0.05 \\ 0.3 & 1 & 0 \\ 0.05 & 0 & \mathrm{var}(\boldsymbol{\tau_1}) \end{bmatrix}$ with $\mathrm{var}(\boldsymbol{\tau_1})$ = 0.25 (left panels) or 1 (right panels). For every replicate, each of three methods, i.e., MRNM, RR-GREML and GCI-GREML, was applied separately to obtain a p-value for detecting a G-C interaction via a comparison between the null (H_0) and alternative hypothesis (H_1) models. For MRNM, the H_0 and H_1 models were $\mathbf{y} = \boldsymbol{\alpha_0} + \boldsymbol{\tau_0} + \boldsymbol{\tau_1}\times\mathbf{c}$ with $\mathbf{c} = \boldsymbol{\beta} + \boldsymbol{\varepsilon}$ and $\mathbf{y} = \boldsymbol{\alpha_0} + \boldsymbol{\alpha_1}\times\mathbf{c} + \boldsymbol{\tau_0} + \boldsymbol{\tau_1}\times\mathbf{c}$ with $\mathbf{c} = \boldsymbol{\beta} + \boldsymbol{\varepsilon}$. For RR-GREML and GCI-GREML, the H_0 and H_1 models were $\mathbf{y} = \boldsymbol{\alpha_0} + \mathbf{e}$ and $\mathbf{y} = \boldsymbol{\alpha_0} + \boldsymbol{\alpha_1}\times\mathbf{c} + \mathbf{e}$. In RR-GREML and GCI-GREML, samples were arbitrarily stratified into four different groups according to the covariate levels. RR-GREML explicitly estimates residual variance for each of the four groups whereas GCI-GREML assumes homogeneous residual variance across the four groups and estimates a single residual variance. This figure shows the proportions of p-values that are statistically significant, i.e., power, for each method (top to bottom) under the two simulation scenarios (left versus right). For $\mathrm{var}(\boldsymbol{\tau_1})$ = 0.25 (left panels), power from top to bottom are 1, 1, and 1, respectively. For $\mathrm{var}(\boldsymbol{\tau_1})$ = 1 (right panels), power from top to bottom are 1, 1 and 1, respectively. Note that p-values are inverse normal transformed, such that the statistical significance level, i.e., 1.65, shown as dashed lines, is equivalent to the 0.05 level before the transformation. Refer to Supplementary Data 5 for estimated variance components of the three methods.

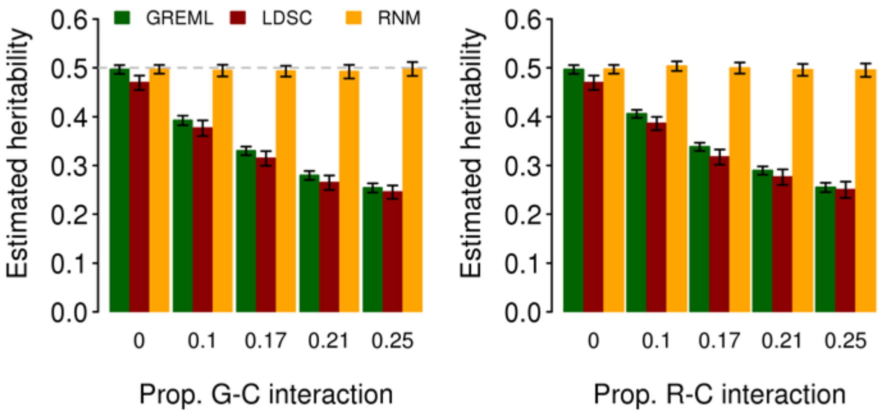

Supplementary Figure 12. Estimated heritability from data simulated under G-C (left) or R-C interaction model (right), using GREML, LDSC and RNM.

Prop. G-C or R-C interaction is the proportion of variance due to $\boldsymbol{\alpha}_1$ or $\boldsymbol{\tau}_1$ (see below) in the total phenotypic variance (i.e. var($\boldsymbol{\alpha}_1$))/var(**y**) in G-C interaction model or var($\boldsymbol{\tau}_1$)/var(**y**) in R-C interaction model).

Simulation was based QCed ARIC data consisting of 7,263 individuals and 583,058 SNPs.

Simulation for G-C interaction ($\boldsymbol{\alpha}_1$): The phenotype data were generated using $\mathbf{y} = \boldsymbol{\alpha}_0 + \boldsymbol{\alpha}_1 \times \mathbf{c} + \mathbf{e}$ with $\mathbf{c} = \boldsymbol{\beta} + \boldsymbol{\varepsilon}$, all effects drawn from a multivariate normal distribution. The variance-covariance structure of $\boldsymbol{\alpha}_0$, $\boldsymbol{\beta}$, and $\boldsymbol{\alpha}_1$ (in this order) is $\begin{bmatrix} 1 & 0 & 0.05 \\ 0 & 1 & 0 \\ 0.05 & 0 & \mathrm{var}(\boldsymbol{\alpha}_1) \end{bmatrix}$ with var($\boldsymbol{\alpha}_1$) = 0, 0.25, 0.5, 0.75 and 1 and that for **e** and **ε** is $\begin{bmatrix} 1 & 0 \\ 0 & 1 \end{bmatrix}$.

Simulation for R-C interaction ($\boldsymbol{\tau}_1$): The phenotype data were generated using $\mathbf{y} = \boldsymbol{\alpha}_0 + \boldsymbol{\tau}_0 + \boldsymbol{\tau}_1 \times \mathbf{c}$ with $\mathbf{c} = \boldsymbol{\beta} + \boldsymbol{\varepsilon}$, all effects drawn from a multivariate normal distribution. The variance-covariance structure of $\boldsymbol{\alpha}_0$ and $\boldsymbol{\beta}$ is $\begin{bmatrix} 1 & 0 \\ 0 & 1 \end{bmatrix}$ and that of $\boldsymbol{\tau}_0$, **ε**, and $\boldsymbol{\tau}_1$ (in this order) is $\begin{bmatrix} 1 & 0 & 0.05 \\ 0 & 1 & 0 \\ 0.05 & 0 & \mathrm{var}(\boldsymbol{\tau}_1) \end{bmatrix}$ with var($\boldsymbol{\tau}_1$) = 0, 0.25, 0.5, 0.75 and 1.

The error bar is a 95% confidence interval, which was estimated over 100 replicates.

The model for GREML is $\mathbf{y} = \boldsymbol{\alpha}_0 + \mathbf{e}$ and the model for RNM in the left panel is $\mathbf{y} = \boldsymbol{\alpha}_0 + \boldsymbol{\alpha}_1 \times \mathbf{c} + \mathbf{e}$.

The model for RNM in the right panel is $\mathbf{y} = \boldsymbol{\alpha}_0 + \boldsymbol{\tau}_0 + \boldsymbol{\tau}_1 \times \mathbf{c}$.

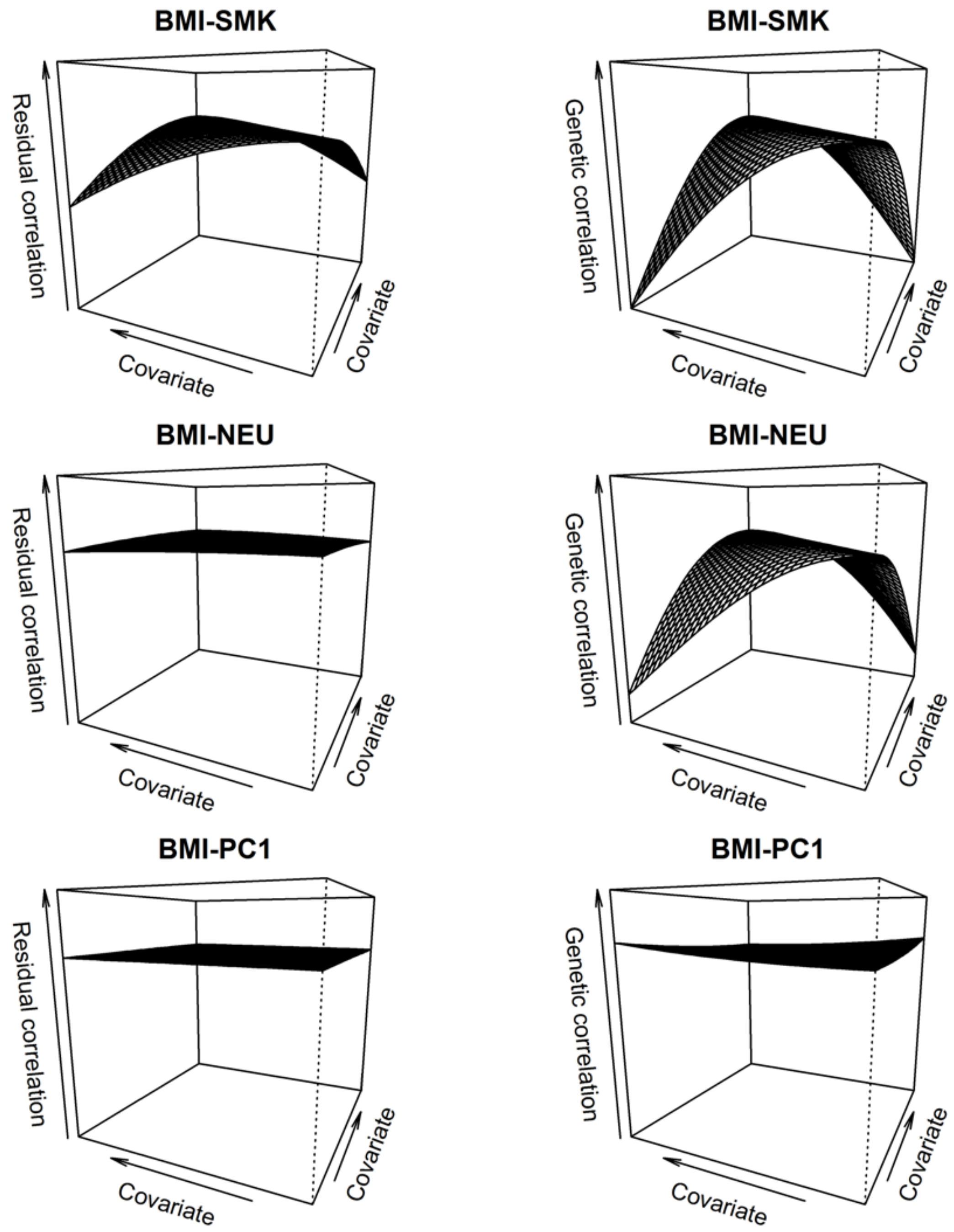

Supplementary Figure 13. Genetic and residual correlations between different covariate levels calculated based on the V_g and R_e matrices in Figures 6 and 7.

The scale of Z-axis for graph between 0.7 and 1.1.

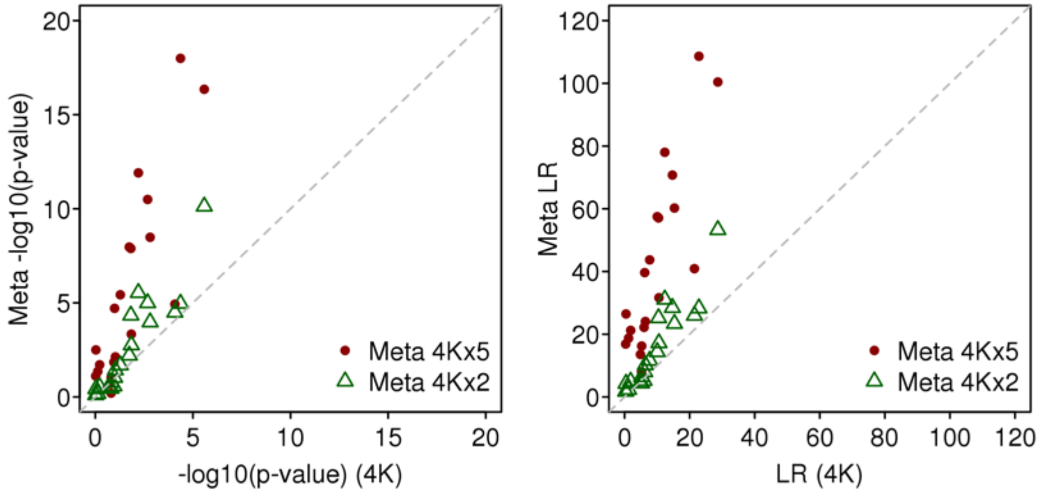

Supplementary Figure 14. Comparisons of –log10(p-value) and likelihood ratio for the meta-analyses across multiple studies against those for the analyses of individual studies.

The solid circles were based on meta-analyses of five studies each with 4,000 individuals.

The green triangles were based on meta-analyses of two studies each with 4,000 individuals.

The grey dashed line is where y = x.

The simulation was based on the first release of UKBB data (QCed), including 72,417 individuals and 1,009,054 SNPs, in which 10k SNPs were used as causal SNPs.

The simulated phenotypes were generated as $\mathbf{y} = \boldsymbol{\alpha}_0 + \boldsymbol{\alpha}_1 \times \mathbf{c} + \boldsymbol{\tau}_0 + \boldsymbol{\tau}_1 \times \mathbf{c}$ with $\mathbf{c} = \boldsymbol{\beta} + \boldsymbol{\varepsilon}$, all effects drawn from a multivariate normal distribution. The variance-covariance structure of $\boldsymbol{\alpha}_0$, $\boldsymbol{\beta}$, and $\boldsymbol{\alpha}_1$ is $\begin{bmatrix} 1 & 0.5 & 0 \\ 0.5 & 1 & 0 \\ 0 & 0 & \text{var}(\alpha_1) \end{bmatrix}$ where var($\boldsymbol{\alpha}_1$) was randomly sampled from a uniform distribution (U(0,1)). The variance-covariance structure of $\boldsymbol{\tau}_0$, $\boldsymbol{\varepsilon}$, and $\boldsymbol{\tau}_1$ is $\begin{bmatrix} 1 & 0.3 & 0.05 \\ 0.3 & 1 & 0 \\ 0.05 & 0 & 0.25 \end{bmatrix}$.

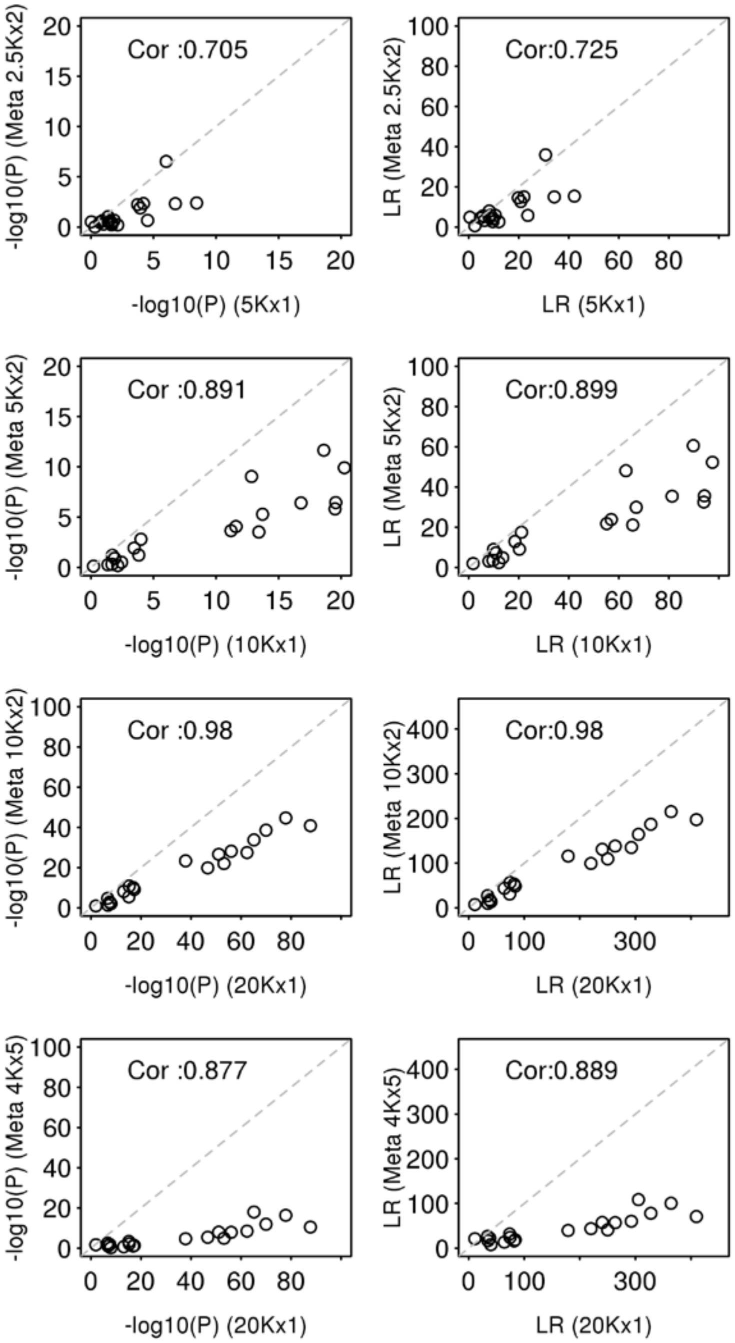

Supplementary Figure 15. Comparisons of –log10(p-value) and likelihood ratio for the meta-analyses across multiple subgroups against those for the full analyses of the whole group.

The same simulation scheme was applied as in Figure S9. For the top three rows, the sample size increased from 5K to 10K and 20K where the sample was randomly selected and divided into two subgroups with an equal number. For the last row, the sample was randomly selected and divided into five subgroups with an equal number.

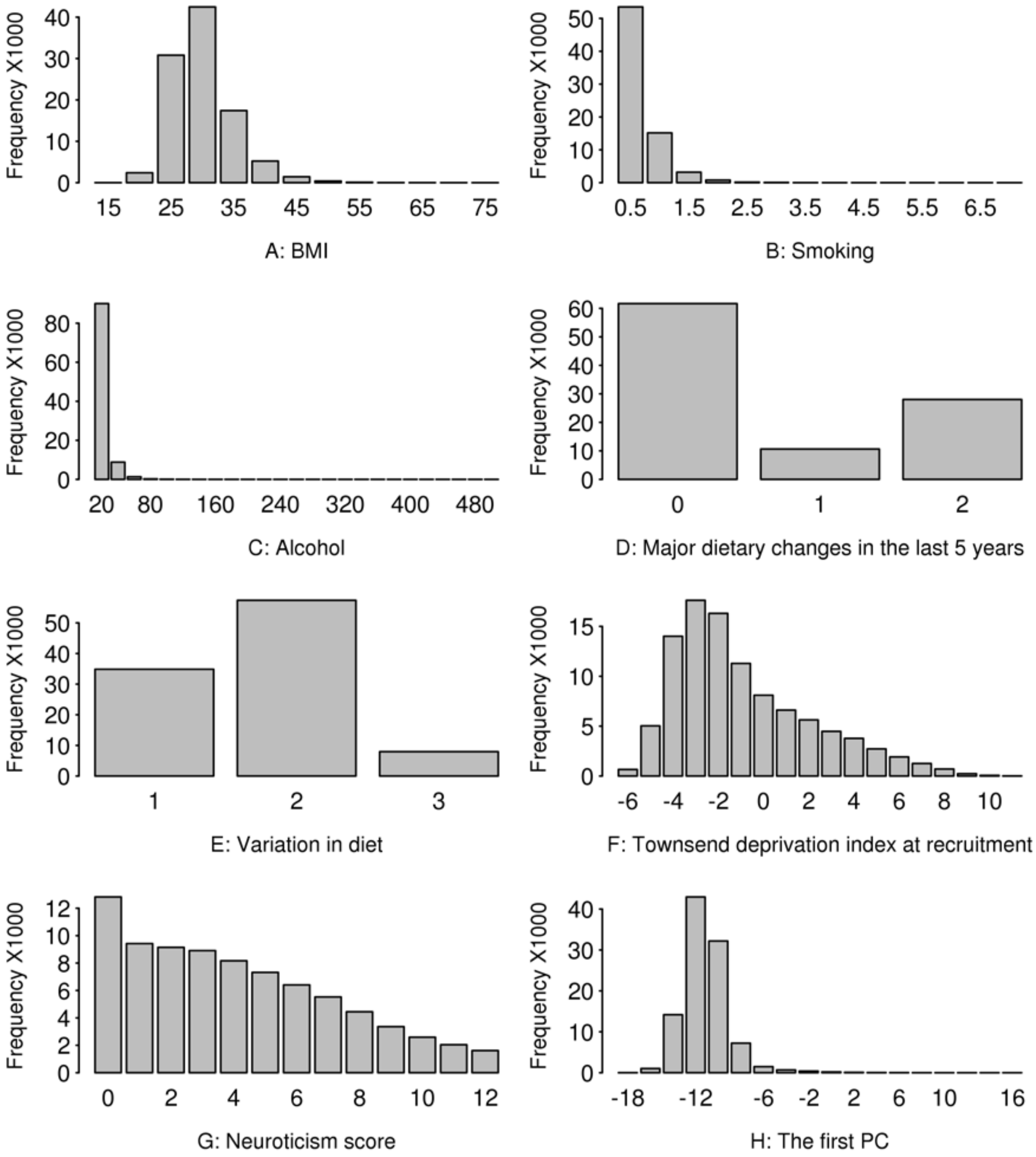

Supplementary Figure 16. Distributions of covariates of interest after quality control. A.BMI: Body mass index. B. Smoking: pack years of smoking. C. Alcohol: weekly alcohol consumption. D. Major dietary changes in the last 5 years. 0 is a generally stable diet. 1 is changing their diet due to health reasons. 2 is voluntarily changing their diet within five years prior to measurement. E. Variation in diet. 1 is Never or rarely. 2 is sometimes, 3 is often. F:

Townsend deprivation index at recruitment. G: Neuroticism score. 0 is the most optimistic, 12 is the least optimistic. H: the first principle component.

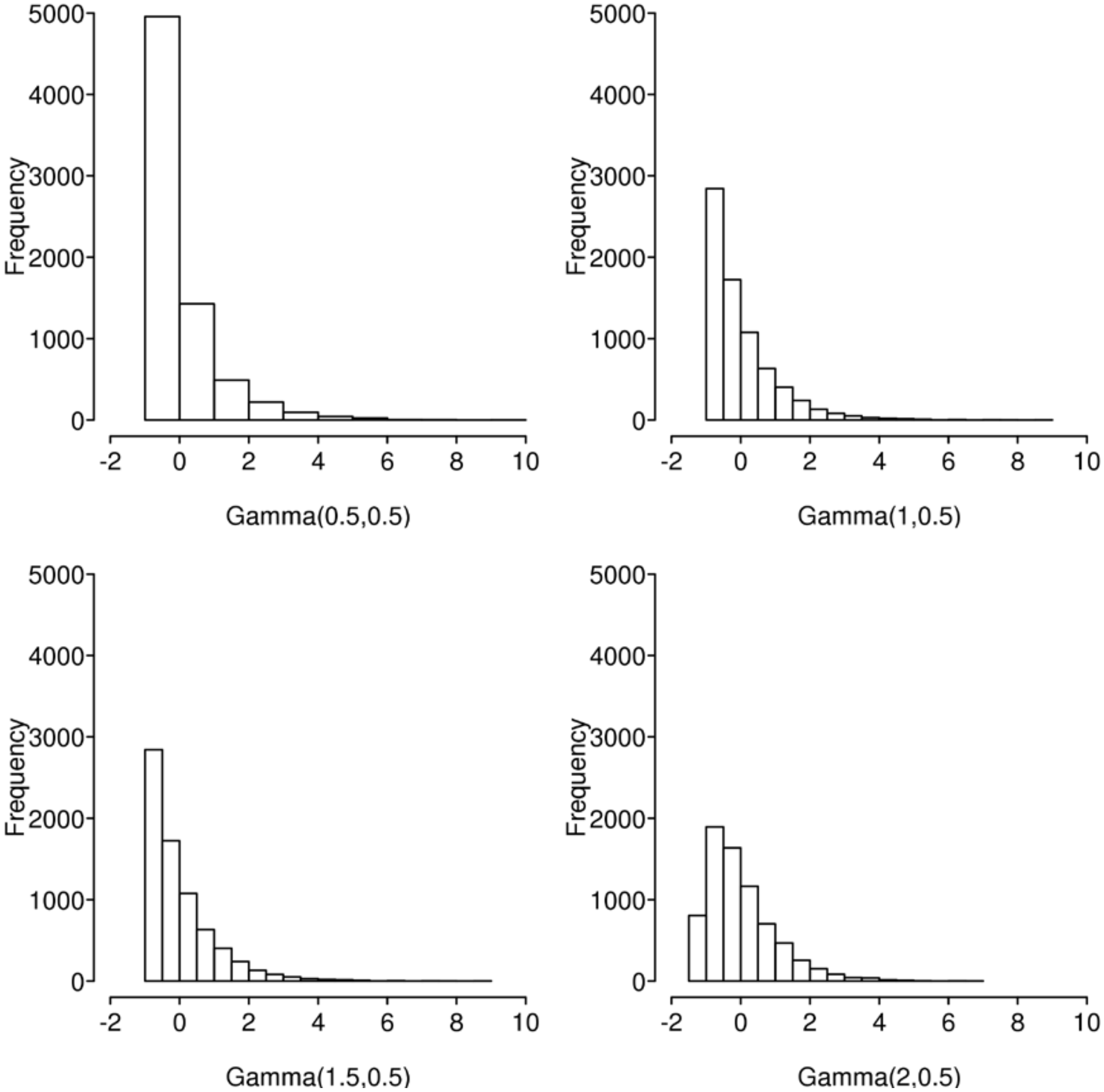

Supplementary Figure 17. The distribution of error values drawn from a gamma distribution with a shape parameter *k*=2, 1.5, 1 or 0.5 and scale parameter *θ*=0.5, denoted as Gamma(*k*, *θ*) in the figure.

Simulation detail and results based on these error values are in Supplementary Table 13.

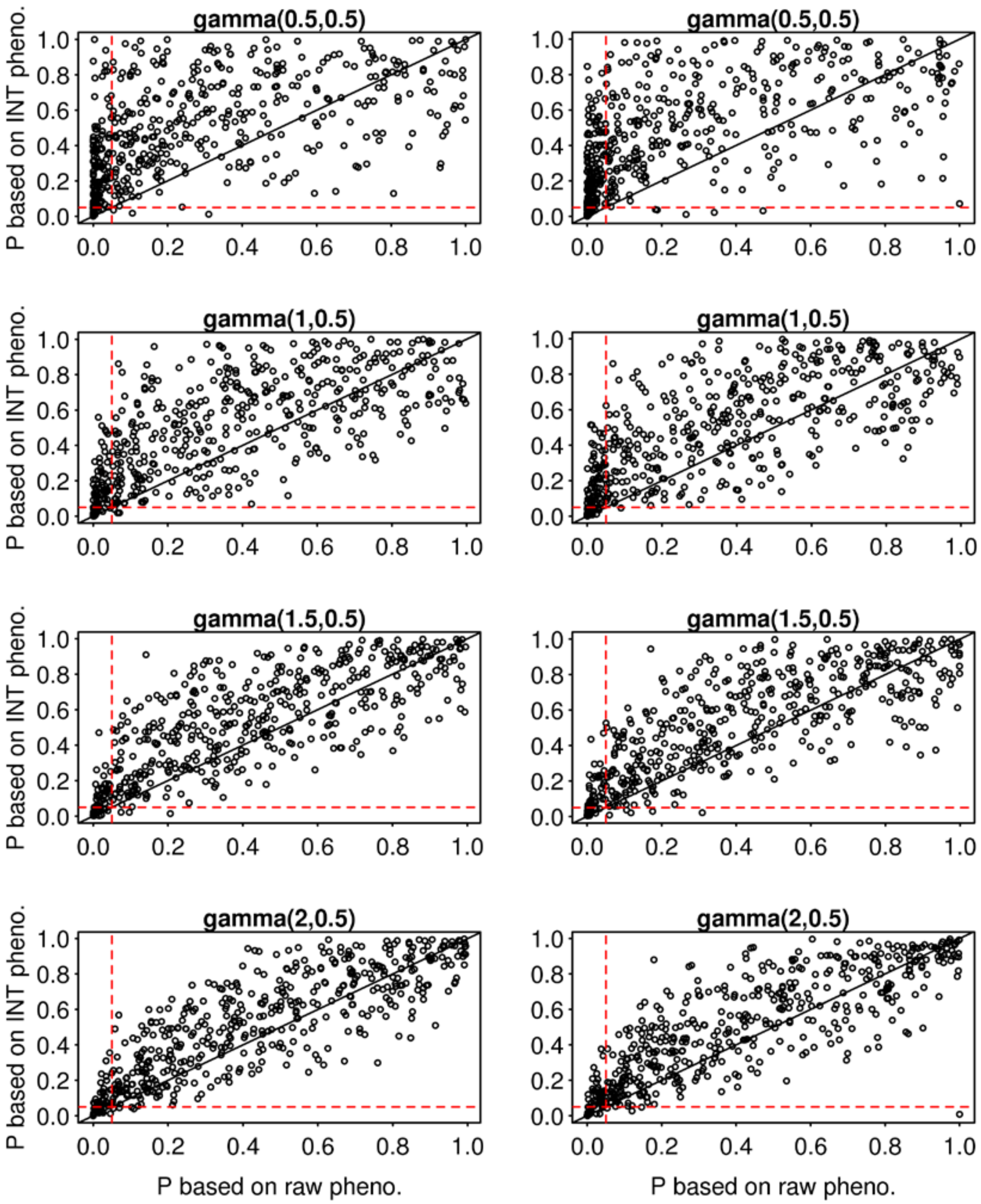

Supplementary Figure 18. P-values from likelihood ratio tests comparing null vs RNM G-C (left panel) and null vs RNM R-C (right panel) based on raw phenotypes and inverse normal transformed phenotypes in simulation.

Simulated data are the same used in Supplementary Table 13. Five hundred replicates of data were simulated under a null model that assumed no genotype-covariate correlation and no interaction. Simulation was based QCed ARIC data that have 7,263 individuals and 583,058

SNPs. The model is specified as $\mathbf{y} = \boldsymbol{\alpha_0} + \boldsymbol{\alpha_1} \times \mathbf{c} + \mathbf{e}$ with $\mathbf{c} = \boldsymbol{\beta} + \boldsymbol{\varepsilon}$, where $\boldsymbol{\alpha_0}$, $\boldsymbol{\beta}$, and $\boldsymbol{\alpha_1}$ (in this order) follows a multivariate normal distribution with mean zero and the variance-covariance structure being $\begin{bmatrix} 1 & 0 & 0 \\ 0 & 1 & 0 \\ 0 & 0 & 0 \end{bmatrix}$ and that between $\mathbf{e}$ and $\boldsymbol{\varepsilon}$ is $\begin{bmatrix} 1 & 0 \\ 0 & 1 \end{bmatrix}$. The residual error values are drawn from a gamma distribution with a shape parameter of 0.5, 1, 1.5 or 2. The simulated residual error values were further scaled as mean zero and variance 1. For each replicate, we fit Uni-GREML (as null model), RNM G-C, RNM R-C, and RNM Full models with rank-based inverse normal transformed phenotypes (denoted as 'INT') or with raw phenotypes (denoted as 'RAW').

The red dashed lines are where p-values equal 0.05.

Type I error rate for each comparison can be seen in Supplementary Table 13.

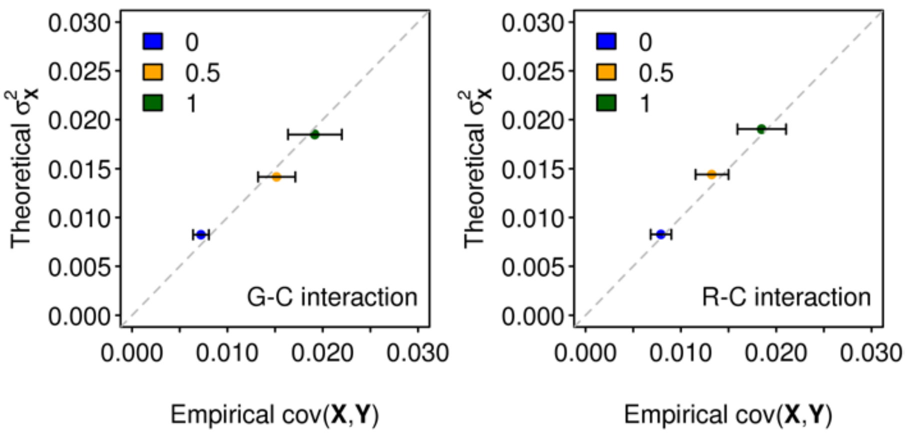

Supplementary Figure 19. Agreement between the theoretical variance of residual variance estimated from RNM and the empirical covariance between residual variances estimated from RNM and GREML.

$\mathbf{X}$ represents the estimated residual variance from RNM. $\mathbf{Y}$ represents the estimated residual variance from GREML. Theoretical σ_X^2 represents the variance of estimated residual variance from RNM (see Supplementary Note).

Different colors represent difference levels of interaction (i.e. variance of $\boldsymbol{\alpha_1}$ on the left panel and variance of $\boldsymbol{\tau_1}$ on the right panel).

The bars are 95% confidence interval, which were estimated based on the mean of 100 replicates of bootstrap. In each replicate, the bootstrap was performed 1000 times.

For the left panel, the simulated phenotypes were generated as $\mathbf{y} = \boldsymbol{\alpha_0} + \boldsymbol{\alpha_1} \times \mathbf{c} + \mathbf{e}$ with $\mathbf{c} = \boldsymbol{\beta} + \boldsymbol{\varepsilon}$, all effects drawn from a multivariate normal distribution. The variance-covariance structure of $\boldsymbol{\alpha_0}$, $\boldsymbol{\beta}$, and $\boldsymbol{\alpha_1}$ (in this order) is $\begin{bmatrix} 1 & 0 & 0.05 \\ 0 & 1 & 0 \\ 0.05 & 0 & \mathrm{var}(\boldsymbol{\alpha_1}) \end{bmatrix}$ with $\mathrm{var}(\boldsymbol{\alpha_1})$ = 0, 0.5 and 1 and that of $\mathbf{e}$ and $\boldsymbol{\varepsilon}$ is $\begin{bmatrix} 1 & 0 \\ 0 & 1 \end{bmatrix}$. The number of replicates is 500.

For the right panel, the simulated phenotype was generated as $\mathbf{y} = \boldsymbol{\alpha}_0 + \boldsymbol{\tau}_0 + \boldsymbol{\tau}_1 \times \mathbf{c}$ with $\mathbf{c} = \boldsymbol{\beta} + \boldsymbol{\varepsilon}$.

The variance-covariance structure of $\boldsymbol{\alpha}_0$, $\boldsymbol{\beta}$ is $\begin{bmatrix} 1 & 0 \\ 0 & 1 \end{bmatrix}$ and that of $\boldsymbol{\tau}_0$, $\boldsymbol{\varepsilon}$, and $\boldsymbol{\tau}_1$ is

$\begin{bmatrix} 1 & 0 & 0.05 \\ 0 & 1 & 0 \\ 0.05 & 0 & \mathrm{var}(\boldsymbol{\tau}_1) \end{bmatrix}$ with $\mathrm{var}(\boldsymbol{\tau}_1)$ =0, 0.5 and 1. The number of replicates is 500. Simulation was

based QCed ARIC data consisting of 7,263 individuals and 583,058 SNPs.

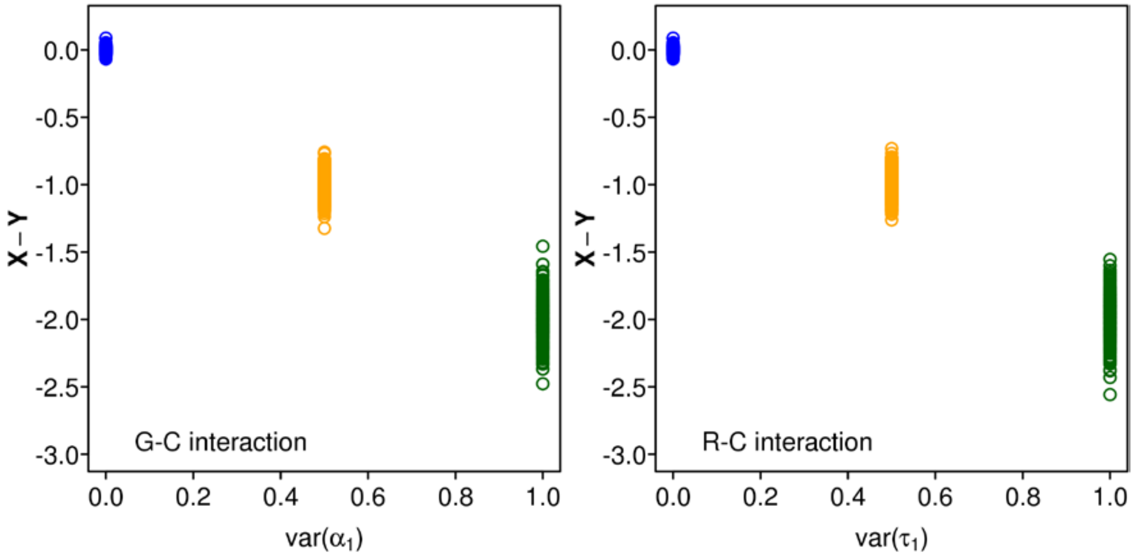

Supplementary Figure 20. Differences between estimated residual variance from RNM (X) and that from GREML (Y).

Simulated data are the same used in Supplementary Figure 19. In the x-axis, the true values used for var ($\boldsymbol{\alpha_1}$) and var ($\boldsymbol{\tau_1}$) are shown. In the y-axis, '**X-Y**' represents the difference between the estimated residual variances from RNM and GREML.

Different colors represent difference levels of simulated interaction variances (i.e. var ($\boldsymbol{\alpha_1}$) and var ($\boldsymbol{\tau_1}$)).

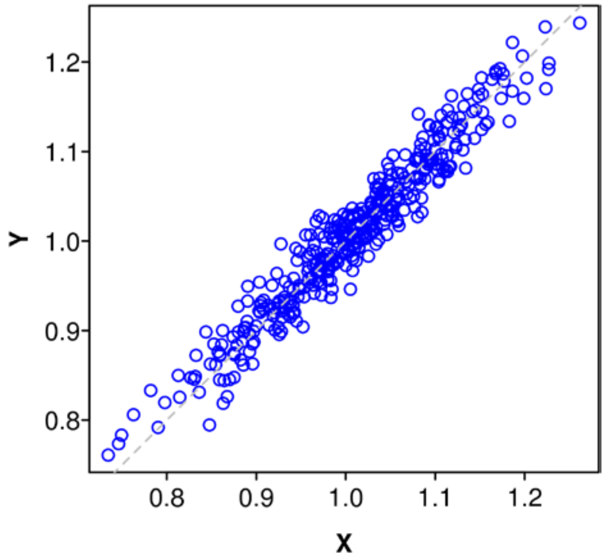

Supplementary Figure 21. Estimated residual variance from RNM (X) and that from GREML (Y) in a simulation with the absence of GCCI and RCCI.

The data used in this Figure are the same in Supplementary Figure 20 when var ($\boldsymbol{\alpha}_1$) and var ($\boldsymbol{\tau}_1$) are both zeros.

X represents the estimated residual variance from RNM. **Y** represents the estimated residual variance from GREML.

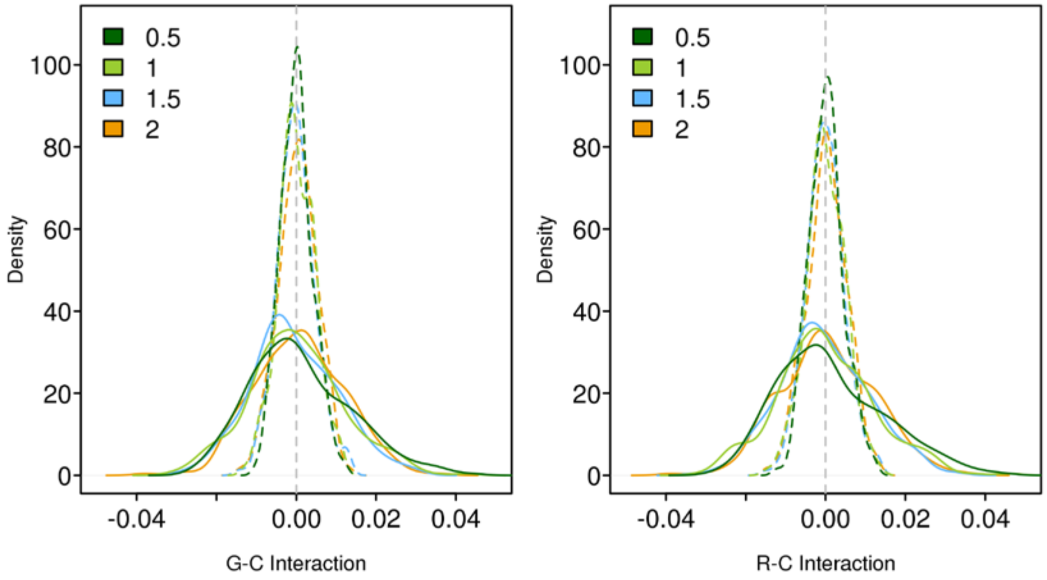

Supplementary Figure 22. Estimated G-C or R-C interaction variance under the null simulation model (i.e. no interaction) with residual error values drawn from a gamma distribution (with shape parameter of 0.5, 1, 1.5 or 2).

Simulation model detail is in Supplementary Table 13.

Different colors represent estimated interaction variances from the gamma distributions with different shape parameters (0.5, 1, 1.5 and 2).

The solid lines stand for the estimated interaction variances using raw phenotypes.

The dashed lines stand for the estimated interaction variances using rank-based inverse normal transformed phenotypes.

The vertical dashed lines stand for the true simulated interaction (x=0, i.e. no interaction).

G-C interaction was estimated from RNM G-C model (left panel), and R-C interaction was estimated from RNM R-C model (right panel). Results are very similar when using RNM Full model (considering G-C and R-C jointly) (results not shown).

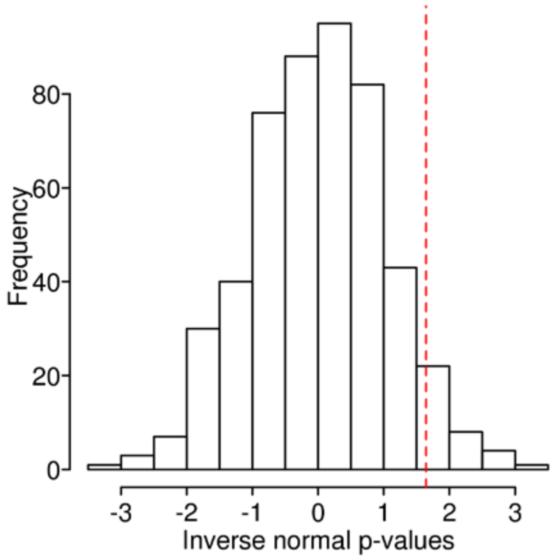

Supplementary Figure 23. Type I error rate for detecting G-C interaction under a null simulation with two covariates.

Five hundred replicates of data were simulated under a null model that assumed no genotype-covariate correlation and interaction between two covariates and phenotype. Simulation was based QCed ARIC data consisting of 7,263 individuals and 583,058 SNPs. The models of simulation are specified as $\mathbf{y} = \boldsymbol{\alpha_0} + \boldsymbol{\alpha_1}\times\mathbf{c_1} + \boldsymbol{\alpha_2}\times\mathbf{c_2} + \boldsymbol{\tau_0}$ with $\mathbf{c_1} = \boldsymbol{\beta_1} + \boldsymbol{\varepsilon_1}$ and $\mathbf{c_2} = \boldsymbol{\beta_2} + \boldsymbol{\varepsilon_2}$, all effects drawn from a multivariate normal distribution, where variance-covariance structure between $\boldsymbol{\alpha_0}$, $\boldsymbol{\beta_1}$, $\boldsymbol{\beta_2}$, $\boldsymbol{\alpha_1}$ and $\boldsymbol{\alpha_2}$ (in this order) is $\begin{bmatrix}1 & 0 & 0 & 0 & 0\\ 0 & 1 & 0.5 & 0 & 0\\ 0 & 0.5 & 1 & 0 & 0\\ 0 & 0 & 0 & 0 & 0\\ 0 & 0 & 0 & 0 & 0\end{bmatrix}$ and that between $\boldsymbol{\tau_0}$, $\boldsymbol{\varepsilon_1}$ and $\boldsymbol{\varepsilon_2}$ is $\begin{bmatrix}1 & 0 & 0\\ 0 & 1 & 0\\ 0 & 0 & 1\end{bmatrix}$. For each replicate, a p-value was obtained via a likelihood ratio test comparing the null (H_0) and alternative hypothesis (H_1) models. The H_0 and H_1 models were $\mathbf{y} = \boldsymbol{\alpha_0} + \boldsymbol{\tau_0}$ and $\mathbf{y} = \boldsymbol{\alpha_0} + \boldsymbol{\alpha_1}\times\mathbf{c_1} + \boldsymbol{\alpha_2}\times\mathbf{c_2} + \boldsymbol{\tau_0}$. The type I error rate was 0.056.

Refer Supplementary Table 18 for the estimated variances.

Supplementary Table 1. Summary of models used in this study.

Name	Brief feature	Model
Univariate interaction models		
RR-GREML	Allows for G-C interaction and discrete covariates, and estimates residual variance for each level	$\mathbf{T_1 = \alpha_0 + \alpha_1 \cdot c + e}$
GCI-GREML	Allows for G-C interaction and discrete covariates, and assumes homogeneous residual variance	$\mathbf{T_1 = \alpha_0 + \alpha_1 \cdot c + e}$
RNM Null model	Equivalent to univariate GREML	$\mathbf{T_1 = \alpha_0 + e}$
RNM R-C model	Allows for R-C interaction and continuous covariates, and assumes homogeneous genetic variance across different covariate levels	$\mathbf{T_1 = \alpha_0 + \tau_0 + \tau_1 \cdot c}$
RNM G-C model	Allows for G-C interaction and continuous covariates, and assumes homogeneous residual variance across different covariate levels	$\mathbf{T_1 = \alpha_0 + \alpha_1 \cdot c + e}$
RNM Full model	Allows for G-C and R-C interaction, and continuous covariates	$\mathbf{T_1 = \alpha_0 + \alpha_1 \cdot c + \tau_0 + \tau_1 \cdot c}$
**Multivariate interaction models**		
MRNM Null model	Equivalent to multivariate GREML	$\mathbf{T_1 = \alpha_0 + e}$<br>$\mathbf{T_2 = \beta + \varepsilon}$
MRNM R-C model	Allows for G-C correlation and R-C correlation and interaction (RCCI), and continuous covariates, and assumes homogeneous genetic variance across different covariate levels	$\mathbf{T_1 = \alpha_0 + \tau_0 + \tau_1 \cdot c}$<br>$\mathbf{T_2 = \beta + \varepsilon}$
MRNM G-C model	Allows for G-C correlation and interaction (GCCI) and R-C correlation, and continuous covariates, and assumes homogeneous residual variance across different covariate levels	$\mathbf{T_1 = \alpha_0 + \alpha_1 \cdot c + e}$<br>$\mathbf{T_2 = \beta + \varepsilon}$
MRNM Full model	Allows for GCCI and RCCI, and continuous covariates	$\mathbf{T_1 = \alpha_0 + \alpha_1 \cdot c + \tau_0 + \tau_1 \cdot c}$<br>$\mathbf{T_2 = \beta + \varepsilon}$

Note: T_1 is the residual of main trait adjusted for confounders. T_2 is the residual of c adjusted for confounders.

Supplementary Table 2. Estimated variance components of random regression coefficients from data simulated under a model that assumed the presence of genotype-covariate interaction.

**Parameters**	**var(e)**	**var(α_0)**	**var(α_1)**	**cov(α_0, α_1)**
**True value**	1.00	1.00	0.25	0.05
**RNM**	1.00 (0.011)[a]	1.00 (0.011)	0.25 (0.002)	0.05 (0.002)
**RR-GREML**[b]	1.17 (0.031)<br>1.00 (0.012)<br>0.98 (0.013)<br>1.20 (0.035)	2.01 (0.021)	0.50 (0.016)	0.10 (0.011)
**GCI-GREML**[c]	1.08 (0.022)	0.86 (0.013)	0.56 (0.023)	

[a]Standard errors (in brackets) of estimates from 100 replicates.

[b]In RR-GREML, samples were arbitrarily stratified into four different groups according to the covariate levels. RR-GREML explicitly estimated residual variance for each of the four groups. It is noted that RR-GREML used Legendre polynomial function such that the scale of the estimated var($\boldsymbol{\alpha}_0$) was different from RNM or GCI-GREML.

[c]In GCI-GREML, as in RR-GREML, samples were arbitrarily stratified into four different groups according to the covariate levels. GCI-GREML assumes homogeneous residual variance across the four groups and estimates a single residual variance.

Simulation was based QCed ARIC data consisting 7,263 individuals and 583,058 SNPs.

Supplementary Table 3. Variance components estimated based on a simulation with a relatively low genetic correlation

		var(τ0)	var(ε)	cov(τ0,ε)	var(α0)	var(α1)	cov(α0, α1)	var(β)	cov(α0, β)	cov(α1, β)
**GREML**	Est	1.012			0.990					
	SE	0.009			0.009					
**RNM G-C**	Est	0.980			0.990	0.016	0.000			
	SE	0.009			0.009	0.001	0.001			
**MVGREML**	Est	1.011	1.017	0.293	0.991			0.983	0.104	
	SE	0.009	0.008	0.007	0.009			0.009	0.007	
**MRNM G-C**	Est	1.014	1.017	0.293	0.991	-0.002	0.000	0.983	0.104	-0.001
	SE	0.009	0.008	0.007	0.009	0.001	0.001	0.009	0.007	0.001

One hundred replicates of data were simulated under a null model that assumed genotype-covariate correlation but no genotype-covariate interaction. The model is specified as $\mathbf{y} = \boldsymbol{\alpha}_0 + \boldsymbol{\alpha}_1 \times \mathbf{c} + \mathbf{e}$ with $\mathbf{c} = \boldsymbol{\beta} + \boldsymbol{\varepsilon}$, all effects drawn from a multivariate normal distribution, where the variance-covariance structure between $\boldsymbol{\alpha}_0$, $\boldsymbol{\beta}$, and $\boldsymbol{\alpha}_1$ (in this order) is $\begin{bmatrix} 1 & 0.1 & 0 \\ 0.1 & 1 & 0 \\ 0 & 0 & 0 \end{bmatrix}$ and that between $\mathbf{e}$ and $\boldsymbol{\varepsilon}$ is $\begin{bmatrix} 1 & 0.3 \\ 0.3 & 1 \end{bmatrix}$. For every replicate, a univariate RNM and a multivariate RNM were fitted separately to obtain a p-value for the G-C interaction by comparing the null (H_0) and alternative hypothesis (H_1) model. For the univariate RNM, the H_0 and H_1 models were $\mathbf{y} = \boldsymbol{\alpha}_0 + \mathbf{e}$ and $\mathbf{y} = \boldsymbol{\alpha}_0 + \boldsymbol{\alpha}_1 \times \mathbf{c} + \mathbf{e}$. For the multivariate RNM, the H_0 and H_1 models were $\mathbf{y} = \boldsymbol{\alpha}_0 + \mathbf{e}$ with $\mathbf{c} = \boldsymbol{\beta} + \boldsymbol{\varepsilon}$ and $\mathbf{y} = \boldsymbol{\alpha}_0 + \boldsymbol{\alpha}_1 \times \mathbf{c} + \mathbf{e}$ with $\mathbf{c} = \boldsymbol{\beta} + \boldsymbol{\varepsilon}$. This figure shows the proportions of significant p-values, i.e., type I error rate, for both models, which are 0.25 (univariate RNM) and 0.04 (multivariate RNM). Note that p-values are inverse normal transformed, such that the statistical significance level, i.e., 1.65, shown as dashed lines, is equivalent to the 0.05 level before the transformation. Refer Supplementary Figure 3 for the type I error.

Supplementary Table 4. Estimated variance components of random regression coefficients from data simulated under a model that assumes the presence of genotype-covariate correlation (i.e. cov(α_0, β) > 0) and interaction (i.e. var(α_1) > 0).

Parameters	var(e)	var(ε)	cov(e,ε)	var(α_0)	var(α_1)	cov(α_0, α_1)	var(β)	cov(α_0, β)	cov(α_1, β)
**True value**	1.00	1.00	0.30	1.00	0.25	0.05	1.00	0.50	0.00
**RNM**	0.93 (0.011)	N/A	N/A	0.95 (0.011)	0.31 (0.002)	0.05 (0.002)	N/A	N/A	N/A
**MRNM**	1.00 (0.011)	1.00 (0.009)	0.29 (0.008)	0.96 (0.011)	0.25 (0.002)	0.05 (0.002)	1.00 (0.009)	0.51 (0.008)	0.00 (0.002)

Estimates and standard errors (in brackets) are based on 100 replicates.

For RNM, the model was $\mathbf{y} = \boldsymbol{\alpha}_0 + \boldsymbol{\alpha}_1 \times \mathbf{c} + \mathbf{e}$. For MRNM, the model was $\mathbf{y} = \boldsymbol{\alpha}_0 + \boldsymbol{\alpha}_1 \times \mathbf{c} + \mathbf{e}$ with $\mathbf{c} = \boldsymbol{\beta} + \boldsymbol{\varepsilon}$, all effects drawn from a multivariate normal distribution.

Simulation was based QCed ARIC data consisting 7,263 individuals and 583,058 SNPs.

Supplementary Table 5. Estimated variance components (standard error) of random regression coefficients after adjusting for the covariance in the model with or without genotype–covariate interaction.

Parameters	var($\mathbf{e}$)	var($\boldsymbol{\alpha}_0$)	var($\boldsymbol{\alpha}_1$)	cov($\boldsymbol{\alpha}_0$, $\boldsymbol{\alpha}_1$)
Presence of genotype–covariate interaction				
True value	1.00	1.00	0.25	0.05
Estimates (SE)	0.919 (0.008)	0.757 (0.008)	0.252 (0.002)	0.049 (0.002)
Absence of genotype–covariate interaction				
True value	1.00	1.00	0	0
Estimates (SE)	0.929 (0.007)	0.751 (0.006)	0.001 (0.001)	0.001 (0.001)

Supplementary Table 6. Variance components estimated based on a simulation with non-zero cov(ε,τ_1) and cov(α_1, β)

var(τ_1)		var(τ_0)	var(ε)	cov(τ_0,ε)	var(τ_1)	cov(τ_0,τ_1)	cov(ε,τ_1)	var(α_0)	var(α_1)	cov(α_0, α_1)	var(β)	cov(α_0, β)	cov(α_1, β)
**True**		1	1	0.3	0.25 or 1	0.05	0.1	1	1	0.05	1	0.5	0.1
**0.25**	Est	1.029	1.007	0.315	0.263	0.055	0.110	0.967	0.240	0.047	0.996	0.488	0.093
	SE	0.022	0.017	0.013	0.012	0.012	0.012	0.021	0.012	0.013	0.017	0.015	0.013
**1**	Est	1.014	1.017	0.322	0.982	0.041	0.095	0.966	0.269	0.048	0.985	0.466	0.109
	SE	0.017	0.017	0.014	0.024	0.015	0.013	0.017	0.021	0.016	0.017	0.014	0.014

The models are specified as $\mathbf{y} = \boldsymbol{\alpha}_0 + \boldsymbol{\alpha}_1\times\mathbf{c} + \boldsymbol{\tau}_0 + \boldsymbol{\tau}_1\times\mathbf{c}$ with $\mathbf{c} = \boldsymbol{\beta} + \boldsymbol{\varepsilon}$, all effects drawn from a multivariate normal distribution, where variance-covariance structure between $\boldsymbol{\alpha}_0$, $\boldsymbol{\beta}$, and $\boldsymbol{\alpha}_1$ (in this order) is $\begin{bmatrix} 1 & 0.5 & 0.05 \\ 0.5 & 1 & 0.1 \\ 0.05 & 0.1 & 0.25 \end{bmatrix}$ and that between $\boldsymbol{\tau}_0$, $\boldsymbol{\varepsilon}$ and $\boldsymbol{\tau}_1$ is

$\begin{bmatrix} 1 & 0.3 & 0.05 \\ 0.3 & 1 & 0.1 \\ 0.05 & 0.1 & \mathrm{var}(\boldsymbol{\tau}_1) \end{bmatrix}$ with var($\boldsymbol{\tau}_1$) = 0.25 and 1.

The standard errors were based in 100 replicates.

Supplementary Table 7. Estimated residual and genetic variances from data simulated under G-C (left) or R-C interaction model (right) with the presence of genetic correlation, using MVGREML, LDSC and MRNM

	G-C interaction								R-C interaction							
**%Interaction**[a]	var(e)		var(α_0)		h^2		r_g		var(e)		var(α_0)		h^2		r_g	
	Est	SE	Est	SE	Est	SE	Est	SE	Est	SE	Est	SE	Est	SE	Est	SE
**MVGREML**																
**0**	1.00	0.02	0.99	0.02	0.50	0.01	0.50	0.01	1.00	0.01	1.00	0.01	0.50	0.00	0.50	0.01
**0.1**	1.54	0.02	0.97	0.02	0.39	0.01	0.51	0.01	1.49	0.01	1.01	0.01	0.41	0.00	0.49	0.01
**0.17**	2.00	0.03	1.01	0.04	0.34	0.01	0.52	0.02	1.99	0.02	1.01	0.01	0.34	0.00	0.49	0.01
**0.21**	2.42	0.03	1.07	0.03	0.31	0.01	0.51	0.01	2.50	0.02	1.00	0.02	0.29	0.00	0.50	0.01
**0.25**	2.98	0.05	1.04	0.05	0.26	0.01	0.51	0.01	3.03	0.02	0.99	0.02	0.25	0.00	0.51	0.01
**LDSC**																
**0**	1.05	0.04	0.95	0.04	0.47	0.02	0.50	0.02	1.03	0.02	0.97	0.02	0.48	0.01	0.51	0.01
**0.1**	1.53	0.04	0.97	0.04	0.39	0.01	0.53	0.02	1.49	0.02	1.01	0.02	0.40	0.01	0.48	0.01
**0.17**	2.00	0.05	1.00	0.05	0.33	0.02	0.53	0.02	2.02	0.02	0.98	0.02	0.33	0.01	0.48	0.01
**0.21**	2.42	0.05	1.08	0.05	0.31	0.01	0.52	0.03	2.55	0.03	0.95	0.03	0.27	0.01	0.49	0.01
**0.25**	3.02	0.07	0.98	0.07	0.25	0.02	0.50	0.02	3.07	0.02	0.93	0.02	0.23	0.01	0.50	0.02
**MRNM**																
**0**	1.00	0.02	1.00	0.02	0.50	0.01	0.50	0.01	1.00	0.01	1.00	0.01	0.50	0.01	0.50	0.01
**0.1**	1.01	0.02	0.99	0.02	0.49	0.01	0.50	0.02	0.99	0.01	1.01	0.01	0.52	0.01	0.50	0.01
**0.17**	1.00	0.03	1.01	0.03	0.50	0.02	0.52	0.01	1.00	0.01	1.00	0.01	0.52	0.01	0.49	0.01
**0.21**	0.96	0.03	1.06	0.03	0.53	0.01	0.51	0.01	1.02	0.01	0.98	0.01	0.49	0.01	0.50	0.01
**0.25**	0.98	0.03	1.03	0.04	0.51	0.02	0.51	0.01	1.00	0.01	1.00	0.01	0.51	0.01	0.50	0.01

[a]The proportion of variance due to $\boldsymbol{\alpha_1}$ or $\boldsymbol{\tau_1}$ (see below) in the total phenotypic variance (i.e. var($\boldsymbol{\alpha_1}$))/var(**y**) in G-C interaction model or var($\boldsymbol{\tau_1}$)/var(**y**) in R-C interaction model).

Simulation for G-C interaction ($\boldsymbol{\alpha_1}$): The phenotype data were generated using $\mathbf{y} = \boldsymbol{\alpha_0} + \boldsymbol{\alpha_1} \times \mathbf{c} + \mathbf{e}$ with $\mathbf{c} = \boldsymbol{\beta} + \boldsymbol{\varepsilon}$, all effects drawn from a multivariate normal distribution. The variance-covariance structure of $\boldsymbol{\alpha_0}$, $\boldsymbol{\beta}$, and $\boldsymbol{\alpha_1}$ (in this order) is $\begin{bmatrix} 1 & 0.5 & 0.05 \\ 0.5 & 1 & 0 \\ 0.05 & 0 & \mathrm{var}(\boldsymbol{\alpha_1}) \end{bmatrix}$ with var($\boldsymbol{\alpha_1}$) = 0, 0.25, 0.5, 0.75 and 1 and that for **e** and $\boldsymbol{\varepsilon}$ is $\begin{bmatrix} 1 & 0 \\ 0 & 1 \end{bmatrix}$.

Simulation for R-C interaction ($\boldsymbol{\tau_1}$): The phenotype data were generated using $\mathbf{y} = \boldsymbol{\alpha_0} + \boldsymbol{\tau_0} + \boldsymbol{\tau_1} \times \mathbf{c}$ with $\mathbf{c} = \boldsymbol{\beta} + \boldsymbol{\varepsilon}$, all effects drawn from a multivariate normal distribution. The variance-covariance structure of $\boldsymbol{\alpha_0}$ and $\boldsymbol{\beta}$ is $\begin{bmatrix} 1 & 0.5 \\ 0.5 & 1 \end{bmatrix}$ and that of $\boldsymbol{\tau_0}$, $\boldsymbol{\varepsilon}$, and $\boldsymbol{\tau_1}$ (in this order) is $\begin{bmatrix} 1 & 0 & 0.05 \\ 0 & 1 & 0 \\ 0.05 & 0 & \mathrm{var}(\boldsymbol{\tau_1}) \end{bmatrix}$ with var($\boldsymbol{\tau_1}$) = 0, 0.25, 0.5, 0.75 and 1.

The standard errors were estimated over 100 replicates.

The model for MVGREML is $\mathbf{y} = \boldsymbol{\alpha_0} + \mathbf{e}$ and $\mathbf{c} = \boldsymbol{\beta} + \boldsymbol{\varepsilon}$. The model for MRNM in the left panel is $\mathbf{y} = \boldsymbol{\alpha_0} + \boldsymbol{\alpha_1} \times \mathbf{c} + \mathbf{e}$ and $\mathbf{c} = \boldsymbol{\beta} + \boldsymbol{\varepsilon}$. The model for MRNM in the right panel is $\mathbf{y} = \boldsymbol{\alpha_0} + \boldsymbol{\tau_0} + \boldsymbol{\tau_1} \times \mathbf{c}$ and $\mathbf{c} = \boldsymbol{\beta} + \boldsymbol{\varepsilon}$.

Supplementary Table 8. P-values of likelihood ratio tests for model comparisons in UKBB analyses of rank-based inverse normal transformed BMI as the main trait, considering either SMK, NEU or PC1 as a covariate

Index	Model comparison		SMK[a]	NEU[b]	PC1[c]
**Univariate**					
**M1**	H_0: RR-GREML k=0[d]<br>H_1: RR-GREML k=1[d]	$\mathbf{T_1 = \alpha_0 + e}$<br>$\mathbf{T_1 = \alpha_0 + \alpha_1 \cdot c + e}$	2.17E-04	1.23E-03	6.38E-01
**M2**	H_0: Uni-GREML<br>H_1: GCI-GRMEL	$\mathbf{T_1 = \alpha_0 + e}$<br>$\mathbf{T_1 = \alpha_0 + \alpha_1 \cdot c + e}$	1.85E-09	8.88E-01	1.00E+00
**M3**	H_0: Uni-GREML<br>H_1: RNM Full	$\mathbf{T_1 = \alpha_0 + e}$<br>$\mathbf{T_1 = \alpha_0 + \alpha_1 \cdot c + \tau_0 + \tau_1 \cdot c}$	6.61E-86	7.18E-38	9.52E-01
**M4**	H_0: Uni-GREML<br>H_1: RNM R-C	$\mathbf{T_1 = \alpha_0 + e}$<br>$\mathbf{T_1 = \alpha_0 + \tau_0 + \tau_1 \cdot c}$	1.55E-83	1.14E-36	9.70E-01
**M5**	H_0: Uni-GREML<br>H_1: RNM G-C	$\mathbf{T_1 = \alpha_0 + e}$<br>$\mathbf{T_1 = \alpha_0 + \alpha_1 \cdot c + e}$	2.47E-75	2.18E-35	8.00E-01
**M6**	H_0: RNM G-C<br>H_1: RNM Full	$\mathbf{T_1 = \alpha_0 + \alpha_1 \cdot c + e}$<br>$\mathbf{T_1 = \alpha_0 + \alpha_1 \cdot c + \tau_0 + \tau_1 \cdot c}$	1.32E-13	3.62E-05	8.83E-01
**M7**	H_0: RNM R-C<br>H_1: RNM Full	$\mathbf{T_1 = \alpha_0 + \tau_0 + \tau_1 \cdot c}$<br>$\mathbf{T_1 = \alpha_0 + \alpha_1 \cdot c + \tau_0 + \tau_1 \cdot c}$	2.11E-05	6.91E-04	7.29E-01
**Multivariate**					
**M8**	H_0: MVGREML<br>H_1: MRNM Full	$\mathbf{T_1 = \alpha_0 + e \quad , T_2 = \beta + \varepsilon}$<br>$\mathbf{T_1 = \alpha_0 + \alpha_1 \cdot c + \tau_0 + \tau_1 \cdot c \quad , T_2 = \beta + \varepsilon}$	5.67E-159	3.21E-36	9.50E-01
**M9**	H_0: MVGREML<br>H_1: MRNM R-C	$\mathbf{T_1 = \alpha_0 + e \quad , T_2 = \beta + \varepsilon}$<br>$\mathbf{T_1 = \alpha_0 + \tau_0 + \tau_1 \cdot c \quad , T_2 = \beta + \varepsilon}$	1.50E-159	1.31E-35	9.61E-01
**M10**	H_0: MVGREML<br>H_1: MRNM G-C	$\mathbf{T_1 = \alpha_0 + e \quad , T_2 = \beta + \varepsilon}$<br>$\mathbf{T_1 = \alpha_0 + \alpha_1 \cdot c + e \quad , T_2 = \beta + \varepsilon}$	4.79E-121	2.06E-34	9.28E-01
**M11**	H_0: MRNM G-C<br>H_1: MRNM Full	$\mathbf{T_1 = \alpha_0 + \alpha_1 \cdot c + e \quad , T_2 = \beta + \varepsilon}$<br>$\mathbf{T_1 = \alpha_0 + \alpha_1 \cdot c + \tau_0 + \tau_1 \cdot c \quad , T_2 = \beta + \varepsilon}$	3.51E-41	1.44E-04	7.58E-01
**M12**	H_0: MRNM R-C<br>H_1: MRNM Full	$\mathbf{T_1 = \alpha_0 + \tau_0 + \tau_1 \cdot c \quad , T_2 = \beta + \varepsilon}$<br>$\mathbf{T_1 = \alpha_0 + \alpha_1 \cdot c + \tau_0 + \tau_1 \cdot c \quad , T_2 = \beta + \varepsilon}$	3.63E-03	1.99E-03	7.20E-01

[a]SMK: Pack years of smoking.

[b]NEU: Neuroticism score treated as continuous variable.

[c]The first principal component provided by UK Biobank.

[d]Samples used in the respective model were arbitrarily stratified into four different levels according to covariates, SMK, NEU and PC1. Residual variance was estimated in each level for RR-GREML whereas GCI-GREML assumes homogeneous residual variance across the four groups and estimates a single residual variance.

Supplementary Table 9. Estimated variance components and standard errors of BMI when fitting both SMK and NEU simultaneously as multiple covariates in RNM[a].

	**var(τ_0)**	**var(τ_1)**	**cov(τ_0,τ_1)**	**var(τ_1)**	**cov(τ_0,τ_1)**	**var(α_0)**	**var(α_1)**	**cov(α_0, α_1)**	**var(α_1)**	**cov(α_0, α_1)**
		SMK		NEU			SMK		NEU	
**Est**	16.19	-0.15	0.71	0.05	0.49	4.92	0.44	-0.14	0.26	0.38
**SE**	0.23	0.17	0.13	0.18	0.11	0.17	0.15	0.11	0.13	0.11

[a] $y_i = \alpha_{i0} + \alpha_{i11} \times SMK_i + \alpha_{i21} \times NEU_i + \tau_{i0} + \tau_{i11} \times SMK_i + \tau_{i21} \times NEU_i$

Supplementary Table 10. Statistical tests for the difference between residual variances for BMI estimated from MRNM[a] and MVGREML[b]

	Difference[c]	SE[d]	Difference in%	SE of Difference in %	h^2 (MVGREML)	h^2 (MRNM)	P[e]
**SMK**	-0.245	0.095	1.456	0.564	0.220	0.217[f]	9.827E-03
**NEU**	-0.339	0.125	2.062	0.759	0.227	0.231	6.564E-03
**PC1**	0.017	0.088	-0.101	0.524	0.227	0.227	8.469E-01

[a]Alternative hypothesis model (H_1) of M8 in Table 1

[b]Null hypothesis model (H_0) of M8 in Table 1

[c]Difference = the residual variance estimated from MRNM – the residual variance estimated from MVGREML.

[d]Standard error of the difference was calculated based on the theory in Supplementary Note.

[e]P value was obtained based a two-tailed Wald test using the difference of residual variances and its SE.

[f]In this analysis, h^2 was decreased because the covariance terms, cov($\boldsymbol{\alpha_1}$, $\boldsymbol{\beta}$) and cov($\boldsymbol{\tau_1}$, $\boldsymbol{\varepsilon}$), captured some of the main genetic variance in MRNM such that the estimated main genetic variance was lower than that in MVGREML that did not parameterize such covariance components. When omitting cov($\boldsymbol{\alpha_1}$, $\boldsymbol{\beta}$) and cov($\boldsymbol{\tau_1}$, $\boldsymbol{\varepsilon}$) from MRNM, the main genetic variance is similar between MRNM and MVGREML. For a more fair comparison, please see Table 2.

Supplementary Table 11. Computational requirements of each model[a].

	N=7000		N=60000	
**Model**	RAM	Wall time	RAM	Wall time
**RNM null**	1.2GB	4min	50.1 GB	2.5h
**RNM R-C**	3.7GB	10min	328.3 GB	6.0h
**RNM G-C**	2.7 GB	9mim	213.8 GB	4.75h
**RNM Full**	4.1 GB	10min	328.3 GB	10h
**MRNM Null**	2.0 GB	7min	146.0 GB	30h
**MRNM R-C**	12.4 GB	55min	1015.9 GB	277h
**MRNM G-C**	9.1 GB	10min	737.7 GB	100h
**MRNM Full**	12.4 GB	67min	1015.9 GB	304h

[a]Those analyses were run on 14 CPUs with each2.6 GHz CPUs. When increasing the number of CPUs, the computational efficiency will be further increased (e.g. by a factor of 2-fold when using a 4-fold higher number of CPUs). To load input files including relationship matrices (for which parallel computing is not allowed), MTG2 takes few seconds with a sample size of 7000, or takes approximately one hour with a sample size of 60000.

Supplementary Table 12. Estimated variance components when an assumption of constant SNP variance across minor allele frequency is violated

		$var(\tau_0)$	$var(\varepsilon)$	$cov(\tau_0,\varepsilon)$	$var(\alpha_0)$	$var(\alpha_1)$	$cov(\alpha_0,\alpha_1)$	$var(\beta)$	$cov(\alpha_0,\beta)$	$cov(\alpha_1,\beta)$
**TRUE**		1	1	0.3	1	0.25	0.05	1	0.5	0
**MRNM G-C**	Est	0.946	0.952	0.273	1.060	0.250	0.051	1.046	0.534	0.003
	SE	0.011	0.008	0.008	0.011	0.002	0.002	0.008	0.008	0.002
**MVGREML**	Est	1.451	0.952	0.275	1.054			1.046	0.532	
	SE	0.011	0.008	0.008	0.012			0.008	0.008	

One hundred replicates of data were simulated with the presence of genotype-covariate interaction and correlation, and residual-covariate correlation. Simulation was based QCed ARIC data consisting of 7,263 individuals and 583,058 SNPs. The model was specified as $\mathbf{y} = \boldsymbol{\alpha_0} + \boldsymbol{\alpha_1}\times\mathbf{c} + \mathbf{e}$ with $\mathbf{c} = \boldsymbol{\beta} + \boldsymbol{\varepsilon}$, all effects drawn from a multivariate normal distribution, where the variance-covariance structure of $\boldsymbol{\alpha_0}$, $\boldsymbol{\beta}$, and $\boldsymbol{\alpha_1}$ (in this order) was $\begin{bmatrix} 1 & 0.5 & 0.05 \\ 0.5 & 1 & 0 \\ 0.05 & 0 & 0.25 \end{bmatrix}$ and that of $\mathbf{e}$ and ε was $\begin{bmatrix} 1 & 0.3 \\ 0.3 & 1 \end{bmatrix}$. We simulated genetic variance due to each causal variant as $var(SNP_i) \propto [p(1-p)]^s$ with the scale parameter s = 0 and applied estimation models (MRNM G-C and MVGREML) that assumes a constant SNP variance across MAF, i.e. the scale parameter s = -1 in estimating a genomic relationship matrix (the standard approach)[1-3].

Estimated genetic variance and SNP-heritability may be biased if an assumption of the variance due to causal variants across different MAF spectrums is violated[1-3]. As expected and agreed with previous studies[2] , the genetic variance ($\mathrm{var}(\boldsymbol{\alpha_0})$, $\mathrm{var}(\boldsymbol{\beta})$) and covariance ($\mathrm{cov}(\boldsymbol{\alpha_0}, \boldsymbol{\beta})$) were slightly overestimated. However, we found that the estimated interaction effects were robust to the assumption violation (when estimation model is different from the true model). Nonetheless, whether there was the assumption violation or not, it was similarly observed that a reduced model (e.g. MVGREML) estimated a much higher residual variance while estimating a similar genetic variance, compared to the MRNM G-C model (estimated heritability was $\mathrm{var}(\alpha_0) / [\mathrm{var}(\alpha_0) + \mathrm{var}(\tau_0)] = 0.53$ and 0.42 for MVGREML and MRNM G-C, respectively).

Supplementary Table 13. Type I error rates with and without rank-based inverse normal transformation based on simulations of non-normal variables

Pheno	Shape in Gamma	NULLvsGC	NULLvsRC	RCvsFULL	GCvsFULL	NULLvsFULL	Kurtosis	Skewness
**RINT**	0.5	0.054	0.056	0.076	0.086	0.084	2.99	0
**RAW**	0.5	0.294	0.324	0.096	0.102	0.28	6.01	1
**RINT**	1	0.054	0.062	0.07	0.062	0.07	2.99	0
**RAW**	1	0.168	0.198	0.066	0.07	0.166	4.50	0.71
**RINT**	1.5	0.06	0.052	0.056	0.056	0.068	2.99	0
**RAW**	1.5	0.122	0.12	0.062	0.06	0.112	4.00	0.58
**RINT**	2	0.046	0.044	0.068	0.078	0.048	2.99	0
**RAW**	2	0.102	0.116	0.052	0.074	0.088	3.76	0.50

Five hundred replicates of data were simulated under a null model that assumed no genotype-covariate correlation and no interaction. Simulation was based QCed ARIC data that have 7,263 individuals and 583,058 SNPs. The model is specified as $\mathbf{y} = \boldsymbol{\alpha}_0 + \boldsymbol{\alpha}_1 \times \mathbf{c} + \mathbf{e}$ with $\mathbf{c} = \boldsymbol{\beta} + \boldsymbol{\varepsilon}$, where $\boldsymbol{\alpha}_0$, $\boldsymbol{\beta}$, and $\boldsymbol{\alpha}_1$ (in this order) follows a multivariate normal distribution with mean zero and the variance-covariance structure being $\begin{bmatrix} 1 & 0 & 0 \\ 0 & 1 & 0 \\ 0 & 0 & 0 \end{bmatrix}$ and that between $\mathbf{e}$ and $\boldsymbol{\varepsilon}$ is $\begin{bmatrix} 1 & 0 \\ 0 & 1 \end{bmatrix}$. The residual values are drawn from a gamma distribution with a shape parameter of 0.5, 1, 1.5 or 2. The simulated residual values were further scaled as mean zero and variance 1. For each replicate, we fit Uni-GREML (as null model), RNM G-C, RNM R-C, and RNM Full models with rank-based inverse normal transformed phenotypes (denoted as 'RINT') or with raw phenotypes (denoted as 'RAW'). Kurtosis and skewness were measured for

the simulated phenotypes using functions kurtosis and skewness in R[4]. It was shown that type I error rates were mostly controlled when using rank-based INTed phenotypes although they were inflated when using raw phenotypes. When using raw phenotypes without rank-based INT, the inflation of type I error rates was probably because the sampling variance of estimated interaction variances was increased due to the normality assumption violation, but not because the mean of estimated interaction variances was biased or shifted (as illustrated in Supplementary Figure 22). This larger sampling variance appeared to be controlled by using rank-based INTed phenotypes. It is also noted that the type error rates were not much inflated when using a model comparison between RC vs Full or GC vs Full even when using skewed phenotypes without rank-based INT. This RC vs Full or GC vs Full test appeared to be able to account for inflated estimates in RC or GC model if there was any inflation, which was explicitly used in the real data analysis (M6, M7, M11 or M12 in Table 1 and Supplementary Table 8).

Supplementary Table 14. Variance components estimated based on a simulation with non-zero correlation between two random effects

	$\mathrm{var}(\boldsymbol{\tau_0})$	$\mathrm{var}(\boldsymbol{\alpha_0})$	$\mathrm{var}(\boldsymbol{\alpha_1})$	$\mathrm{cov}(\boldsymbol{\alpha_0}, \boldsymbol{\alpha_1})$	$\mathrm{var}(\boldsymbol{\alpha_2})$	$\mathrm{cov}(\boldsymbol{\alpha_0}, \boldsymbol{\alpha_2})$
**TRUE**	1	1	0.25	0	0.25	0
**Est**	0.981	1.022	0.245	-0.002	0.249	0.002
**SE**	0.011	0.011	0.002	0.002	0.002	0.002
**Fitting one covariate only**						
**Est**	1.475	1.025	0.245	-0.001		
**SE**	0.012	0.012	0.002	0.002		

The models were specified as $\mathbf{y} = \boldsymbol{\alpha_0} + \boldsymbol{\alpha_1} \times \mathbf{c_1} + \boldsymbol{\alpha_2} \times \mathbf{c_2} + \boldsymbol{\tau_0}$ with $\mathbf{c_1} = \boldsymbol{\beta_1} + \boldsymbol{\varepsilon_1}$ and $\mathbf{c_2} = \boldsymbol{\beta_2} + \boldsymbol{\varepsilon_2}$, all effects drawn from a multivariate normal distribution, where variance-covariance structure between $\boldsymbol{\alpha_0}$, $\boldsymbol{\beta_1}$, $\boldsymbol{\beta_2}$, $\boldsymbol{\alpha_1}$ and $\boldsymbol{\alpha_2}$ (in this order) was $\begin{bmatrix} 1 & 0 & 0 & 0 & 0 \\ 0 & 1 & 0 & 0 & 0 \\ 0 & 0 & 1 & 0 & 0 \\ 0 & 0 & 0 & 0.25 & 0.1 \\ 0 & 0 & 0 & 0.1 & 0.25 \end{bmatrix}$ and that between $\boldsymbol{\tau_0}$, $\boldsymbol{\varepsilon_1}$ and $\boldsymbol{\varepsilon_2}$ was $\begin{bmatrix} 1 & 0 & 0 \\ 0 & 1 & 0 \\ 0 & 0 & 1 \end{bmatrix}$. SE were estimated based on 100 replicates. When fitting two uncorrelated covariates simultaneously, all of the estimated variance components were unbiased. When fitting one random effect (α_1) only (assuming that the second covariate, $\mathbf{c_2}$, is missing), the estimated main and interaction variances for the fitted random effect were unbiased although the residual variance ($\mathrm{var}(\boldsymbol{\tau}_0)$) was overestimated due to the unmodelled random effect (as agreed with the results in Figure 5).

Supplementary Table 15. Variance components estimated based on a simulation with non-zero correlation between two covariates

	$\mathrm{var}(\boldsymbol{\tau}_0)$	$\mathrm{var}(\boldsymbol{\alpha}_0)$	$\mathrm{var}(\boldsymbol{\alpha}_1)$	$\mathrm{cov}(\boldsymbol{\alpha}_0, \boldsymbol{\alpha}_1)$	$\mathrm{var}(\boldsymbol{\alpha}_2)$	$\mathrm{cov}(\boldsymbol{\alpha}_0, \boldsymbol{\alpha}_2)$
**TRUE**	1	1	0.25	0	0.25	0
**Est**	1.002	1.000	0.251	-0.001	0.248	-0.003
**SE**	0.013	0.013	0.002	0.002	0.003	0.002
**Fitting one covariate only**						
**Est**	1.462	1.004	0.268	-0.003		
**SE**	0.013	0.013	0.002	0.002		

The models were specified as $\mathbf{y} = \boldsymbol{\alpha}_0 + \boldsymbol{\alpha}_1 \times \mathbf{c}_1 + \boldsymbol{\alpha}_2 \times \mathbf{c}_2 + \boldsymbol{\tau}_0$ with $\mathbf{c}_1 = \boldsymbol{\beta}_1 + \boldsymbol{\varepsilon}_1$ and $\mathbf{c}_2 = \boldsymbol{\beta}_2 + \boldsymbol{\varepsilon}_2$, all effects drawn from a multivariate normal distribution, where variance-covariance structure between $\boldsymbol{\alpha}_0$, $\boldsymbol{\beta}_1$, $\boldsymbol{\beta}_2$, $\boldsymbol{\alpha}_1$ and $\boldsymbol{\alpha}_2$ (in this order) was $\begin{bmatrix} 1 & 0 & 0 & 0 & 0 \\ 0 & 1 & 0.5 & 0 & 0 \\ 0 & 0.5 & 1 & 0 & 0 \\ 0 & 0 & 0 & 0.25 & 0 \\ 0 & 0 & 0 & 0 & 0.25 \end{bmatrix}$ and that between $\boldsymbol{\tau}_0$, $\boldsymbol{\varepsilon}_1$ and $\boldsymbol{\varepsilon}_2$ was $\begin{bmatrix} 1 & 0 & 0 \\ 0 & 1 & 0 \\ 0 & 0 & 1 \end{bmatrix}$. SE were estimated based on 100 replicates. When fitting two correlated covariates simultaneously, the estimates were unbiased. When fitting one random effect, α_1, only (assuming that the second covariate, $\mathbf{c}_2$, is missing), the estimated $\mathrm{var}(\boldsymbol{\alpha}_1)$ could partly capture $\mathrm{var}(\boldsymbol{\alpha}_2)$ because of the correlation between $\mathbf{c}_1$ and $\mathbf{c}_2$.

Supplementary Table 16. Variance components estimated based on a simulation with non-zero correlations both between two covariates and between two random effects

	$var(\tau_0)$	$var(\alpha_0)$	$var(\alpha_1)$	$cov(\alpha_0, \alpha_1)$	$var(\alpha_2)$	$cov(\alpha_0, \alpha_2)$
**TRUE**	1	1	0.25	0	0.25	0
**Est**	0.973	0.994	0.282	0.002	0.280	-0.002
**SE**	0.013	0.013	0.002	0.002	0.003	0.002
**Fitting one covariate only**						
**Est**	1.471	0.995	0.317	-0.0003		
**SE**	0.013	0.013	0.003	0.0023		

The models were specified as $\mathbf{y} = \boldsymbol{\alpha_0} + \boldsymbol{\alpha_1}\times\mathbf{c_1} + \boldsymbol{\alpha_2}\times\mathbf{c_2} + \boldsymbol{\tau_0}$ with $\mathbf{c_1} = \boldsymbol{\beta_1} + \boldsymbol{\varepsilon_1}$ and $\mathbf{c_2} = \boldsymbol{\beta_2} + \boldsymbol{\varepsilon_2}$, all effects drawn from a multivariate normal distribution, where variance-covariance structure between $\boldsymbol{\alpha_0}$, $\boldsymbol{\beta_1}$, $\boldsymbol{\beta_2}$, $\boldsymbol{\alpha_1}$ and $\boldsymbol{\alpha_2}$ (in this order) was $\begin{bmatrix} 1 & 0 & 0 & 0 & 0 \\ 0 & 1 & 0.5 & 0 & 0 \\ 0 & 0.5 & 1 & 0 & 0 \\ 0 & 0 & 0 & 0.25 & 0.1 \\ 0 & 0 & 0 & 0.1 & 0.25 \end{bmatrix}$ and that between $\boldsymbol{\tau_0}$, $\boldsymbol{\varepsilon_1}$ and $\boldsymbol{\varepsilon_2}$ was $\begin{bmatrix} 1 & 0 & 0 \\ 0 & 1 & 0 \\ 0 & 0 & 1 \end{bmatrix}$. SE were estimated based on 100 replicates. When fitting two correlated covariates simultaneously, interaction variances were biased due to unmodeled correlation between α_1 and α_2. When fitting one random effect, α_1, only (assuming that the second covariate, $\mathbf{c}_2$, is missing), the estimated $var(\alpha_1)$ could partly capture $var(\alpha_2)$ because of the correlation between $\mathbf{c}_1$ and $\mathbf{c}_2$ and between α_1 and α_2.

Supplementary Table 17. Variance components estimated with a covariate that is not a modulator itself but correlated with a modulator

	var(τ_0)	var(α_0)	var(α_1)	cov(α_0, α_1)	var(α_2)	cov(α_0, α_2)
**TRUE**	1	1	0.25	0	0	0
**Est**	1.004	0.992	0.249	0.000	0.001	-0.004
**SE**	0.011	0.011	0.002	0.002	0.001	0.001
**Fitting one covariate only**						
**Est**	1.473	0.989	-	-	0.017	-0.004
**SE**	0.012	0.012	-	-	0.002	0.002

The models of simulation were specified as $\mathbf{y} = \boldsymbol{\alpha_0} + \boldsymbol{\alpha_1}\times\mathbf{c_1} + \boldsymbol{\alpha_2}\times\mathbf{c_2} + \boldsymbol{\tau_0}$ with $\mathbf{c_1} = \boldsymbol{\beta_1} + \boldsymbol{\varepsilon_1}$ and $\mathbf{c_2} = \boldsymbol{\beta_2} + \boldsymbol{\varepsilon_2}$, all effects drawn from a multivariate normal distribution, where variance-covariance structure between $\boldsymbol{\alpha_0}$, $\boldsymbol{\beta_1}$, $\boldsymbol{\beta_2}$, $\boldsymbol{\alpha_1}$ and $\boldsymbol{\alpha_2}$ (in this order) was $\begin{bmatrix} 1 & 0 & 0 & 0 & 0 \\ 0 & 1 & 0.5 & 0 & 0 \\ 0 & 0.5 & 1 & 0 & 0 \\ 0 & 0 & 0 & 0.25 & 0 \\ 0 & 0 & 0 & 0 & 0 \end{bmatrix}$ and that between $\boldsymbol{\tau_0}$, $\boldsymbol{\varepsilon_1}$ and $\boldsymbol{\varepsilon_2}$ was $\begin{bmatrix} 1 & 0 & 0 \\ 0 & 1 & 0 \\ 0 & 0 & 1 \end{bmatrix}$. SE were estimated based on 100 replicates. The fitted model was $\mathbf{y} = \boldsymbol{\alpha_0} + \boldsymbol{\alpha_2}\times\mathbf{c_2} + \boldsymbol{\tau_0}$. When fitting a single covariate, $\mathbf{c_2}$, (which was not a modulator but correlated with a modulator, $\mathbf{c_1}$), the estimated var(α_2) could partly capture var(α_1) as expected due to the correlation structure. When fitting two covariates simultaneously (i.e. full information), the estimates of all variance components were unbiased.

Supplementary Table 18. Variance components estimated based on a null simulation model (i.e. no interactions) with two covariates

	$var(\tau_0)$	$var(\alpha_0)$	$var(\alpha_1)$	$cov(\alpha_0, \alpha_1)$	$var(\alpha_2)$	$cov(\alpha_0, \alpha_2)$
**TRUE**	1	1	0	0	0	0
**Est**	1.016	0.989	0.001	0.001	0.000	0.000
**SE**	0.009	0.009	0.001	0.001	0.001	0.001

The models of simulation were specified as $\mathbf{y} = \boldsymbol{\alpha}_0 + \boldsymbol{\alpha}_1 \times \mathbf{c}_1 + \boldsymbol{\alpha}_2 \times \mathbf{c}_2 + \boldsymbol{\tau}_0$ with $\mathbf{c}_1 = \boldsymbol{\beta}_1 + \boldsymbol{\varepsilon}_1$ and $\mathbf{c}_2 = \boldsymbol{\beta}_2 + \boldsymbol{\varepsilon}_2$, all effects drawn from a multivariate normal distribution, where variance-covariance structure between $\boldsymbol{\alpha}_0$, $\boldsymbol{\beta}_1$, $\boldsymbol{\beta}_2$, $\boldsymbol{\alpha}_1$ and $\boldsymbol{\alpha}_2$ (in this order) was $\begin{bmatrix} 1 & 0 & 0 & 0 & 0 \\ 0 & 1 & 0.5 & 0 & 0 \\ 0 & 0.5 & 1 & 0 & 0 \\ 0 & 0 & 0 & 0 & 0 \\ 0 & 0 & 0 & 0 & 0 \end{bmatrix}$ and that between $\boldsymbol{\tau}_0$, $\boldsymbol{\varepsilon}_1$ and $\boldsymbol{\varepsilon}_2$ was $\begin{bmatrix} 1 & 0 & 0 \\ 0 & 1 & 0 \\ 0 & 0 & 1 \end{bmatrix}$. SE were estimated based on 100 replicates. The fitted model was $\mathbf{y} = \boldsymbol{\alpha}_0 + \boldsymbol{\alpha}_1 \times \mathbf{c}_1 + \boldsymbol{\alpha}_2 \times \mathbf{c}_2 + \boldsymbol{\tau}_0$. When there was no interaction, there was no spurious interaction signal even with fitting correlated covariates (a type I error rate of 0.056 (Supplementary Figure 23)). It was already shown that type I error rate was well controlled for a single covariate model (results not shown here).

Supplementary Table 19. P values of likelihood ratio tests in the meta-analyses across the first and second groups (g1 and g2) within UKBB1.

	Likelihood ratio				P value			
	g1	g2	Meta	Full	g1	g2	Meta	Full
				**SMK**				
**M1**	1.26	9.14	10.41	13.82	5.31E-01	1.03E-02	3.41E-02	1.00E-03
**M2**	5.82	12.11	23.48	27.04	1.59E-02	5.02E-04	1.01E-04	1.99E-07
**M3**	129.78	89.02	202.79	13.82	4.34E-27	2.12E-18	9.42E-43	1.89E-49
**M4**	128.57	80.14	208.71	27.04	1.21E-28	3.97E-18	5.04E-44	8.76E-48
**M5**	116.65	78.18	194.83	13.82	4.67E-26	1.06E-17	4.85E-41	1.19E-44
**M6**	13.13	10.85	23.98	27.04	1.41E-03	4.41E-03	8.08E-05	1.35E-07
**M7**	1.21	8.89	10.10	13.82	5.45E-01	1.18E-02	3.88E-02	1.83E-04
**M8**	325.18	315.97	603.26	27.04	3.26E-67	3.08E-65	3.05E-129	1.97E-135
**M9**	321.86	311.25	622.49	13.82	1.84E-69	3.66E-67	2.11E-133	6.10E-137
**M10**	257.24	254.35	501.39	27.04	1.78E-55	7.50E-55	3.36E-107	2.93E-101
**M11**	67.95	61.62	122.07	13.82	1.17E-14	2.65E-13	1.93E-25	2.37E-37
**M12**	3.32	4.73	5.42	27.04	3.45E-01	1.93E-01	2.47E-01	3.26E-02
				**NEU**				
**M1**	0.97	6.05	7.02	13.82	6.15E-01	4.85E-02	1.35E-01	6.82E-04
**M2**	0.02	0.02	0.42	27.04	8.99E-01	8.99E-01	9.80E-01	6.18E-01
**M3**	100.69	138.89	223.19	13.82	7.00E-21	4.88E-29	3.85E-47	1.05E-49
**M4**	100.53	131.79	232.32	27.04	1.48E-22	2.42E-29	4.18E-49	2.36E-48
**M5**	87.19	131.30	218.49	13.82	1.17E-19	3.08E-29	3.95E-46	1.15E-46
**M6**	13.50	7.59	21.09	27.04	1.17E-03	2.25E-02	3.04E-04	7.73E-06
**M7**	0.16	7.10	7.26	13.82	9.23E-01	2.87E-02	1.23E-01	3.77E-04
**M8**	104.96	139.45	214.22	27.04	2.32E-20	1.31E-27	3.28E-45	4.12E-48
**M9**	104.20	132.36	227.89	13.82	1.94E-22	1.68E-28	3.76E-48	2.18E-47
**M10**	88.60	131.63	211.73	27.04	4.39E-19	2.41E-28	1.13E-44	1.17E-45
**M11**	16.36	7.82	19.90	13.82	9.56E-04	5.00E-02	5.23E-04	2.36E-05
**M12**	0.76	7.09	5.65	27.04	8.59E-01	6.90E-02	2.27E-01	1.08E-03
				**PC1**				
**M1**	1.59	2.36	3.95	13.82	4.52E-01	3.07E-01	4.12E-01	7.00E-01
**M2**	0.24	0.00	0.94	27.04	6.26E-01	1.00E+00	9.19E-01	1.00E+00
**M3**	7.42	3.33	5.69	13.82	1.15E-01	5.04E-01	2.23E-01	8.39E-01
**M4**	5.24	1.32	6.56	27.04	7.29E-02	5.17E-01	1.61E-01	5.63E-01
**M5**	1.79	0.56	2.36	13.82	4.08E-01	7.54E-01	6.71E-01	5.02E-01
**M6**	5.63	2.77	8.40	27.04	5.98E-02	2.51E-01	7.80E-02	9.74E-01
**M7**	7.42	3.33	10.76	13.82	2.44E-02	1.89E-01	2.95E-02	8.69E-01
**M8**	9.00	3.96	4.27	27.04	1.74E-01	6.82E-01	3.71E-01	8.98E-01
**M9**	6.38	1.93	5.78	13.82	9.46E-02	5.86E-01	2.16E-01	7.09E-01

**M10**	2.24	1.08	1.78	27.04	5.25E-01	7.81E-01	7.76E-01	7.09E-01
**M11**	6.76	2.88	6.83	13.82	7.99E-02	4.11E-01	1.45E-01	8.39E-01
**M12**	2.62	2.03	2.72	27.04	4.54E-01	5.66E-01	6.06E-01	8.40E-01

Supplementary Table 20. P values of likelihood ratio tests for different models in the meta-analyses across UKBB1 and UKBB2.

	Likelihood ratio				P value			
	G1[a]	G2[b]	G0[c]	Meta	G1	G2	G0	Meta
				SMK				
**M1**	13.02	2.43	13.82	29.27	1.49E-03	2.97E-01	1.00E-03	5.41E-05
**M2**	5.19	5.47	27.04	46.33	2.27E-02	1.93E-02	1.99E-07	2.55E-08
**M3**	123.84	93.55	233.92	425.75	8.09E-26	2.32E-19	1.89E-49	8.12E-89
**M4**	111.12	91.04	216.71	418.87	7.43E-25	1.70E-20	8.76E-48	2.45E-87
**M5**	122.86	90.06	202.29	415.20	2.10E-27	2.78E-20	1.19E-44	1.50E-86
**M6**	0.98	3.49	31.63	36.10	6.13E-01	1.75E-01	1.35E-07	2.64E-06
**M7**	12.72	2.51	17.21	32.44	1.73E-03	2.86E-01	1.83E-04	1.34E-05
**M8**	606.13	592.52	642.05	1776.12	1.11E-127	9.57E-125	1.97E-135	0.00E+00
**M9**	600.76	586.60	633.29	1802.78	6.90E-130	8.09E-127	6.10E-137	0.00E+00
**M10**	470.79	472.08	468.67	1394.42	1.02E-101	5.35E-102	2.93E-101	3.91E-298
**M11**	135.34	120.44	173.38	415.62	3.81E-29	6.21E-26	2.37E-37	1.22E-86
**M12**	5.37	5.92	8.76	15.00	1.47E-01	1.16E-01	3.26E-02	2.03E-02
				NEU				
**M1**	5.12	7.84	14.58	27.54	7.73E-02	1.98E-02	6.82E-04	1.15E-04
**M2**	5.19	5.47	27.04	46.33	2.27E-02	1.93E-02	1.99E-07	2.55E-08
**M3**	239.63	144.21	235.10	591.22	1.11E-50	3.54E-30	1.05E-49	1.83E-124
**M4**	232.56	136.82	219.33	588.71	3.16E-51	1.95E-30	2.36E-48	6.35E-124
**M5**	190.94	123.08	211.56	525.57	3.46E-42	1.88E-27	1.15E-46	2.61E-110
**M6**	48.70	21.13	23.54	93.37	2.66E-11	2.58E-05	7.73E-06	6.02E-18
**M7**	7.07	7.39	15.77	30.23	2.91E-02	2.48E-02	3.77E-04	3.55E-05
**M8**	243.16	148.88	235.94	576.50	1.19E-49	1.33E-29	4.12E-48	2.73E-121
**M9**	235.84	136.76	219.83	577.98	7.53E-51	1.89E-29	2.18E-47	1.31E-121
**M10**	194.54	126.06	211.83	518.29	6.38E-42	3.82E-27	1.17E-45	9.66E-109
**M11**	48.62	22.82	24.12	86.52	1.57E-10	4.39E-05	2.36E-05	1.60E-16
**M12**	7.32	12.13	16.11	29.15	6.25E-02	6.96E-03	1.08E-03	5.70E-05
				PC1				
**M1**	11.04	1.67	0.71	13.42	4.01E-03	4.34E-01	7.00E-01	3.69E-02
**M2**	5.19	5.47	27.04	46.33	2.27E-02	1.93E-02	1.99E-07	2.55E-08
**M3**	10.26	1.96	1.43	7.58	3.62E-02	7.43E-01	8.39E-01	2.70E-01
**M4**	1.94	0.44	1.15	3.53	3.80E-01	8.03E-01	5.63E-01	7.41E-01
**M5**	2.22	0.63	1.38	4.23	3.30E-01	7.28E-01	5.02E-01	6.46E-01
**M6**	8.04	1.33	0.05	9.42	1.79E-02	5.15E-01	9.74E-01	1.51E-01
**M7**	8.33	1.52	0.28	10.13	1.56E-02	4.67E-01	8.69E-01	1.19E-01

**M8**	12.16	2.19	2.23	6.10	5.84E-02	9.02E-01	8.98E-01	4.12E-01
**M9**	2.82	0.63	1.39	2.66	4.19E-01	8.90E-01	7.09E-01	8.50E-01
**M10**	2.25	0.73	1.38	2.28	5.21E-01	8.65E-01	7.09E-01	8.92E-01
**M11**	9.91	1.46	0.84	8.97	1.94E-02	6.93E-01	8.39E-01	1.75E-01
**M12**	9.34	1.56	0.84	8.52	2.51E-02	6.68E-01	8.40E-01	2.02E-01

[a]A random half group of the second release of UK Biobank

[b]The remaining individuals (the other half) of the second release of UK Biobank

[c]The first release of UK Biobank

Supplementary Notes

Supplementary Note 1. Note for more general equations with a higher order

Reaction norm model (RNM)

To account for phenotypic plasticity and norms of reaction in response to different covariate or environmental conditions among samples[5,6], the dependent variable for individual *i* can be modelled as

$$y_i = b_i + g_i + e_i = b_i + \sum_{z=0}^{k} \alpha_{iz} \cdot c_i^z + e_i \quad (1)$$

where y_i is the phenotypic observation, b_i represents fixed effects, g_i is the random genetic effect, α_{iz} is the *z*th order of random regression coefficients ($z = 0 \sim k$), c_i is the covariate value, and e_i is the residual effect for the *i*th individual. Assuming that each individual has unique covariate value, the variance-covariance matrix of observed phenotypes (y_i) is

$$\mathrm{var}(\mathbf{y}) = \begin{bmatrix} \mathbf{Z}_1\mathbf{A}\sigma_{g_1}^2\mathbf{Z}_1' + \mathbf{Z}_1\mathbf{I}\sigma_{e_1}^2\mathbf{Z}_1' & \cdots & \mathbf{Z}_1\mathbf{A}\sigma_{g_{1,N}}\mathbf{Z}_N' + \mathbf{Z}_1\mathbf{I}\sigma_{e_{1,N}}\mathbf{Z}_N' \\ \vdots & \ddots & \vdots \\ \mathbf{Z}_\mathrm{N}\mathbf{A}\sigma_{g_{1,N}}\mathbf{Z}_1' + \mathbf{Z}_\mathrm{N}\mathbf{I}\sigma_{e_{1,N}}\mathbf{Z}_1' & \cdots & \mathbf{Z}_N\mathbf{A}\sigma_{g_N}^2\mathbf{Z}_\mathrm{N}' + \mathbf{Z}_N\mathbf{I}\sigma_{e_N}^2\mathbf{Z}_N' \end{bmatrix},$$

where **A** is the *N* x *N* genomic relationship matrix based on genome-wide SNP information, $\mathbf{Z}_\mathrm{i}$ is an incidence matrix for g_i, and **I** is an *N* x *N* identity matrix. The terms $\sigma_{g_i}^2$ and $\sigma_{e_i}^2$ denote the genetic and residual variances at the *i*th covariate level. The terms $\sigma_{g_{i,j}}$ and $\sigma_{e_{i,j}}$ indicate the genetic and residual covariance between the *i*th and *j*th covariate levels (*i*=1, … , *N*, and *j*=1, … , *N*), respectively.[7] The random genetic and residual effect are assumed following a normal distribution with mean as zero and variance as $\mathbf{A}\sigma_g^2$ and $\mathbf{I}\sigma_e^2$. The random genetic effect, g_i, can be regressed on the covariate gradient (reaction norm), which can be efficiently modelled with

random regression coefficients. The variance-covariance matrix of random regression coefficients (**K**) is

$$\mathbf{K} = \mathrm{cov}(\boldsymbol{\alpha}_z, \boldsymbol{\alpha}_l) = \begin{bmatrix} \mathrm{var}(\boldsymbol{\alpha}_0) & \cdots & \mathrm{cov}(\boldsymbol{\alpha}_0, \boldsymbol{\alpha}_\mathrm{k}) \\ \vdots & \ddots & \vdots \\ \mathrm{cov}(\boldsymbol{\alpha}_0, \boldsymbol{\alpha}_\mathrm{k}) & \cdots & \mathrm{var}(\boldsymbol{\alpha}_\mathrm{k}) \end{bmatrix}$$

where $\boldsymbol{\alpha}_z$ and $\boldsymbol{\alpha}_l$ are the *z*th and *l*th order random regression coefficients ($z = l = 0 \sim k$). The genetic (co)variance matrix of genetic effects between *N* individuals or *N* covariate values (because each individual has unique covariate value) is a function of random regression coefficients and polynomials, which can be expressed as

$$\mathbf{V_g} = \boldsymbol{\Phi}\mathbf{K}\boldsymbol{\Phi}' = \begin{bmatrix} \sigma^2_{g_1} & \cdots & \sigma_{g_{1,N}} \\ \vdots & \ddots & \vdots \\ \sigma_{g_{N,1}} & \cdots & \sigma^2_{g_N} \end{bmatrix}$$

where $\boldsymbol{\Phi}$ is the $N \times (k+1)$ matrix of polynomials evaluated given *N* covariate values as c_i^z with $z = 0 \sim \mathrm{k}$. For example, with an order k=1, the polynomial matrix is $\boldsymbol{\Phi_i} = [\boldsymbol{c}_i^0, \boldsymbol{c}_i^1]$.

Given that this model does not explicitly parameterise the correlation between y_i and c_i, it naively assumes that y_i and c_i are uncorrelated. For this reason, this model is also referred to as a genotype-covariate interaction (G-C interaction) model.

Multivariate reaction norm model (MRNM)

The naïve assumption of the univariate RNM (or G-C interaction model) that y_i and c_i are uncorrelated is often violated. In a more proper model, the covariate value for individual *i* is decomposed as $c_i = \mu_i + \beta_i + \varepsilon_i$, where μ_i is fixed effects, β_i is the random genetic effect, and ε_i is the residual effect. When considering the main response (**y**) and covariate (**c**) jointly in a multivariate model, the variance-covariance matrix of observed phenotypes y_i and c_i is

$\mathrm{cov}(\mathbf{y},\mathbf{c}) =$

$$\begin{bmatrix} \mathbf{Z}_1\mathbf{A}\sigma^2_{g_1}\mathbf{Z}_1' + \mathbf{Z}_1\mathbf{I}\sigma^2_{e_1}\mathbf{Z}_1' & \cdots & \mathbf{Z}_1\mathbf{A}\sigma_{g_{1,N}}\mathbf{Z}_N' + \mathbf{Z}_1\mathbf{I}\sigma_{e_{1,N}}\mathbf{Z}_N' & \mathbf{Z}_1\mathbf{A}\sigma_{g_{1,\beta}}\mathbf{Z}_c' + \mathbf{Z}_1\mathbf{I}\sigma_{e_{1,\varepsilon}}\mathbf{Z}_c' \\ \vdots & \ddots & \vdots & \vdots \\ \mathbf{Z}_\mathrm{N}\mathbf{A}\sigma_{g_{1,N}}\mathbf{Z}_1' + \mathbf{Z}_\mathrm{N}\mathbf{I}\sigma_{e_{1,N}}\mathbf{Z}_1' & \cdots & \mathbf{Z}_N\mathbf{A}\sigma^2_{g_N}\mathbf{Z}_\mathrm{N}' + \mathbf{Z}_N\mathbf{I}\sigma^2_{e_N}\mathbf{Z}_N' & \mathbf{Z}_N\mathbf{A}\sigma_{g_N,\beta}\mathbf{Z}_c' + \mathbf{Z}_N\mathbf{I}\sigma_{e_N,\varepsilon}\mathbf{Z}_c' \\ \mathbf{Z}_c\mathbf{A}\sigma_{g_1,\beta}\mathbf{Z}_1' + \mathbf{Z}_\mathrm{c}\mathbf{I}\sigma_{e_1,\varepsilon}\mathbf{Z}_1' & \cdots & \mathbf{Z}_c\mathbf{A}\sigma_{g_N,\beta}\mathbf{Z}_N' + \mathbf{Z}_\mathrm{c}\mathbf{I}\sigma_{e_N,\varepsilon}\mathbf{Z}_N' & \mathbf{Z}_c\mathbf{A}\sigma^2_\beta\mathbf{Z}_c' + \mathbf{Z}_c\mathbf{I}\sigma^2_\varepsilon\mathbf{Z}_c' \end{bmatrix}$$

where $\mathbf{Z}_\mathrm{c}$ is an incidence matrix for the vector of the random genetic and residual effects, $\boldsymbol{\beta}$ and $\boldsymbol{\varepsilon}$, underlying **c**. The genetic and residual variances of covariate **c** are denoted as σ^2_β and σ^2_ε, respectively. The terms $\sigma_{g_i,\beta}$ and $\sigma_{e_i,\varepsilon}$ indicate the genetic and residual covariance between main trait and covariate at the *i*th covariate levels (*i* =1,…, *N*), respectively. The random genetic and residual effects of y are the same as defined above. The random genetic and residual effect of **c** are assumed following a normal distribution with mean as zero and variance as $\mathbf{A}\sigma^2_\beta$ and $\mathbf{I}\sigma^2_\varepsilon$.The genetic (co)variance matrix of individual genetic effects in the multivariate model can be written as

$$\mathbf{V}_{\mathrm{g},\beta} = \begin{bmatrix} \boldsymbol{\Phi}\mathbf{K}_\mathbf{y}\boldsymbol{\Phi}' & \boldsymbol{\Phi}\mathbf{K}_{\mathbf{y},\mathbf{c}} \\ \mathbf{K}_{\mathbf{y},\mathbf{c}}'\boldsymbol{\Phi}' & \mathrm{var}(\boldsymbol{\beta}) \end{bmatrix} = \begin{bmatrix} \sigma^2_{g_1} & \cdots & \sigma_{g_{1,N}} & \sigma_{g_1,\beta} \\ \vdots & \ddots & \vdots & \vdots \\ \sigma_{g_{N,1}} & \cdots & \sigma^2_{g_N} & \sigma_{g_N,\beta} \\ \sigma_{g_1,\beta} & \cdots & \sigma_{g_N,\beta} & \sigma^2_\beta \end{bmatrix} \qquad (2)$$

where $\mathbf{K}_\mathbf{y}$ is the same as **K** defined above, and $\mathbf{K}_{\mathbf{y},\mathbf{c}}$ consists of the covariance between $\boldsymbol{\beta}$ and the random regression coefficients, that is

$$\mathbf{K}_{\mathrm{y,c}} = \begin{pmatrix} \mathrm{cov}(\boldsymbol{\alpha}_\mathbf{0},\boldsymbol{\beta}) \\ \vdots \\ \mathrm{cov}(\boldsymbol{\alpha}_\mathbf{k},\boldsymbol{\beta}) \end{pmatrix}.$$

The multivariate residual covariance structure is

$$\mathbf{R}_{\mathrm{e},\varepsilon} = \begin{pmatrix} \mathrm{var}(\mathbf{e}) & \mathrm{cov}(\mathbf{e},\boldsymbol{\varepsilon}) \\ \mathrm{cov}(\mathbf{e},\boldsymbol{\varepsilon}) & \mathrm{var}(\boldsymbol{\varepsilon}) \end{pmatrix},$$

where $\mathbf{e}$ is the vector of residual effects for the main phenotypes, assuming that var($\mathbf{e}$) is homogenous across different levels of covariate values, i.e. $\sigma^2_{e_1} = \sigma^2_{e_2} =, \dots, = \sigma^2_{e_N}$, which can be relaxed for the case of heterogeneous residual variances (see the next section), and $\boldsymbol{\varepsilon}$ is the vector of residual effects for the covariate, defined as above, and $\mathrm{var}(\boldsymbol{\varepsilon})$ is the residual variance of the covariate.

This model explicitly parameterises covariance between the random regression coefficients for the main phenotypes and the genetic effects underlying the covariate (i.e. $\mathbf{K}_{\mathrm{y,c}}$), therefore, is referred to as a genotype-covariate correlation and interaction (GCCI) model. Importantly, values for $\mathrm{cov}(\boldsymbol{\alpha}_0, \boldsymbol{\beta})$ or $\mathrm{cov}(\mathbf{e}, \boldsymbol{\varepsilon})$ are often non-negligible. Neglecting these terms can cause confounding between G-C correlation and interaction, thereby generating spurious signals and biased estimates for the interaction. Yet many studies do not account for G-C correlations when estimating and testing G-C interaction[8].

Multivariate reaction norm model (MRNM) accounting for heterogeneous residual variance, i.e. residual-covariate correlation and interaction (RCCI)

The models we described so far assume that the residual variance for the main phenotypes, $\mathrm{var}(\mathbf{e})$, is homogeneous across different values of the covariate. However, it is often possible that residual-covariate (R-C) correlation and interaction exist, resulting in heterogeneous residual variances across different covariate values. To account for this possibility, MRNM can be further generalised as

$$y_i = b_i + g_i + e_i = b_i + \sum_{z=0}^{k} \alpha_{iz} \cdot c_i^z + \sum_{z=0}^{m} \tau_{iz} \cdot c_i^z \qquad (3)$$

where the residual term, e_i, can be also regressed on the covariate gradient, modelled with the random regression coefficients τ_{iz} and the *z*th order polynomial of the covariate (*z* = 0 ~ *m*). The variance-covariance structure of the genetic effect for this model is the same as for the multivariate reaction norm model described in Eq. (2) in the previous section. The multivariate residual covariance structure in this generalised MRNM becomes

$$\mathbf{R}_{\mathrm{e},\varepsilon} = \begin{bmatrix} \mathbf{\Phi}\mathbf{M}_{\mathbf{y}}\mathbf{\Phi}' & \mathbf{\Phi}\mathbf{M}_{\mathbf{y,c}} \\ \mathbf{M}'_{\mathbf{y,c}}\mathbf{\Phi}' & \mathrm{var}(\boldsymbol{\varepsilon}) \end{bmatrix} = \begin{bmatrix} \sigma^2_{e_1} & \cdots & \sigma_{e_{1,N}} & \sigma_{e_1,\varepsilon} \\ \vdots & \ddots & \vdots & \vdots \\ \sigma_{e_{N,1}} & \cdots & \sigma^2_{e_N} & \sigma_{e_N,\varepsilon} \\ \sigma_{e_1,\varepsilon} & \cdots & \sigma_{e_N,\varepsilon} & \sigma^2_{\varepsilon} \end{bmatrix} \quad (4)$$

where $\mathbf{M_y}$ is the variance and covariance matrix of random regression coefficients for the residual components and can be written as

$$\mathbf{M_y} = \mathrm{cov}(\boldsymbol{\tau}_z, \boldsymbol{\tau}_l) = \begin{pmatrix} \mathrm{var}(\boldsymbol{\tau}_0) & \cdots & \mathrm{cov}(\boldsymbol{\tau}_0, \boldsymbol{\tau}_m) \\ \vdots & \ddots & \vdots \\ \mathrm{cov}(\boldsymbol{\tau}_0, \boldsymbol{\tau}_m) & \cdots & \mathrm{var}(\boldsymbol{\tau}_m) \end{pmatrix},$$

where $\boldsymbol{\tau}_z$ and $\boldsymbol{\tau}_l$ are the *z*th and *l*th order random regression coefficients (*z* = *l* = 0 ~ *k*) for the residual effects. $\mathbf{M_{y,c}}$ is a vector with the covariance between $\boldsymbol{\varepsilon}$ and the random regression coefficients for the residual effects, and can be expressed as

$$\mathbf{M_{y,c}} = \begin{pmatrix} \mathrm{cov}(\boldsymbol{\tau}_0, \boldsymbol{\varepsilon}) \\ \vdots \\ \mathrm{cov}(\boldsymbol{\tau}_m, \boldsymbol{\varepsilon}) \end{pmatrix}.$$

RNM with multiple covariates

RNM can be further extended to include multiple covariates. A model fitting with multiple covariates can be expressed as

$$y_i = b_i + \sum_{j=1}^{x} g_{ij} + e_i = b_i + \sum_{j=1}^{x} \sum_{z=0}^{k_j} \alpha_{ijz} \cdot c_{ij}^z + e_i,$$

where x is the number of random effects, each of which is associated with a unique combination of a relationship matrix and covariate (see below), k_j is the polynomial order for the jth random effect, and α_{ijz} and c_{ij}^{z} are the zth order random regression coefficient and polynomial covariate of the jth random effect for the ith individual. Therefore, this model is a multiple random effects model fitting multiple components[9], but it allows the inclusion of interaction effects, modelled with the random regression coefficients and covariate, for each random effect. As in the original multiple random effects model, it is assumed that there is no correlation between the random effects[10].

The variance-covariance matrix of observed phenotypes (y_i) for this multiple random effects model is

$$\mathrm{var}(\mathbf{y}) = \begin{bmatrix} \sum_{j=1}^{x} \mathbf{Z}_1\mathbf{A}_j\sigma^2_{(g_1)_j}\mathbf{Z}_1' + \mathbf{Z}_1\mathbf{I}\sigma^2_{e_1}\mathbf{Z}_1' & \cdots & \sum_{j=1}^{x} \mathbf{Z}_1\mathbf{A}_j\sigma_{(g_{1,N})_j}\mathbf{Z}_N' + \mathbf{Z}_1\mathbf{I}\sigma_{e_{1,N}}\mathbf{Z}_N' \\ \vdots & \ddots & \vdots \\ \sum_{j=1}^{x} \mathbf{Z}_\mathrm{N}\mathbf{A}_j\sigma_{(g_{1,N})_j}\mathbf{Z}_1' + \mathbf{Z}_\mathrm{N}\mathbf{I}\sigma_{e_{1,N}}\mathbf{Z}_1' & \cdots & \sum_{j=1}^{x} \mathbf{Z}_N\mathbf{A}_j\sigma^2_{(g_N)_j}\mathbf{Z}_\mathrm{N}' + \mathbf{Z}_N\mathbf{I}\sigma^2_{e_N}\mathbf{Z}_N' \end{bmatrix},$$

where $\mathbf{A}_\mathrm{j}$ is the genomic relationship matrix for the jth random effect, $\sigma^2_{(g_i)_j}$ is the genetic variance at the ith covariate level for the jth random effect, $\sigma_{(g_{1,N})_j}$ is, for example, the genetic covariance between the first and the last covariate levels, and other terms are defined as above. As in the RNM fitting with a single covariate, g_{ij} in each random effect ($j=1$~x) can be regressed on the covariate gradient in the same manner. The variance-covariance matrix of random regression coefficients for each random effect ($\mathbf{K}_j$) can be written as

$$\mathbf{K}_j = \mathrm{cov}(\boldsymbol{\alpha}_{jz}, \boldsymbol{\alpha}_{jl}) = \begin{bmatrix} \mathrm{var}(\boldsymbol{\alpha}_{j0}) & \cdots & \mathrm{cov}(\boldsymbol{\alpha}_{j0}, \boldsymbol{\alpha}_{jk_j}) \\ \vdots & \ddots & \vdots \\ \mathrm{cov}(\boldsymbol{\alpha}_{j0}, \boldsymbol{\alpha}_{jk_j}) & \cdots & \mathrm{var}(\boldsymbol{\alpha}_{jk_j}) \end{bmatrix}.$$

Similarly, the genetic (co)variance matrix of individual genetic effects between *N* individuals can be obtained as

$$\mathbf{V}_{\mathrm{g}_j} = \mathbf{\Phi}_j \mathbf{K}_j \mathbf{\Phi}_j' = \begin{bmatrix} \sigma^2_{(g_1)_j} & \cdots & \sigma_{(g_{1,N})_j} \\ \vdots & \ddots & \vdots \\ \sigma_{(g_{1,N})j} & \cdots & \sigma^2_{(g_N)j} \end{bmatrix},$$

where $\mathbf{\Phi}_j$ is the $N \times (k_j + 1)$ matrix of covariate polynomials for the *j*th random effect, and the variance covariance components are defined in the variance-covariance matrix of observed phenotypes above (i.e. var(**y**)). This multiple random effects model fitting with multiple covariates can be feasibly extended to MRNM with GCCI and RCCI although the number of parameters increases exponentially.

All models described above can be fitted using MTG2[7].

Supplementary Note 2. Note for assumption violations

Estimated genetic variance and SNP-heritability may be also biased if an assumption of the equal variance due to causal variants across different minor allele frequency (MAF) spectrums is violated[1-3]. However, it is reported that estimated genetic correlation is robust toward such violation as it is the ratio of the covariance over the variances, which can cancel out the bias[2]. We also observed that estimated random regression coefficients were somewhat robust to the violation of this assumption while the main genetic and residual variances were slightly biased (Supplementary Table 12). It was shown that overestimated residual variance from MVGREML (as also shown in Figure 5) was regardless of the assumption violation (Supplementary Table 12).

To investigate if spurious results could result from violations of normality assumptions, we performed a rank-based inverse normal transformation (INT) for the pre-adjusted phenotype of BMI (Supplementary Table 8) and for simulated non-normal distributed data (Supplementary Table 13 and Supplementary Figures 17 and 18). We found that the bias due to non-normality could be remedied by applying the rank-based INT (Supplementary Figure 18 and Supplementary Table 13), which was also considered in Robinson et al.[8]. Even with the rank-based INT, the significance for the R-C effects (M11) and G-C effects (M12) was not substantially decreased (Table 1 vs. Supplementary Table 8). We also found that the significance for the null versus G-C model comparison (M10) or for the null versus R-C model comparison (M9) still remained for BMI-SMK and BMI-NEU (Table 1 vs. Supplementary Table 8). Taken together, these analyses suggest that our results from the real data analyses cannot be attributed to violation of assumptions of normality.

Another caveat is the assumption about negligible correlation between two random effects (see section 'RNM with multiple covariates'), which has been conventionally accepted[10]. When this assumption is violated (Supplementary Tables 14-18), the estimates of interaction and residual variances from RNM could be biased if there was significant correlation between two covariates (Supplementary Table 16). However, the bias was relatively modest considering the substantial correlation between the two random effects used in the simulation (correlation between $\boldsymbol{\alpha}_1$ and $\boldsymbol{\alpha}_2$ = 0.4 in Supplementary Table 16). In the real data analyses, there were no remarkable changes to estimated interaction variances between single and two covariate models (H1 of M6 in Supplementary Data 6 vs. Supplementary Table 9), showing that there was little or no correlation between the random effects. When there was no correlation between random effects,

estimates were always unbiased (Supplementary Tables 14 and 15) and type I error rates were controlled (Supplementary Table 18). When covariate information is not available, overall interaction effects were not fully captured as expected (Supplementary Tables 14-16).

Supplementary Note 3. Estimating the sampling error of the difference between estimated residual variance from GREML and RNM

Assuming that the true underlying model is the RNM with $y_i = \alpha_{i0} + \alpha_{i1} \cdot c_i + e_i$ and $c_i = \beta_i + \varepsilon_i$, the residual variance, σ_e^2, can be unbiasedly estimated. However, standard GREML or LDSC may overestimate the residual variance because of confounding from the interaction that is not properly modeled in the GREML or LDSC model, i.e. $y_i = \alpha_{i0} + e_i^*$ and $c_i = \beta_i + \varepsilon_i$, where $e_i^* = \alpha_{i1} \cdot c_i + e_i$.

We are interested in estimating the sampling variance of the difference between estimated residual variance from RNM, $X = \hat{\sigma}_e^2$, and that from GREML or LDSC, $Y = \hat{\sigma}_{e*}^2$. The sampling variance of the difference (σ_d^2) can be expressed as

$$\sigma_d^2 = \sigma_X^2 + \sigma_Y^2 - 2 \cdot \mathrm{cov}(\mathbf{X}, \mathbf{Y}) \quad \text{(C1)}$$

where σ_X^2 is the sampling variance of $\mathbf{X}$ and σ_Y^2 is the sampling variance of $\mathbf{Y}$.

From RNM, the estimated residual variance is $X = \hat{\sigma}_e^2 = E(\hat{e}^2)$, assuming that the mean of estimated residual values is zero. From GREML, the estimated residual variance is $Y = \hat{\sigma}_{e*}^2 = E[(\hat{\boldsymbol{\alpha}}_\mathbf{1} \cdot \mathbf{c} + \hat{\mathbf{e}})^2] = E(\hat{\mathbf{e}}^\mathbf{2}) + E(\hat{\boldsymbol{\alpha}}_\mathbf{1} \cdot \mathbf{c}^2) + 2E(\hat{\boldsymbol{\alpha}}_\mathbf{1} \cdot \mathbf{c} \cdot \hat{\mathbf{e}})$. Therefore, Y can be written as a linear function of X as $\mathbf{Y} = \mathbf{X}b + \boldsymbol{\lambda}$ where b is a regression coefficient (b = 1) and $\boldsymbol{\lambda} = E(\hat{\boldsymbol{\alpha}}_\mathbf{1} \cdot \mathbf{c}^2) +$

$2\mathrm{E}(\hat{\boldsymbol{\alpha}}_{\mathbf{1}} \cdot \mathbf{c} \cdot \hat{\mathbf{e}}) = \mathrm{E}\left(\hat{\boldsymbol{\alpha}}_{\mathbf{1}}^{\mathbf{2}} \cdot \mathbf{c}^{\mathbf{2}}\right)$ is a random variable. The regression coefficient is b = cov (**X**,**Y**) / var(**X**), therefore cov(**X**,**Y**) = var(**X**). Therefore, Eq. (C1) can be rewritten as

$$\sigma_{\mathrm{d}}^2 = \sigma_{\mathrm{Y}}^2 - \sigma_{\mathrm{X}}^2. \quad \text{(C2)}$$

When there is negligible interaction, the GREML model is $\mathrm{y_i} = \alpha_{\mathrm{i0}} + \mathrm{e_i^*}$ as above but with $\mathrm{e_i^*} \approx \mathrm{e_i}$. Because GREML and RNM are a model based on a (linear) stochastic system[11], the values for $\hat{\sigma}_{\mathrm{e}}^2$ can be similar, higher or lower than $\hat{\sigma}_{\mathrm{e}*}^2$ as shown in Supplementary Figures 19 - 21 illustrating that the magnitude and direction of $\hat{\sigma}_{\mathrm{e}}^2$ relative to $\hat{\sigma}_{\mathrm{e}*}^2$ are stochastic.

If $\hat{\sigma}_{\mathrm{e}}^2$ is the same as $\hat{\sigma}_{\mathrm{e}*}^2$ (i.e. $\mathrm{e_i} = \mathrm{e_i^*}$), there is no difference between **X** and **Y**, hence no sampling variance of the difference (σ_{d}^2=0). We show that when the difference between **X** and **Y** decreases, the sampling variance of the difference decreases as well (Supplementary Figure 20).

It is also possible that σ_{X}^2 is higher than σ_{Y}^2 such that the estimated residual variance from RNM can be written as $\mathrm{e_i} = f + \mathrm{e_i^*}$ where f is a stochastic factor inflating σ_{X}^2 in RNM. From RNM, $\mathrm{X} = \hat{\sigma}_{\mathrm{e}}^2 = \mathrm{E}\left[\left(\hat{\mathbf{e}}^* + \hat{\mathbf{f}}\right)^2\right] = \mathrm{E}(\hat{\mathbf{e}}^{*\mathbf{2}}) + \mathrm{E}\left(\hat{\mathbf{f}}^{\mathbf{2}}\right) - 2\mathrm{E}(\hat{\mathbf{e}}^* \cdot \hat{\mathbf{f}})$. From GREML, $\mathrm{Y} = \hat{\sigma}_{\mathrm{e}^*}^2$. Now, **X** can be written as a linear function of **Y** as **X** = **Y**b + **λ** where b is a regression coefficient (b = 1) and **λ** = $\mathrm{E}(\hat{\mathbf{f}}^{\mathbf{2}}) - 2\mathrm{E}(\hat{\mathbf{e}}^* \cdot \hat{\mathbf{f}}) = \mathrm{E}(\hat{\mathbf{f}}^{\mathbf{2}})$ is a random variable. For this, the regression coefficient is b = cov (**X**,**Y**) / var(**Y**), therefore cov(**X**,**Y**) = var(**Y**). And, Eq. (C1) can be now rewritten as

$$\sigma_{\mathrm{d}}^2 = \sigma_{\mathrm{X}}^2 - \sigma_{\mathrm{Y}}^2. \quad \text{(C3)}$$

With (C2) and (C3), it can be written in general

$$\sigma_{\mathrm{d}}^2 = |\sigma_{\mathrm{Y}}^2 - \sigma_{\mathrm{X}}^2|.$$

This was empirically validated in a simulation study (Supplementary Figure 19).

Supplementary Note 4. Meta-analysis approach and validation using UK Biobank data

For very large datasets, our proposed approach may become computationally infeasible (see Supplementary Table 11 for computational requirements). A solution could be to divide the data in various subsets and undertake a meta-analysis. We show that a meta-analysis[12] of GCCI and RCCI results across difference data subsets is useful and reliable (Supplementary Tables 19-20 , Supplementary Data 7-8, Supplementary Figures 14-15). We simulated phenotypes using UKBB1 genotype data and compared results from meta-analysis of multiple sub-samples with results from each individual sub-sample.

As expected, the values of –log10(P) and likelihood ratio in the meta-analyses were larger than those in each single study (Supplementary Figure 14). The power increased further as the number of studies (and the total sample size) increased as shown in Supplementary Figure 14. The correlation between p-values from a meta-analysis based on two groups and p-values from data combining two groups approached to one when the sample size in each group increased to 10K although the regression slope was less than one (Supplementary Figure 15). As expected, with the same sample size, the power of meta-analyses decreased with the number of groups increased (e.g. 10K x 2 vs. 4K x 5 in Supplementary Figure 15) although it was still higher than that from a single group (Supplementary Figure 14). This indicates that our approach combined with meta-analysis can be applied to any sample size, ensuring that the power keeps increasing with further additions to large-scale biobank data. The increased power in meta-analyses was also evident in real data analyses. We randomly divided the UKBB1 data set into two groups of

equal size (33,140 each for SMK, 27,179 each for NEU and PC1) and obtained meta-analysed p-values (Supplementary Table 19) and estimates (Supplementary Data 7). In agreement with the simulation, the meta-analysed p-values greatly improved the power compared to using a single sub-group study (g1 or g2) (Supplementary Table 19).

We further performed meta-analyses across the UKBB1 and the second release of UK Biobank data that excluded the overlapping and highly related samples from UKBB1 (denoted as UKBB2, see Methods). The UKBB2 was used as an independent validation data set (see Methods for more detail). From the meta-analyses, the significance of R-C interaction effects for BMI-SMK and BMI-NEU increased from p-value = 2.37E-37 to p-value = 1.22E-86 and from 2.36E-05 to 1.60E-16, respectively (M11 in Supplementary Table 20). G-C interaction effects for BMI-NEU became more significant and the p-values decreased from 1.08E-03 to 5.70E-05 (M12 in Supplementary Table 20). The meta-analysed estimated variance components are shown in Supplementary Data 8.

Supplementary Note 5. Note for large fixed effects

(M)RNM allows non-zero fixed effects of covariates, for which the main phenotypes are pre-adjusted using a linear model fitting covariates as fixed effects, as shown in the simulation study (Supplementary Data 9). Whether the covariate had a large fixed effect or not, the estimated interaction variances remained unbiased. Some other parameters were shown to be biased especially for estimated covariance terms, cov($\boldsymbol{\tau}_0$, $\boldsymbol{\varepsilon}$) and cov($\boldsymbol{\alpha}_0$, $\boldsymbol{\beta}$) even with adjusted phenotypes (Supplementary Data 9). However, the biases were not substantial unless the covariate had a large fixed effect, i.e. the proportion of the phenotypic variance explained by the

covariate (the coefficient of determination, r^2) was more than 0.11 (Supplementary Data 9). Note that for the real data, the proportion of BMI phenotypic variance explained by SMK was r^2 = 0.01. When a fixed effect fitted as a random effect being an interacting covariate in MRNM by error, the estimated interaction variance component was still unbiased whether phenotypes were adjusted for the covariates or not (Supplementary Data 9).

Supplementary Note 6. Confounders

Alcohol intake (*ALC)*

We summed average weekly alcohol intake (red wine, UK Biobank data field: 1568, champagne plus white wine, UK Biobank data field: 1578, beer plus cider, UK Biobank data field: 1588, spirits, UK Biobank data field: 1598, fortified, UK Biobank data field: 1608), and further combined it with alcohol intake frequency (UK Biobank data field: 1558) to generate ALC. The distribution of ALC is in Supplementary Figure 16.

Townsend deprivation index at recruitment *(TDI)*

The distribution of TDI (UK Biobank data field: 189) is in Supplementary Figure 16. TDI were calculated immediately after participant joining UK Biobank. Based on the preceding national census output areas, each participant is assigned a score corresponding to the output area in which their postcode is located.

Age at interview (Age)

The data field of age at interview in UK Biobank is 21022. Age at interview is derived from date of birth and date of attending an initial assessment centre. It refers to the age of participant on the day they initially attended assessment centre.

Supplementary Reference

1. Speed, D., Hemani, G., Johnson, M.R. & Balding, D.J. Improved heritability estimation from genome-wide SNPs. *American Journal of Human Genetics* **91**, 1011-1021 (2012).
2. Lee, S.H. *et al.* Estimation of SNP heritability from dense genotype data. *American Journal of Human Genetics* **93**, 1151-1155 (2013).
3. Yang, J. *et al.* Genetic variance estimation with imputed variants finds negligible missing heritability for human height and body mass index. *Nature genetics* **47**, 1114 (2015).
4. R Development Core Team. R: A language and environment for statistical computing. (2013).
5. Meyer, K. Estimating covariance functions for longitudinal data using a random regression model. *Genetics Selection Evolution* **30**, 221 (1998).
6. Kirkpatrick, M., Lofsvold, D. & Bulmer, M. Analysis of the inheritance, selection and evolution of growth trajectories. *Genetics* **124**, 979-993 (1990).
7. Lee, S.H. & van der Werf, J. MTG2: An efficient algorithm for multivariate linear mixed model analysis based on genomic information. *Bioinformatics* **32**, 1420-1422 (2016).
8. Robinson, M.R. *et al.* Genotype–covariate interaction effects and the heritability of adult body mass index. *Nature Genetics* **49**, 1174 (2017).
9. Maier, R. *et al.* Joint Analysis of Psychiatric Disorders Increases Accuracy of Risk Prediction for Schizophrenia, Bipolar Disorder, and Major Depressive Disorder. *The American Journal of Human Genetics* **96**, 283-294 (2015).
10. Lynch, M. & Walsh, B. *Genetics and analysis of quantitative traits*, (Sinauer Sunderland, MA, 1998).
11. Neumaier, A. & Groeneveld, E. Restricted maximum likelihood estimation of covariances in sparse linear models. *Genetics Selection Evolution* **30**, 3 (1998).
12. Evangelou, E. & Ioannidis, J.P.A. Meta-analysis methods for genome-wide association studies and beyond. *Nature Reviews Genetics* **14**, 379-389 (2013).
