# Supplementary material for: Genotype–covariate correlation and interaction disentangled by a whole-genome multivariate reaction norm model
Source: Nat Commun. 2019 May 20;10:2239. doi: 10.1038/s41467-019-10128-w (PMC6527612; doi:10.1038/s41467-019-10128-w)
Supplement: Supplementary file 2 — Reporting Summary [file 41467_2019_10128_MOESM2_ESM.pdf]

nature research

Corresponding author(s): S. Hong Lee

Reporting Summary

Nature Research wishes to improve the reproducibility of the work that we publish. This form provides structure for consistency and transparency in reporting. For further information on Nature Research policies, see Authors & Referees and the Editorial Policy Checklist.

Statistical parameters

When statistical analyses are reported, confirm that the following items are present in the relevant location (e.g. figure legend, table legend, main text, or Methods section).

n/a	Confirmed	
☐	☒	The exact sample size (*n*) for each experimental group/condition, given as a discrete number and unit of measurement
☐	☒	An indication of whether measurements were taken from distinct samples or whether the same sample was measured repeatedly
☐	☒	The statistical test(s) used AND whether they are one- or two-sided<br>*Only common tests should be described solely by name; describe more complex techniques in the Methods section.*
☐	☒	A description of all covariates tested
☐	☒	A description of any assumptions or corrections, such as tests of normality and adjustment for multiple comparisons
☐	☒	A full description of the statistics including central tendency (e.g. means) or other basic estimates (e.g. regression coefficient) AND variation (e.g. standard deviation) or associated estimates of uncertainty (e.g. confidence intervals)
☐	☒	For null hypothesis testing, the test statistic (e.g. *F*, *t*, *r*) with confidence intervals, effect sizes, degrees of freedom and *P* value noted<br>*Give P values as exact values whenever suitable.*
☐	☒	For Bayesian analysis, information on the choice of priors and Markov chain Monte Carlo settings
☐	☒	For hierarchical and complex designs, identification of the appropriate level for tests and full reporting of outcomes
☐	☒	Estimates of effect sizes (e.g. Cohen's *d*, Pearson's *r*), indicating how they were calculated
☐	☒	Clearly defined error bars<br>*State explicitly what error bars represent (e.g. SD, SE, CI)*

Our web collection on statistics for biologists may be useful.

Software and code

Policy information about availability of computer code

Data collection	The data used in this study were provided by UK Biobank.
Data analysis	We used MTG2, GCTA, and R to analysis the data in this study.

For manuscripts utilizing custom algorithms or software that are central to the research but not yet described in published literature, software must be made available to editors/reviewers upon request. We strongly encourage code deposition in a community repository (e.g. GitHub). See the Nature Research guidelines for submitting code & software for further information.

Data

Policy information about availability of data

All manuscripts must include a data availability statement. This statement should provide the following information, where applicable:

- Accession codes, unique identifiers, or web links for publicly available datasets
- A list of figures that have associated raw data
- A description of any restrictions on data availability

The simulated data can be obtained from the authors on request. We also used the genotype data of ARIC study under accession code phs000090 in the database of Genotypes and Phenotypes.

Field-specific reporting

Please select the best fit for your research. If you are not sure, read the appropriate sections before making your selection.

☒ Life sciences ☐ Behavioural & social sciences ☐ Ecological, evolutionary & environmental sciences

For a reference copy of the document with all sections, see nature.com/authors/policies/ReportingSummary-flat.pdf

Life sciences study design

All studies must disclose on these points even when the disclosure is negative.

Sample size	We used the UK Biobank data, which initially contained 488,377 individuals and 92,693,895 imputed SNPs across autosomes
Data exclusions	We excluded individuals who met one of the following criteria: 1) does not have white British ancestry, 2) has a genotype missing rate > 0.05, 3) whose reported gender does not match with the gender inferred using genotype data, and 4) has a putative sex chromosome aneuploidy. At the SNP level, we excluded SNPs with an INFO score < 0.6, with a minor allele frequency (MAF) < 0.01, with a Hardy-Weinberg equilibrium P-value <1E-4, or with a call rate < 0.95. For multiple records of the same SNP, we randomly selected one and removed duplicates. We also excluded ambiguous SNPs and only kept HapMap 3 SNPs. In addition, we excluded individual population outliers, namely individuals with the first or second PC outside six standard deviations of its respective population mean. For individuals who were in the first and second waves of UK biobank genotype data, we calculated the discordance rate between imputed genotype of the two versions for each individual and for each SNP, and excluded individuals and SNPs with a discordance rate lager than 0.05. We also excluded one individual randomly from any pair with a genomic relationship larger than 0.05.
Replication	We used simulation to access the type I error rate (500 replicates) and power (100 replicates) of the proposed method. For the analyses based UK Biobank, After the QC, 288,866 individuals and 1,130,918 SNPs remained. Of these remaining individuals 91,472 were from the first wave of UK Biobank (denoted as UKBB1), who were used in the main analyses and the rest of 197,394 individuals were from the second wave of UK Biobank (denoted as UKBB2), who were used in the validation and meta-analyses.
Randomization	In the meta-analyses of for the first wave of UK Biobank, samples were randomly allocated into groups.
Blinding	Blinding was not relevant to our study, Because the data were collected by UK Biobank and the ID of participants has been encrypted.

Reporting for specific materials, systems and methods

Materials & experimental systems

n/a	Involved in the study
☒	☐ Unique biological materials
☒	☐ Antibodies
☒	☐ Eukaryotic cell lines
☒	☐ Palaeontology
☒	☐ Animals and other organisms
☐	☒ Human research participants

Methods

n/a	Involved in the study
☒	☐ ChIP-seq
☒	☐ Flow cytometry
☒	☐ MRI-based neuroimaging

Human research participants

Policy information about studies involving human research participants

Population characteristics	UK Biobank data consist of 500,000 participants aged between 40-69 years in 2006-2010, with ~54% females and 46% males. Approximately 50,000 samples were genotyped on the UK BiLEVE Axiom array and the other 450,000 samples were genotyped on the UK Biobank Axiom array. Genotype were imputed to the whole genome level with IMPUTE3 using UK10K and 1000 Genome Phase 3 as the reference.
Recruitment	The recruitment includes a half million people from all around the UK who are currently aged 40-69 because the age group involves people at risk over the next few decades of developing a wide range of important diseases, conditions and covariates. The phenotype and genotype information is based on extensive baseline questionnaire and physical measures, as well as stored blood and urine samples that allow many different types of assay, incorporated with information from the UK National Health Service.
