# Supplementary material for: Genotype–covariate correlation and interaction disentangled by a whole-genome multivariate reaction norm model
Source: Nat Commun. 2019 May 20;10:2239. doi: 10.1038/s41467-019-10128-w (PMC6527612; doi:10.1038/s41467-019-10128-w)
Supplement: Supplementary file 3 — Description of Additional Supplementary Files [file 41467_2019_10128_MOESM3_ESM.pdf]

Description of Additional Supplementary Files

File Name: Supplementary Data 1

Description:

Supplementary Data 1. Estimates of variance components and standard errors based on simulated data under a residual-covariate interaction (RCI) model in the absence of a genotype-covariate interaction.[a]

[a]The simulated phenotypes were generated as $\mathbf{y} = \boldsymbol{\alpha_0} + \boldsymbol{\tau_0} + \boldsymbol{\tau_1}\times\mathbf{c}$ with $\mathbf{c} = \boldsymbol{\beta} + \boldsymbol{\varepsilon}$, all effects drawn from a multivariate normal distribution. The variance-covariance structure of $\boldsymbol{\alpha_0}$ and $\boldsymbol{\beta}$ is $\begin{bmatrix} 1 & 0 \\ 0 & 1 \end{bmatrix}$ and that of $\boldsymbol{\tau_0}$, $\boldsymbol{\varepsilon}$ and $\boldsymbol{\tau_1}$ is $\begin{bmatrix} 1 & 0 & 0.05 \\ 0 & 1 & 0 \\ 0.05 & 0 & \mathrm{var}(\boldsymbol{\tau_1}) \end{bmatrix}$ with var($\boldsymbol{\tau_1}$) =0.25 in the top panel and 1 in the bottom panel. The standard error was estimated based on 500 replicates. The results are based on the same data as in Supplementary Figure 7.

File Name: Supplementary Data 2

Description:

Supplementary Data 2. Estimates of variance components and standard errors based on simulated data under a genotype-covariate interaction and residual-covariate interaction model[a].

[a]The simulated phenotypes were generated as $\mathbf{y} = \boldsymbol{\alpha}_0 + \boldsymbol{\alpha}_1 \times \mathbf{c} + \boldsymbol{\tau}_0 + \boldsymbol{\tau}_1 \times \mathbf{c}$ with $\mathbf{c} = \boldsymbol{\beta} + \boldsymbol{\varepsilon}$, all effects drawn from a multivariate normal distribution. The variance-covariance structure of $\boldsymbol{\alpha}_0$, $\boldsymbol{\beta}$, and $\boldsymbol{\alpha}_1$ (in this order) is $\begin{bmatrix} 1 & 0 & 0.05 \\ 0 & 1 & 0 \\ 0.05 & 0 & 0.25 \end{bmatrix}$ and that of $\boldsymbol{\tau}_0$, $\boldsymbol{\varepsilon}$, and $\boldsymbol{\tau}_1$ is

$\begin{bmatrix} 1 & 0 & 0.05 \\ 0 & 1 & 0 \\ 0.05 & 0 & \mathrm{var}(\boldsymbol{\tau}_1) \end{bmatrix}$ with $\mathrm{var}(\boldsymbol{\tau}_1) = 0.25$ in the top panel and 1 in the bottom panel.

Standard errors were estimated based on 100 replicates. The results are based on the same data as in Supplementary Figure 8.

File Name: Supplementary Data 3

Description:

Supplementary Data 3. Estimates of variance components and standard errors based on simulated data under a genotype-covariate correlation and residual-covariate correlation and interaction model.[a]

[a]The simulated phenotypes were generated as $\mathbf{y} = \boldsymbol{\alpha}_0 + \boldsymbol{\tau}_0 + \boldsymbol{\tau}_1 \times \mathbf{c}$ with $\mathbf{c} = \boldsymbol{\beta} + \boldsymbol{\varepsilon}$, all effects drawn from a multivariate normal distribution. The variance-covariance structure of $\boldsymbol{\alpha}_0$ and $\boldsymbol{\beta}$ is $\begin{bmatrix} 1 & 0.5 \\ 0.5 & 1 \end{bmatrix}$, and that of $\boldsymbol{\tau}_0$, $\boldsymbol{\varepsilon}$, and $\boldsymbol{\tau}_1$ is $\begin{bmatrix} 1 & 0.3 & 0.05 \\ 0.3 & 1 & 0 \\ 0.05 & 0 & \mathrm{var}(\tau_1) \end{bmatrix}$ with $\mathrm{var}(\boldsymbol{\tau}_1) = 0.25$ in the top panel and 1 in the bottom panel. Standard errors were estimated based on 500 replicates. The results are based on the same data as in Supplementary Figure 9.

File Name: Supplementary Data 4

Description:

Supplementary Data 4. Estimated variance components and standard errors based on simulated data under a genotype-covariate correlation and interaction and residual-covariate correlation and interaction model[a]

The simulated phenotypes were generated as $\mathbf{y} = \boldsymbol{\alpha}_0 + \boldsymbol{\alpha}_1 \times \mathbf{c} + \boldsymbol{\tau}_0 + \boldsymbol{\tau}_1 \times \mathbf{c}$ with $\mathbf{c} = \boldsymbol{\beta} + \boldsymbol{\varepsilon}$, all effects drawn from a multivariate normal distribution. The variance-covariance structure of $\boldsymbol{\alpha}_0$, $\boldsymbol{\beta}$, and $\boldsymbol{\alpha}_1$ (in this order) is $\begin{bmatrix} 1 & 0.5 & 0.05 \\ 0.5 & 1 & 0 \\ 0.05 & 0 & 0.25 \end{bmatrix}$ and that of $\boldsymbol{\tau}_0$, $\boldsymbol{\varepsilon}$, and $\boldsymbol{\tau}_1$ is $\begin{bmatrix} 1 & 0.3 & 0.05 \\ 0.3 & 1 & 0 \\ 0.05 & 0 & \mathrm{var}(\boldsymbol{\tau}_1) \end{bmatrix}$ with $\mathrm{var}(\boldsymbol{\tau}_1) = 0.25$ in the top panel and 1 in the bottom panel. Standard errors were estimated based on 100 replicates. The results are based on the same data as in Supplementary Figure 10.

File Name: Supplementary Data 5

Description:

Supplementary Data 5. Estimated variance components and standard errors based on simulated data under a genotype-covariate correlation and interaction and residual-covariate correlation and interaction model

The simulated phenotypes were generated as $\mathbf{y} = \boldsymbol{\alpha}_0 + \boldsymbol{\alpha}_1 \times \mathbf{c} + \boldsymbol{\tau}_0 + \boldsymbol{\tau}_1 \times \mathbf{c}$ with $\mathbf{c} = \boldsymbol{\beta} + \boldsymbol{\varepsilon}$, all effects drawn from a multivariate normal distribution. The variance-covariance structure of $\boldsymbol{\alpha}_0$, $\boldsymbol{\beta}$, and $\boldsymbol{\alpha}_1$ (in this order) is $\begin{bmatrix} 1 & 0.5 & 0.05 \\ 0.5 & 1 & 0 \\ 0.05 & 0 & 1 \end{bmatrix}$ and that of $\boldsymbol{\tau}_0$, $\boldsymbol{\varepsilon}$, and $\boldsymbol{\tau}_1$ is $\begin{bmatrix} 1 & 0.3 & 0.05 \\ 0.3 & 1 & 0 \\ 0.05 & 0 & \mathrm{var}(\boldsymbol{\tau}_1) \end{bmatrix}$ with $\mathrm{var}(\boldsymbol{\tau}_1) = 0.25$ in the top panel and 1 in the bottom panel. Standard errors were estimated based on 100 replicates. The results are based on the same data as in Supplementary Figure 11.

File Name: Supplementary Data 6

Description:

Supplementary Data 6. Estimated variance components and standard errors of BMI when fitting SMK, NEU or PC1 as a covariate of interest using RR-GREML, GCI-GREML, RNM and MRNM.

File Name: Supplementary Data 7

Description:

Supplementary Data 7. Estimated variance components and standard errors of BMI when fitting SMK, NEU or PC1 as a covariate of interest based on the analysis of each individual study and the meta-analyses across the first and second groups (g1 and g2) within UKBB1.

File Name: Supplementary Data 8

Description:

Supplementary Data 8. Estimated variance components and standard errors of BMI when fitting SMK, NEU or PC1 as a covariate of interest based on the analysis of each individual study and the meta-analyses across UKBB1 and UKBB2.

File Name: Supplementary Data 9

Description:

Supplementary Data 9. Estimated variance components when fixed effects have large main effects

One hundred replicates of data were simulated under each of two scenarios that assumed no genotype-covariate interaction but a residual-covariate interaction of different magnitudes. Simulation was based QCed ARIC data consisting of 7,263 individuals and 583,058 SNPs. The models were specified as $\mathbf{y} = \mathrm{b}\times\mathbf{c} + \boldsymbol{\alpha}_0 + \boldsymbol{\alpha}_1\times\mathbf{c} + \boldsymbol{\tau}_0$ with $\mathbf{c} = \boldsymbol{\beta} + \boldsymbol{\varepsilon}$, all effects drawn from a multivariate normal distribution, where b was the regression coefficient of the fixed effects with a value of 0.1 or 0.5, the variance-covariance structure between $\boldsymbol{\alpha}_0$, $\boldsymbol{\beta}$, and $\boldsymbol{\alpha}_1$ (in this order) was $\begin{bmatrix} 1 & \mathrm{cov}(\alpha_0,\beta) & 0 \\ \mathrm{cov}(\alpha_0,\beta) & 1 & 0 \\ 0 & 0 & var(\alpha_1) \end{bmatrix}$ with

cov($\boldsymbol{\alpha}_0, \boldsymbol{\beta}$)= 0 or 0.5, var($\boldsymbol{\alpha}_1$)=0 or 0.25 and that between $\boldsymbol{\tau}_{\mathbf{0}}, \boldsymbol{\varepsilon}$ was $\begin{bmatrix} 1 & 0 \\ 0 & 1 \end{bmatrix}$. For every replicate, MRNM G-C and MRNM Full models were applied to raw phenotypes ($\mathbf{y}$) or phenotypes adjusted for the covariate ($\mathbf{c}$).
